# Aromatic-bridged and *meso*-*meso*-linked BF_2_-smaragdyrin dimers exhibit fast decays in polar solvents by symmetry-breaking charge transfer

**DOI:** 10.1038/s42004-023-00822-8

**Published:** 2023-02-09

**Authors:** Lingbo Wen, Xian Cao, Seokwon Lee, Ling Xu, Yutao Rao, Seongsoo Kang, Dongho Kim, Atsuhiro Osuka, Jianxin Song

**Affiliations:** 1 Key Laboratory of Chemical Biology and Traditional Chinese Medicine, Ministry of Educational of China, Key Laboratory of the Assembly and Application of Organic Functional Molecules of Hunan Province College of Chemistry and Chemical Engineering, Hunan Normal University, Changsha, 410081 China; 2 Spetroscopy Laboratory for Functional π-Electron Systems and Department of Chemistry, Yonsei University, Seoul, 03722 Korea

**Keywords:** Ligands, Synthetic chemistry methodology, Solar cells

## Abstract

Symmetry-breaking charge transfer is one of the key process in photosynthetic reaction centers and specific artificial optoelectronic devices such as dye-sensitized solar cells. Here we report the synthesis of aromatic-bridged BF_2_-smaragdyrin dimers, *meso*-free BF_2_-smaragdyrin, and its *meso*-*meso*-linked BF_2_-smaragdyrin dimer. The decays of S_1_-states of these dimers are accelerated with an increase in solvent polarity and a decrease in the distance between the two BF_2_-smaragdyrin units, suggesting symmetry-breaking charge transfer. The fluorescence lifetimes of the dimers become shortened in polar solvents. However, ultrafast transient absorption spectroscopy do not detect charge-separated ion pairs. On the basis of these results, we conclude that the decays of the excited states of the BF_2_-smaragdyrin dimers are accelerated by solvation-induced symmetry-breaking charge transfer, depending on the degree of the electronic interaction between the smaragdryin units as a rare case for porphyrinoids. The degree of charge transfer is larger for dimers with larger electronic interactions.

## Abstract (web summary)

Symmetry breaking charge separations have been found to be important for charge transfer in bacteriochlorophyll pairs in photosynthetic reaction centers, but engineering symmetry breaking charge separations in artificial dimers of related chromophores is challenging. Here, the authors report the design and synthesis of aromatic-bridged as well as *meso*-*meso*-linked BF_2_-smaragdyrin dimers that exhibit symmetry-breaking charge transfer properties.

## Introduction

**Fig. 1:**
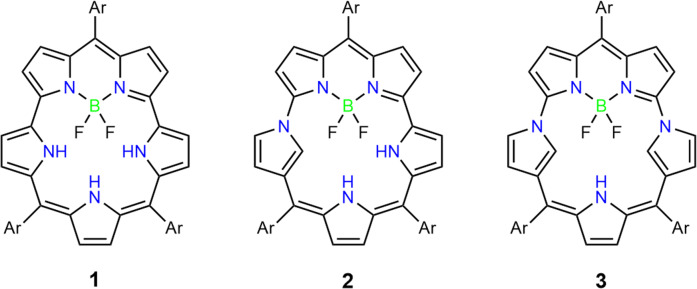
BF_2_–smaragdyrin and Neo-confused BF_2_–smaragdyrins. Structures of BF_2_–smaragdyrin **1**, singly neo-confused BF_2_–smaragdyrin **2**, and doubly neo-confused BF_2_–smaragdyrin **3**.

In recent years, expanded porphyrins have emerged as attractive π-functional molecules in light of versatile electronic properties, rich coordination chemistry, and charming structures^1–5^. Among these, smaragdyrin had been possessing a legendary position, since its presence was reported along with sapphyrin by Woodward but had been long elusive until our first synthesis of 5,10,19-triarylsmaragdyrins in 2018^6^. Instead, Chandrashekar et al have extensively explored the chemistry of core-modified smaragdyrins to demonstrate that these porphyrinoids are promising in many fields including catalysts, molecular hosts, nonlinear optical materials, and coordinating ligands for large metal ions^7–11^. Our synthesis of 5,10,19-triarylsmaragdyrins **1** was based on a double nucleophilic aromatic substitution (S_N_Ar) reaction of *α*,*α*’-dibrominated boron-dipyrromethene’s (BODIPY’s) with a 5,10-diaryltripyrrane^6^. Later, this synthetic protocol was used for the synthesis of singly and doubly Neo-confused smaragdyrins **2** and **3** (Fig. 1)^12^.

In this paper, we report the synthesis of aromatic-bridged BF_2_–smaragdyrin complex dimers **6a–6d** by similar S_N_Ar reactions of aromatic-bridged *α*,*α*’-dibrominated BODIPY dimers **4a–4d** with 5,10-diaryltripyrrane **5**^13,14^. Further, we synthesized *meso*-unsubstituted BF_2_-smaragdyrin **11** and succeeded in its oxidative dimerization with air to give *meso*–*meso*-linked BF_2_–smaragdyrin dimer **12**. While several *meso*–*meso*-linked dimers of core-modified smaragdyrins and analogs were reported^15,16^, this is the first example of *meso*–*meso*-linked smaragdyrin dimer.

Symmetry breaking charge separations (SB-CS) have been recognized to be important in relation to the charge transfer character of the bacteriochlorophyll special pair in the photosynthetic reaction centers, which is considered to trigger the sequential electron-transfer cascade^17,18^. But photoinduced SB-CS of artificial dimers composed of the same chromophores is rare^17–25^, mainly because the energy gaps of the charge separation between the same chromophores are seldom negative enough to allow SB-CS. SB-CS has been never observed for porphyrins, while so many porphyrin dimers and oligomers were synthesized so far. Recently, an interesting example was reported for SB-CS in excitonically coupled subphthalocyanine dimer^26^. In this respect, smaragdyrin is an intriguing porphyrinoid that is active both in reduction and oxidation due to its expanded conjugated π-network. In this paper, we conclude that these smaragdyrin dimers revealed that the excited-state decays are clearly accelerated with an increase of the solvent polarity and a decrease of the distance between the two smaragdyrin units, suggesting the key role of symmetry-breaking charge transfer in their decays as a rare case for porphyrinoids.

## Results

### Synthesis

**Fig. 2:**
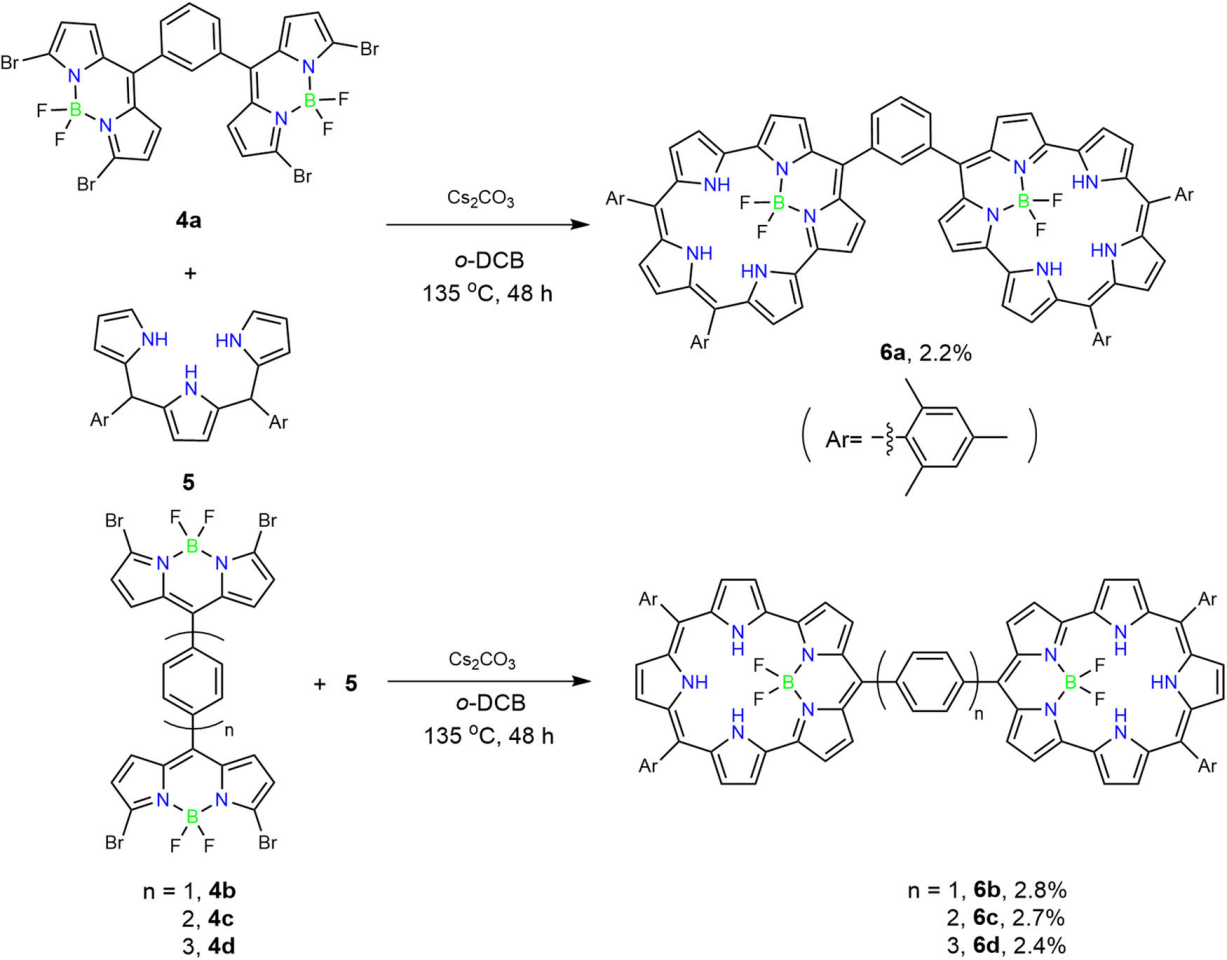
Synthesis of BF_2_–smaragdyrins dimers. Synthesis of **6a–d** through S_N_Ar reactions.

**Fig. 3:**
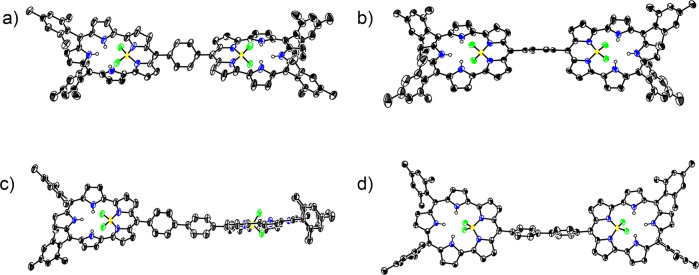
X-ray structures of 6b and 6c. **a** Top view and **b** side view of **6b**. **c** Top view and **d** side view of **6c**. Solvent molecules and hydrogen atoms on carbon have been omitted for clarity.

As the first targets, we synthesized aromatic-spacer-bridged BF_2_–smaragdyrin dimers **6a–6d**. Building blocks **4a–4d** were obtained in moderate yields via a set of transformations including *α*-bromination of the corresponding spacer-bridged dipyrromethene dimers, and oxidation and complexation with BF_2_ unit. Since the solubilities of **4a–4d** were very poor in common solvents and only acceptable in *o*-dichlorobenzene (*o*-DCB), the coupling reactions of **4a–4d** with tripyrromethane **5** were conducted in refluxing *o*-DCB in the presence of an excess amount of cesium carbonate for 48 h. Dimers **6a–6d** were isolated in low but reproducible yields of 2.8, 2.2, 2.7, and 2.4% yield, respectively (Fig. 2). These dimers were characterized by ^1^H and ^13^C NMR spectra and high-resolution matrix-assisted laser desorption ionization time-of-flight (MALDI-TOF) mass spectra (Supplementary Figs. 9, 11, 12, 14, 15, 17, 18, 20, 46–49). The structures of **6b** and **6c** were confirmed by X-ray diffraction analysis (Fig. 3). The crystal structure of **6b** shows that a dihedral angle between the BF_2_–smaragdyrin units and the central 1,4-phenylene bridge is 47.3(2)°. This suggests that the 1,4-phenylene unit may serve as a moderate π-conjugation bridge for the BF_2_-smaragdyrins units. The crystal structure of **6c** shows a dihedral angle of 51.7(2)° for the BF_2_–smaragdyrin units and 4,4-biphenylene unit and a dihedral angle of 31.8(3)° for the two phenylene units in the 4,4’-biphenylene bridge. These structural features suggest that the 4,4’-biphenyl bridge serves as a weaker π-conjugation mediator.

The ^1^H NMR spectra of **6a–6d** in CDCl_3_ are simple, reflecting the symmetric structures. Characteristically, signals due to the inner pyrrolic protons are downfield shifted as compared with the reference monomer **11**. On the other hand, signals of the *β*-protons (labeled as H_1_, H_2_, H_4_, and H_5_) are observed at nearly the same chemical shifts. Signals due to the bridging groups are down-field shifted, reflecting the diatropic ring current of the BF_2_–smaragdyrin ring.

**Fig. 4:**
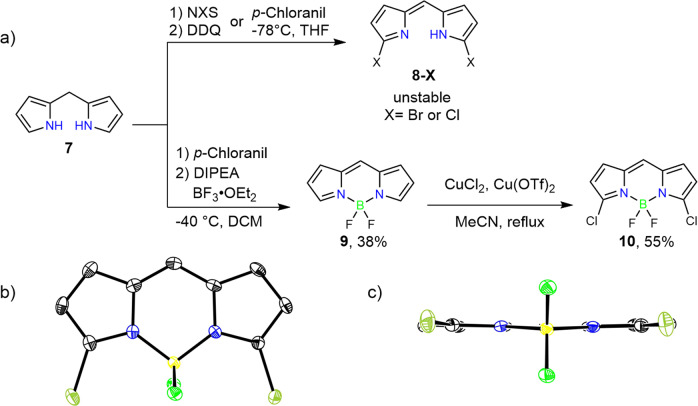
Synthesis and X-ray crystal structures of α, α’-Halogenated meso-free BODIPY 10. **a** Synthesis of α, α’-dichloro meso-free BODIPY **10** and α, α’-dibromo/diiodo meso-free dipyrrin **8-X**. **b** Top view of crystal structure of **10**, and **c** side view of crystal structure of **10**. Solvent molecules and hydrogen atoms on carbon have been omitted for clarity.

As the second target, we synthesized *meso–meso*-linked BF_2_-smaragdyrin dimer **12**. Initially, preparation of *α*,*α*’-dibrominated and dichlorinated *meso*-free BODIPY’s **8**-**Br** and **8**-**Cl** were attempted by halogenations of di(2-pyrroyl)methane **7** with NBS and NCS. But these attempts failed due to the instabilities of **8**-**Br** and **8**-**Cl**. We chose regioselective chlorination of BODIPY’s with CuCl_2_ developed by Jiao and Hall^27^. BODIPY **9** was prepared by oxidation of **7** with *p*-chloranil in CH_2_Cl_2_ at −40 °C under nitrogen followed by reaction with BF_3_•OEt_2_ in 38% yield. Then, the reaction of **9** with CuCl_2_ and Cu(OTf)_2_ under refluxing acetonitrile gave **10** in 55% yield (Fig. 4). The structure of **10** was fully confirmed by ^1^H NMR, ^13^C NMR, HR-MS, and single crystal X-ray diffraction analysis (Fig. 4 and Supplementary Figs. 2, 3, 38, 51).

**Fig. 5:**
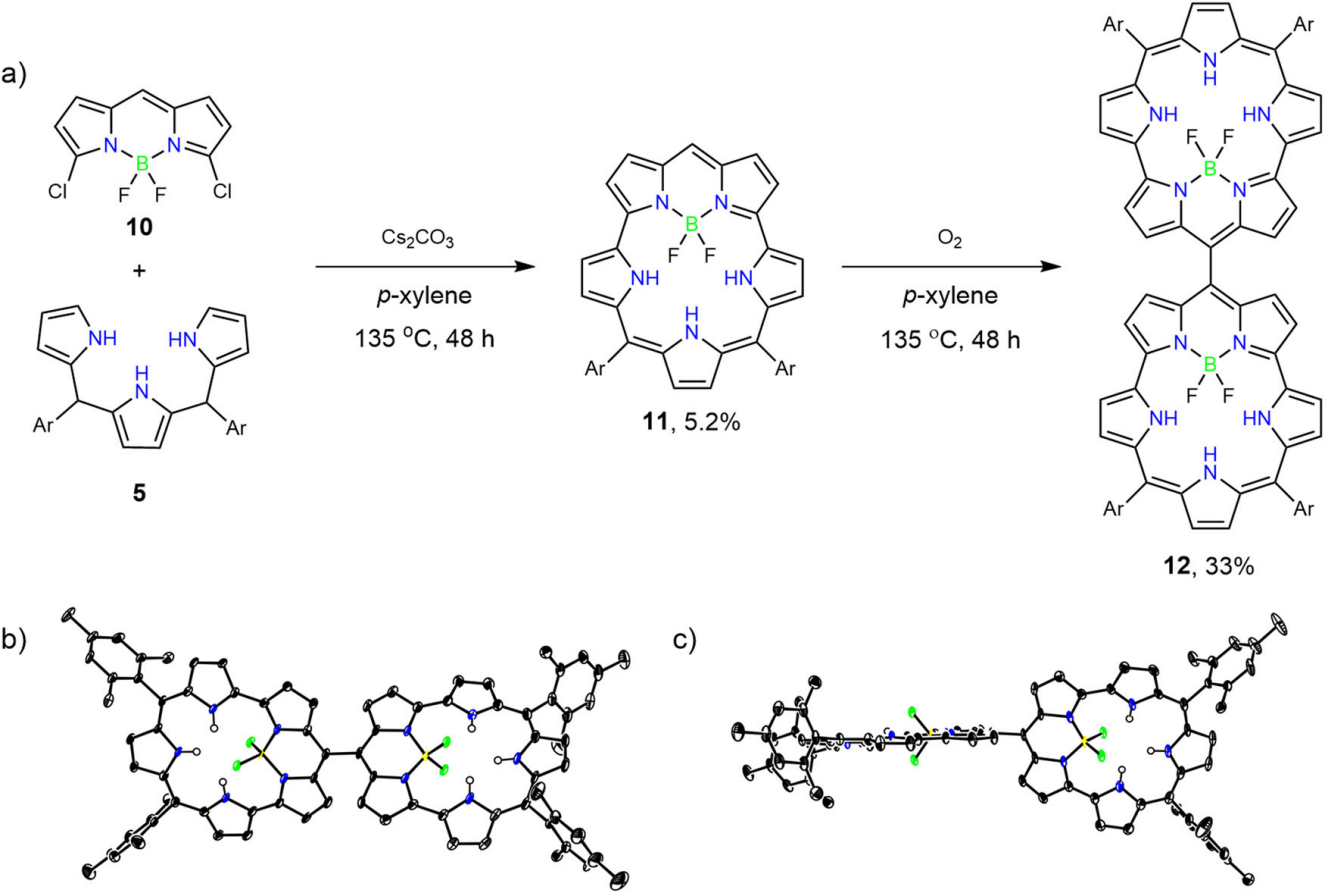
Synthesis and X-ray crystal structures of 12. **a** Synthesis of **12** through **11**. **b** Top view of crystal structure of **12**, and **c** side view of crystal structure of **12**. Solvent molecules and hydrogen atoms on carbon have been omitted for clarity.

With **10** in hand, we tried to synthesize *meso*-diaryl-substituted BF_2_–smaragdyrins by our method^6^. Namely, the reaction of **10** with 5,10-dimesityl-tripyrromethene **5** in the presence of cesium carbonate in refluxing *p*-xylene overnight afforded 5,10-dimesityl-[22]smaragdyrin BF_2_-complex **11** in 5.2% yield after usual work up (Fig. 5). The ^1^H NMR spectrum of **11** in CDCl_3_ displays a singlet due to the *meso*-proton at *δ* = 10.71 and five signals due to the outer pyrrolic *β*-protons between *δ* = 10.46 and 8.94 ppm, a singlet due to the inner NH proton at −7.76 ppm, and a triplet due to the inner NH proton at −4.90 ppm that is coupled with the two fluorine atoms through-space coupling with *J* = 11.2 Hz. The ^1^H NMR spectrum of **11** is similar to that of the triaryl-substituted BF_2_–smaragdyrin reported before^6^, indicating its aromatic character arising from its 22π-circuit. The meso-free smaragdyrin BF2-complex **11** was isolated in a pure form.

In the meanwhile, however, we found that *meso*-free BF_2_-smaragdyrin complex **11** was not stable and slowly oxidized to *meso*–*meso*-linked BF_2_-smaragdyrin dimer **12** in the air. Actually, this oxidative coupling was accelerated at high temperature. After some experimentations, we found that the best yield of **12** (34%) was reproducibly accomplished by heating a *p*-xylene solution of **11** at 135 °C in the air for 48 h. The ^1^H NMR spectrum of **12** in CDCl_3_ is simple, displaying five signals due to the outer pyrrolic protons between 10.44 and 9.06 ppm, a singlet due to the inner NH proton at −7.29 ppm, and a triplet due to the inner NH proton at −4.37 ppm. The disappearance of the *meso*-proton signal of **11** was consistent with the formation of the *meso*-*meso*-linked dimer. The parent ion peak of **12** was observed at *m/z* = 1292.5397 (calcd for [C_82_H_70_B_2_F_4_N_10_]^+^; 1292.5925 ([M]^+^)) in its high-resolution MALDI-TOF mass spectrum. The structure of **12** was unambiguously confirmed by X-ray diffraction analysis (Fig. 5). The C(*meso*)–C(*meso*) bond length is 1.497(4) Å, which is within the range of typical C–C bond. The dihedral angle of the BF_2_–smaragdyrin rings is 62.5(1)°. These structural features indicate that conjugative interaction between the two BF_2_–smaragdyrin units is weak.

### UV/Vis/NIR absorption and fluorescence spectra

**Fig. 6:**
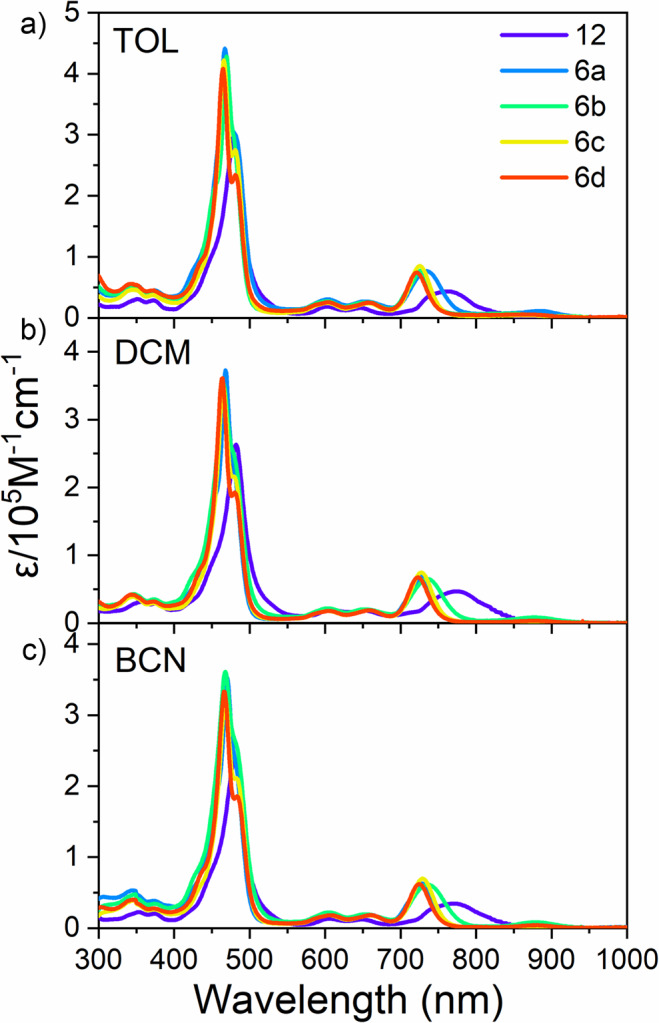
Normalized steady-state absorption spectra. **12** (purple), **6a** (blue), **6b** (green), **6c** (yellow), and **6d** (red) in toluene (**a**), in dichloromethane (**b**), in benzonitrile (**c**).

The UV/Vis/NIR spectra of these BF_2_-smaragdyrins in toluene, CH_2_Cl_2_, and benzonitrile are shown in Fig. 6 and the Supplementary Fig. 21–26). The absorption spectrum of **11** in toluene shows weak Q-bands at 596, 649, and 702 nm, which are similar to that of 5,10,19-triaryl-substituted BF_2_-smaragdyrin. As compared with the absorption spectra of monomer **11**, those of dimers **6a–6d** are all broader both in the Soret and Q-like band regions, probably owing to exciton coupling between the two BF_2_–amaragdyrin units. Consistent with this, the most red-shifted Q-like bands are red-shifted in the order of **6d** < **6c** < **6a** < **6b** < **12** with decreasing distance of the two BF_2_–amaragdyrin units. Namely, *meso–meso*-linked dimer **12** exhibits the most red-shifted and broadest Q-like band at 762 nm. The UV/Vis/NIR absorption spectra of these BF_2_–smaragdyrins are rather insensitive to solvent polarity.

**Fig. 7:**
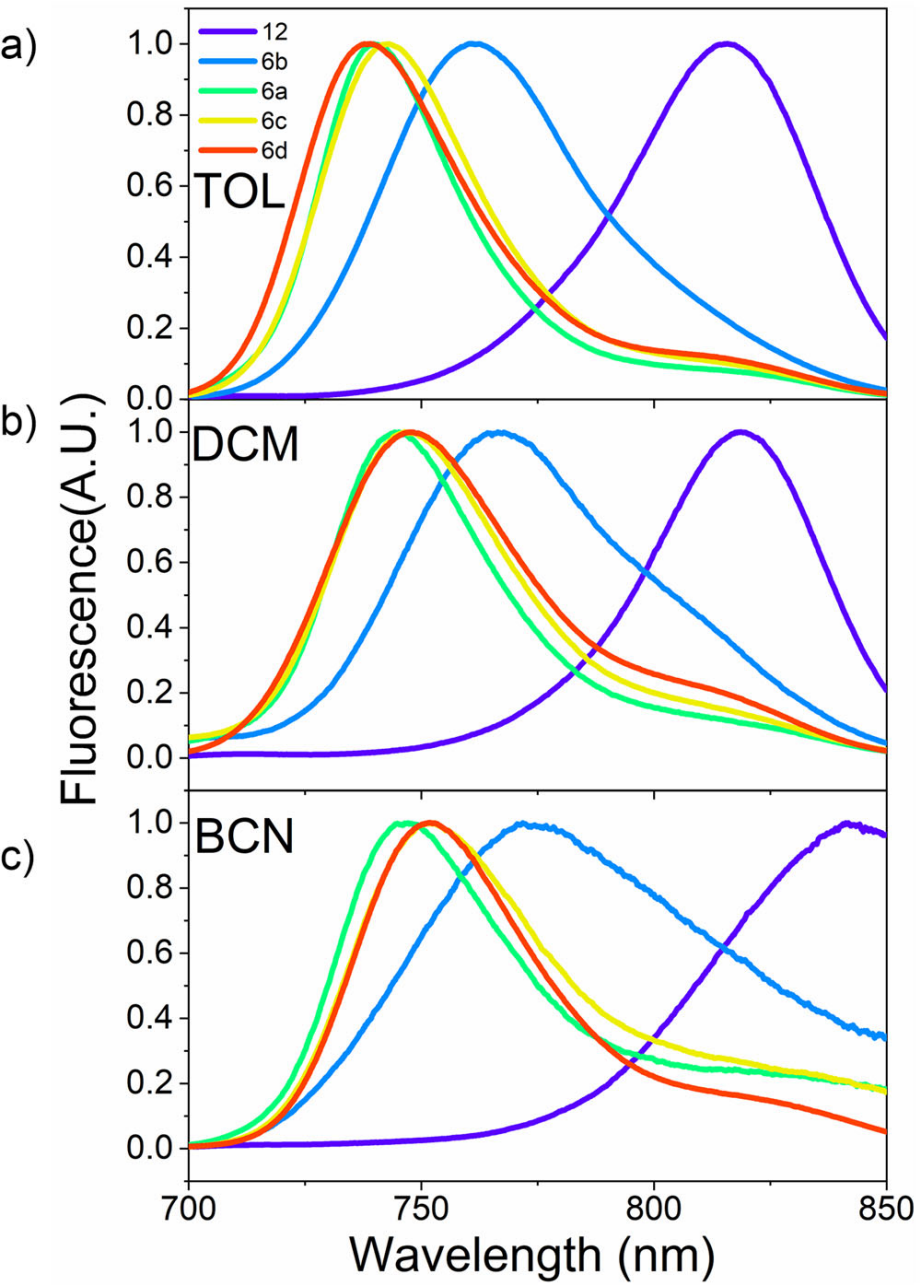
Normalized steady-state fluorescence spectra of BF_2_–smaragdyrins. **12** (purple), **6a** (green), **6b** (blue), **6c** (yellow), and **6d** (red) in toluene (**a**), in dichloromethane (**b**), and in benzonitrile (**c**).

The normalized fluorescence spectra of these BF_2_–smaragdyrin dimers are shown in Fig. 7 and Supplementary Figs. 21–26. Their maximum emission wavelengths are all in the near-infrared region. Gradual redshifts are observed in the fluorescence spectra in going from **11**, **6d**, **6c**, and **6a**, and a substantial shift are found for **6b**, and a bigger shift is observed for **12**. The fluorescence quantum yields of the dimers are quite characteristic, decreasing with a decrease in the distance between the BF_2_–smaragdyrin (Table 1), suggesting that the electronic interaction between the two BF_2_–smaragdyrins causes additional decaying channel. The fluorescence quantum yields in toluene decrease in the order of **11** (0.138) > **6c** (0.079) > **6d** (0.054) > **6b** (0.032) > **6a** (0.028) > **12** (<0.001). The observed practically nonfluorescent behavior of **12** in nonpolar toluene is noteworthy. It is also important that an increase in solvent polarity decreases the fluorescence quantum yield. For example, in the case of **6a**, 0.032 (toluene) > 0.019 (CHCl_3_) > 0.018 (THF) > 0.005 (CH_2_Cl_2_) > < 0.001 (acetonitrile). Similar trends were observed for **6b**–**6d**. These data have suggested that BF_2_–smaragdyrin dimers, despite the identical chromophores in the molecule, undergo intramolecular charge transfer (CT), depending on the solvent polarity and the distance between the two BF_2_–smaragdyrin units. It is probable that the required asymmetric situation is provided by a fluctuation of polar solvents^17–25^. Stokes shifts became increasingly larger from toluene, dichloromethane, and benzonitrile, suggesting that the relaxations of Franck–Condon states to emitting states involve CT. In particular, the photoexcited-state dynamics of **12** is quite curious, since it scarcely emits fluorescence and its Stokes shifts are large, being 868 cm^−1^ in toluene, 811 cm^−1^ in CH_2_Cl_2_ and 1094 cm^−1^ in benzonitrile. These large Stokes shifts suggested that emitting state might have large CT properties. It is notable that dimer **6b** showed relatively large Stokes shifts of 615 cm^−1^ in benzonitrile. It was thought that the extent of the CT in the emitting states depended on the electronic interactions between the two smaragdyrins. Thus, the emitting state of dimer **12** might have the largest CT character in the series.

**Table 1:** Photophysical properties of BF_2_–smaragdyrins in different solvents.

	Solvent	*λ*_Soret_ (nm)	*λ*_Q-max_ (nm)	*λ*_em_(nm)	*E* (eV)^a^	*Φ*_f_^b^	Stokes shift (cm^−1^)	*k*_r_(s^−1^)^d^	*k*_nr_(s^−1^)^d^
**6a**	Toluene	467	728	748	1.68	0.032	367	1.31 × 10^8^	3.97 × 10^9^
	THF	466	730	750	1.68	0.018	365	–	–
	Chloroform	468	731	752	1.67	0.019	382	–	–
	DCM	467	730	750	1.68	0.005	365	1.47 × 10^8^	2.93 × 10^10^
	BCN	470	729	747		0.003	331	1.58 × 10^8^	5.25 × 10^10^
	MeCN	463	727	746	1.68	<0.001	350	–	–
**6b**	Toluene	471	738	752	1.67	0.028	252	1.49 × 10^8^	5.17 × 10^9^
	THF	470	742	758	1.65	0.025	284	–	–
	Chloroform	471	742	757	1.66	0.015	267	–	–
	DCM	468	739	757	1.66	0.005	322	1.85 × 10^8^	3.69 × 10^10^
	BCN	468	737	772		0.002	615	1.33 × 10^8^	6.65 × 10^10^
	MeCN	464	737	750	1.67	<0.001	235	–	–
**6c**	Toluene	466	726	740	1.69	0.079	261	3.50 × 10^8^	4.08 × 10^9^
	THF	465	730	742	1.69	0.043	222	–	–
	Chloroform	467	730	744	1.68	0.047	258	–	–
	DCM	466	729	743	1.69	0.018	258	4.39 × 10^8^	2.40 × 10^10^
	BCN	465	729	753		0.010	437	2.70 × 10^8^	2.68 × 10^10^
	MeCN	462	726	737	1.70	0.013	206	–	–
**6d**	Toluene	464	721	738	1.70	0.054	319	2.30 × 10^8^	4.03 × 10^9^
	THF	463	723	739	1.70	0.041	299	–	–
	Chloroform	464	723	739	1.70	0.037	299	–	–
	DCM	463	723	740	1.70	0.028	318	1.88 × 10^8^	6.53 × 10^9^
	BCN	466	725	751		0.017	478	1.73 × 10^8^	1.00 × 10^10^
	MeCN	459	720	737	1.70	0.017	320	–	–
**11**	Toluene	454, 476	702	715	1.75	0.138	259	–	–
	THF	452, 475	702	718	1.75	0.150	317	–	–
	Chloroform	454, 476	704	717	1.75	0.109	258	–	–
	DCM	453, 475	702	717	1.75	0.117	298	–	–
	MeCN	448, 471	700	714	1.75	0.192	280	–	–
**12**	Toluene	480	762	816	1.57	<0.001	868	–	–
	THF	478	766	–^c^	–	–^c^	–	–	–
	Chloroform	479	770	–^c^	–	–^c^	–	–	–
	DCM	478	768	819	–	<0.001	811	–	–
	BCN	480	771	842		<0.001	1094	–	–

^a^Excitation energy of S_1_-state determined by the midpoint of the absorption spectrum and the fluorescence spectrum.

^b^Fluorescence quantum yield.

^c^Fluorescence quantum yield is too low to be accurately determined.

^d^The rates of radiative and non-radiative decay.

We measured the fluorescence lifetime of BF_2_–smaragdyrin dimers using the time-correlated single-photon counting (TCSPC) method and the results are summarized in Supplementary Note 1, Supplementary Figs. S57 and S58. We representatively selected three solvents, toluene (*ε* = 2.38, TOL), CH_2_Cl_2_ (*ε* = 8.93, DCM), and benzonitrile (*ε* = 25.93, BCN). Compared with the fluorescence lifetime of BF_2_–smaragdyrin dimers in toluene, those in CH_2_Cl_2_ and benzonitrile are clearly shortened, showing the favorable formation of CT state in polar solvents. In the cases of **6a–6d**, the fluorescence lifetimes in polar solvents become shorter in **6a** and **6b** compared with that of **6d**, intermediate in **6c**. These results indicate stronger CT characters in spatially closer dimers with stronger electronic interactions. The fluorescence lifetimes in benzonitrile are 0.98 ns for **6d** > 0.37 ns for **6c** > 0.19 ns for **6a** > 0.15 ns for **6b**, while that of **12** is 0.37 ns, being longer than those of **6a**–**6c**, suggesting that the emitting state of **12** is considerably different from those of **6a–d**.

**Fig. 8:**
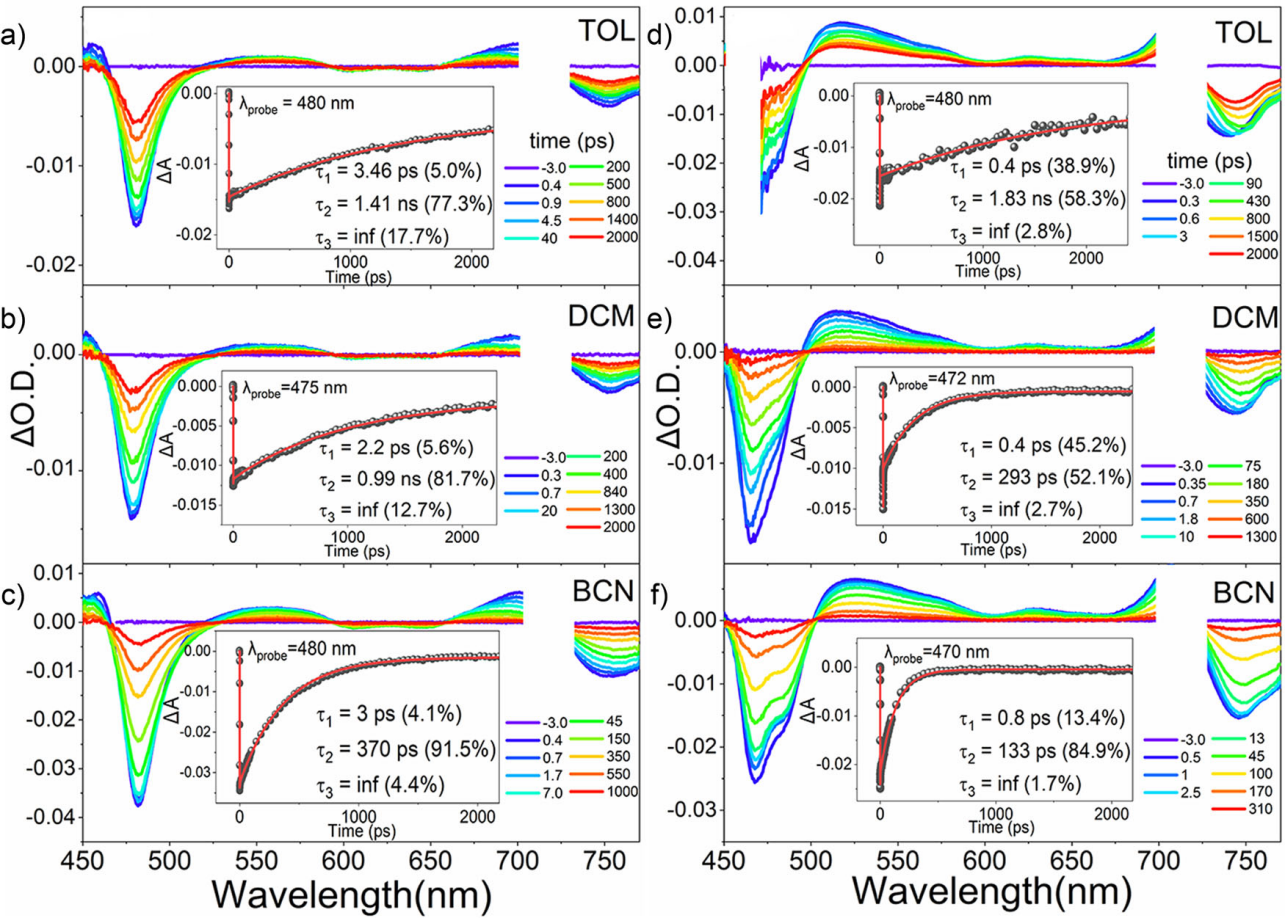
Femtosecond-transient absorption (fs-TA) spectra and decay profiles of BF_2_-Smaragdyrins. **12**, **6b** in toluene (**a**, **d**), in dichloromethane (**b**, **e**), in benzonitrile (**c**, **f**). The samples were pumped at 710 nm with probing visible region to the near-IR region (450–780 nm).

Furthermore, to explore the excited-state population dynamics, femtosecond transient absorption (fs-TA) measurements were carried out in the region of 450–780 nm using an excitation pulse at 710 nm (Fig. 8, Supplementary Note 2, Supplementary Figs. 59–61). The TA spectra of the BF_2_–smaragdyrin dimers display ground-state bleaching (GSB) features in 450–500 nm and above 725 nm regions, which correspond to the steady-state absorption spectra, and broad excited-state absorption (ESA) features in 500–700 nm region.

The TA spectral signatures of the BF_2_–smaragdyrin dimers are similar to those reported by the previous studies on the monomer^6^. No significant spectral changes in the TA spectra were observed except for the decay time constants. Namely, we did not detect charge-separated states. The TA kinetic profiles are well-fitted with triple exponential decay functions. We attributed the initial decay component (*τ*_1_) to the conformational dynamics. In polar solvents, contribution by the *τ*_1_ component increases in the order of **12** < **6a** < **6b** < **6c** which is in accordance with the increased conformational flexibility of the smaragdyrin dimers. Based on the solvation time constants of CH_2_Cl_2_ and benzonitrile, we assigned the structural change as the symmetry-breaking charge transfer process. Especially, in **12**, this process is favorable in benzonitrile, which is consistent with the steady-state fluorescence spectra.

Additionally, we found that the *τ*_2_ component corresponded to the fluorescence lifetimes measured by TCSPC. Thus, we attributed the *τ*_2_ component to the singlet state fluorescence or CT emission decay process, depending on the electronic structures of dimers. These time constants of a series of BF_2_–smaragdyrin dimers become gradually shortened with an increase of solvent polarity. In the cases of **6a** and **6b**, which have one phenylene bridge, *meta*- and *para*-phenylene linker, respectively, while the spatial separation between the monomer units of **6a** is shorter than **6b**, **6a** exhibits a longer lifetime because the electronic interaction is weaker than **6b**. The major decaying components (*τ*_2_) of **12** were 1.4 ns in toluene, 0.98 ns in CH_2_Cl_2_, and 0.37 ns in benzonitrile. These decaying time constants cannot explain the observed very low fluorescent quantum yields of **12**. This means that the emitting state of **12** is not a usual S_1_-excited state but probably a considerable CT state with a small radiative decaying channel to the ground state. Most probably, the CT state of **12** is formed at a very early time assisted by asymmetric solvation of polar solvent, and decays with a slow time constant to regenerate the ground state. While not a symmetric dimer, Similar accelerated decay of the excited state via charge transfer interaction was reported for a directly linked Zn(II) porphyrin–Zn(II) pheophorbide dyad^28^.

Finally, we attributed the τ_3_ component to the long-lived triplet state formation through the intersystem crossing. Consequently, the TA spectral features and excited state dynamics unveil that the charge transfer processes, corroborated with the structural evolution according to the structural flexibility and the distance between the monomer units, occur in the BF_2_–smaragdyrin dimers.

### Electrochemical properties

Estimation of the free energy gap for the symmetry-breaking charge separation is important to understand the photo-excited dynamics of the smaragdyrin dimers. Thus, we examined their electrochemical properties. The electrochemical properties of **6a**–**6d**, and **12** are examined by cyclic voltammetry and differential pulse voltammetry in CH_2_Cl_2_ and benzonitrile containing 0.1 M n-Bu_4_NPF_6_ as an electrolyte and the results are summarized in Table 2. (For detailed electrochemical data see Supplementary Fig. 27–37, Supplementary Tables 1 and 2) In both solvents, the energy levels of the S1 states and the charge-separated states are almost the same. Considering that the reorganization would be necessary for the charge separation, the symmetry-breaking full charge separation would be difficult. Instead, these data would support the feasibility of partial CT.

**Table 2:** Summary of redox potentials^a^.

	*E*_ox.1_/V	*E*_red.1_/V	△*E*_HL_/eV^d^	*E*_(S1)_/V	△*E*_HL_ − *E*_(S1)_/eV
**6a**^b^	0.02	−1.66	1.68	1.67	0.01
**6b**^b^	0.00	−1.65	1.65	1.65	0.00
**6c**^b^	0.00	−1.66	1.66	1.68	−0.02
**6d**^b^	−0.02	−1.66	1.64	1.70	−0.04
**12**^b^	−0.06	−1.62	1.56	1.56	0.00
**6a**^c^	0.05	−1.61	1.66	1.66	0
**6b**^c^	0.05	−1.60	1.65	1.65	0
**6c**^c^	0.02	−1.60	1.62	1.65	−0.03
**6d**^c^	0.02	−1.59	1.61	1.61	0
**12**^c^	−0.02	−1.53	1.51	1.51	0

^a^Potentials [V] vs. ferrocene/ferrocenium ion. Scan rate, 0.05 Vs^−1^; working electrode, glassy carbon; counter electrode, Pt wire; supporting electrolyte, 0.1 M nBu_4_NPF_6_ in CH_2_Cl_2_; reference electrode, Ag/AgNO_3_.

^b^Measured in CH_2_Cl_2_.

^c^Measured in benzonitrile.

^d^Electrochemical HOMO-LUMO gaps (*ΔE*_HL_ = *e*(*E*_ox.1_ – *E*_red.1_) [eV]).

### Theoretical calculations

All calculations were carried out using the Gaussian 09 program. All structures were fully optimized without any symmetry restriction. All geometries were optimized with the crystal structures as the starting structure at the density functional theory (DFT) method with restricted B3LYP (Becke’s three-parameter hybrid exchange functionals and the Lee–Yang–Parr correlation functional) level^19–25^. We calculated the absorption spectra of important compounds, **12**, **6b**, and **6c** (For detailed theoretical calculations data see Supplementary Fig. 54–56, Supplementary Data 9). The calculation results of time-dependent density functional theory (TD-DFT) are in good agreement with the actual absorption spectral data of the compound. The maximum absorption peaks of the Q-like band of these compounds mainly come from electron transition from the highest occupied molecular orbital (HOMO) to the lowest unoccupied molecular orbital (LUMO).

### Summary

In summary, we have synthesized *meso*-diaryl-substituted BF_2_–smaragdyrin dimers in low but acceptable yields via the twofold S_N_Ar reaction and subsequent oxidation reaction with DDQ. We also synthesized *meso*-free and *meso–meso* directly linked BF_2_–smaragdyrin dimer. These molecules were investigated by UV/Vis/NIR absorption, fluorescence measurements, TCSPC method, fs-TA measurements, cyclic voltammetry and theoretical quantum calculations. The excited-state decays of the BF_2_–smaragdyrin dimers were accelerated with increase of the solvent polarity and a decrease in the distance between the smaragdyrin units. It has been concluded that symmetry-breaking charge transfer plays an important role in the excited-state decays of the smaragdyrin dimers in polar solvents.

## Methods

### Materials and characterization

^1^H NMR (500 MHz) and ^13^C NMR (126 MHz) spectra were taken on a Bruker ADVANCE-500 spectrometer, and chemical shifts were reported as the delta scale in ppm relative to CHCl_3_ as an internal reference for ^1^H NMR (*δ* = 7.260 ppm) and ^13^C NMR (*δ* = 77.000 ppm) (For ^1^H NMR and ^13^C NMR spectra see Supplementary Figs. 1–20). UV/Vis absorption spectra were recorded on a Shimadzu UV-3600 spectrometer. Fluorescence emission spectra were recorded on a HITACHI F-4500 spectrometer (For UV/Vis and fluorescence emission spectra see Supplementary Fig. 21–26). MALDI-TOF mass spectra were obtained with a Bruker Ultrafle Xtreme MALDI-TOF/TOF Mass spectrometer and Thermo Scientific Q Exactive ESI Mass spectrometer (For HRMS spectra see Supplementary Figs. 42–53). X-ray crystallographic data were taken on an Agilent SuperNova X-ray diffractometer equipped with a large-area CCD detector. Using Olex2, structures of compounds **6b**, **6c**, **10**, and **12** were solved with the ShelXS structure solution program using Direct Methods and refined with the ShelXL refinement package using least squares minimization. Disordered solvent molecules were treated by the SQUEEZE program of Platon (For single crystal data see Supplementary Figs. 38–41, Supplementary Tables 3–6). Redox potentials were measured by the cyclic voltammetry and differential pulse voltammetry method on an ALS660 electrochemical analyzed model (Solvent: PhCN or CH_2_Cl_2_, electrolyte: 0.1 M n-Bu_4_NPF_6_, working electrode: glassy carbon, reference electrode: Ag/AgNO_3_, Counter electrode: Pt wire, scan rate: 0.05 V/s, external reference: ferrocene/ferrocenium cation). Benzonitrile passed through the alumina column was used for electrochemical analysis. Unless otherwise noted, materials obtained from commercial suppliers were used without further purification.

### Synthesis of 4a–4d

To a solution of corresponding dipyrromethane (1 mmol) in THF (70 mL) at −78 °C, NBS (712 mg, 4 mmol) was added in three portions within 1 h. The mixture was stirred at a low temperature for 3 h. Then DDQ (499 mg, 2.2 mmol) was added to the solution. The mixture was stirred at −78 °C, for 0.5 h and then at room temperature for 3 h. The residue was purified through a long Al_2_O_3_ column using THF as an eluent. The solvent was removed in vacuo. Then, CH_2_Cl_2_ (100 mL) was added to the remaining solids. TEA (4.0 mL, 28 mmol) and BF_3_•OEt_2_ (5.0 mL, 45 mmol) were added dropwise to the solution. The mixture was stirred at room temperature for 24 h. The solvent was removed in vacuo. Products were simply purified by recrystallization with MeOH/H_2_O. **4a** (280 mg), **4b** (274 mg), **4c** (340 mg) and **4d** (452 mg). **4a**: HR-MS (MALDI-TOF-MS): *m/z* = 773.7518, calcd for (C_24_H_12_B_2_Br_4_F_4_N_4_)^+^ = 773.7883 ([M]^+^). **4b**: HR-MS (MALDI-TOF-MS): *m/z* = 773.7731, calcd for (C_24_H_12_B_2_Br_4_F_4_N_4_)^+^ = 773.7883 ([M]^+^). **4c**: HR-MS (MALDI-TOF-MS): *m/z* = 849.7946, calcd for (C_30_H_16_B_2_Br_4_F_4_N_4_)^+^ = 849.8198 ([M]^+^). **4d**: HR-MS (MALDI-TOF-MS): *m/z* = 925.8197, calcd for (C_36_H_20_B_2_Br_4_F_4_N_4_)^+^ = 925.8514 ([M]^+^).

### Synthesis of 6a–6d

A solution of brominated BODIPY compound (0.051 mmol), **5** (50 mg, 0.108 mmol), and Cs_2_CO_3_ (180 mg, 0.60 mmol) were purged with argon and then charged with dry *o*-DCB (16 mL). The mixture was stirred at 135 °C for 48 h. The reaction mixture was passed through a short silica gel column using CHCl_3_ as an eluent and the solvent was evaporated in vacuo. The product was purified by column chromatography on silica gel (CH_2_Cl_2_/hexanes, 1:2 V/V) and recrystallization with CH_2_Cl_2_/MeOH. **6a** (1.9 mg, 0.0014 mmol, 2.8% yield), **6b** (1.5 mg, 0.0011 mmol, 2.2% yield), **6c** (2.0 mg, 0.0014 mmol, 2.7% yield), and **6d** (1.9 mg, 0.0012 mmol, 2.4% yield) were obtained all as green solids. **6a**: ^1^H NMR (500 MHz, CDCl_3_): *δ* = 10.49 (d, 4H, *J* = 4.5 Hz, *β*-H), 10.13–10.11 (m, 8H, *β*-H), 9.95 (s, 1H, Ar-H), 9.17 (dd, 2H, *J* = 7.5, 1.5 Hz, Ar-H), 9.08 (d, 4H, *J* = 1.0 Hz, *β*-H), 8.88–8.87 (m, 4H, *β*-H), 8.59 (t, 1H, *J* = 7.5 Hz, *β*-H), 7.42 (s, 8H, Ar-H), 2.72 (s, 12H, Me-H), 1.92 (s, 24H, Me-H), −4.44 (t, 4H, *J* = 11.5 Hz, N-H), and −7.37 (s, 2H, N-H) ppm. ^13^C NMR (126 MHz, CDCl_3_) *δ* = 140.1, 138.4, 138.0, 134.9, 134.4, 132.4, 131.4, 129.8, 128.3, 125.7, 124.6, 123.7, 122.0, 119.6, 118.3, 116.6, 105.8, 21.6, and 21.2 ppm. *λ*_max_ (*ε* [M^−1^cm^−1^] in toluene) =348 (74000), 373 (60000), 467(441000), 604(38000), 658(37000), 728(129000) nm. HR-MS (MALDI-TOF-MS): *m/z* = 1368.5771, calcd for (C_88_H_74_B_2_F_4_N_10_)^+^ = 1368.6239 ([M]^+^). **6b**: ^1^H NMR (500 MHz, CDCl_3_): *δ*= 10.59 (d, 4H, *J* = 4.5 Hz, *β*-H), 10.21–10.20 (m, 4H, *β*-H), 10.17 (d, 4H, *J* = 4.0 Hz, *β*-H), 9.30 (s, 4H, Ar-H), 9.13 (s, 4H, *β*-H), 8.95 (br, 4H, *β*-H), 7.47 (s, 8H, Ar-H), 2.75 (s, 12H, Me-H), 1.98 (s, 24H, Me-H), −4.34 (t, *J* = 10.5 Hz, 4H, N-H), and −7.27 (s, 2H, N-H) ppm. ^13^C NMR (126 MHz, CDCl_3_) *δ* = 140.1, 139.2, 138.4, 138.0, 135.0, 134.2, 132.4, 131.4, 129.8, 128.3, 125.7, 124.7, 123.8, 122.0, 119.7, 118.4, 100.0, 21.6, and 21.2 ppm. *λ*_max_ (*ε* [M^−1^cm^−1^] in toluene) = 347 (58000), 371 (47000), 471 (428000), 600(32000), 647(28000), 738(105000) nm. HR-MS (MALDI-TOF-MS): m/z = 1368.5771, calcd for (C_88_H_74_B_2_F_4_N_10_)^+^ = 1368.6239 ([M]^+^). **6c**: ^1^H NMR (500 MHz, CDCl_3_): *δ* = 10.48 (d, 4H, *J* = 4.0 Hz, *β*-H), 10.14–10.13 (m, 4H, *β*-H), 9.88 (d, 4H, *J* = 4.5 Hz, *β*-H), 9.09 (s, 4H, *β*-H), 8.95 (d, 4H, *J* = 7.5 Hz, Ar-H), 8.90 (dd, 4H, *J* = 4.0, 1.5 Hz, *β*-H), 8.64 (d, 4H, *J* = 7.5 Hz, Ar-H), 7.45 (s, 8H, Ar-H), 2.74 (s, 12H, Me-H), 1.95 (s, 24H, Me-H), −4.39 (t, *J* = 11.5 Hz, 4H, N-H), and −7.31 (s, 2H, N-H) ppm. ^13^C NMR (126 MHz, CDCl_3_) *δ* = 140.1, 138.4, 138.0, 135.7, 134.9, 132.2, 131.4, 129.8, 128.3, 127.4, 125.7, 124.6, 123.6, 121.9, 119.5, 118.3, 116.6, 105.8, 21.6, and 21.2 ppm. *λ*_max_ (*ε* [M^−1^cm^−1^] in toluene) = 346 (45000), 371 (36000), 466 (422000), 604(24000), 657(23000), 726(80000) nm. HR-MS (MALDI-TOF-MS): m/z = 1444.6035, calcd for (C_94_H_78_B_2_F_4_N_10_)^+^ = 1444.6554 ([M]^+^). **6d**: ^1^H NMR (500 MHz, CDCl_3_): *δ* = 10.44 (d, 4H, *J* = 4.4 Hz, *β*-H), 10.12–10.11 (m, 4H, *β*-H), 9.80 (d, 4H, *J* = 4.0 Hz, *β*-H), 9.08 (s, 4H, *β*-H), 8.88 (dd, 4H, *J* = 4.0, 1.5 Hz, *β*-H), 8.84 (d, 4H, *J* = 8.0 Hz, Ar-H), 8.42 (d, 4H, *J* = 7.5 Hz, Ar-H), 8.32 (s, 4H, Ar-H), 7.44 (s, 8H, Ar-H), 2.73 (s, 12H, Me-H), 1.94 (s, 24H, Me-H), −4.40 (t, *J* = 11.5 Hz, 4H, N-H), and −7.32 (s, 2H, N-H) ppm. ^13^C NMR (126 MHz, CDCl_3_) *δ* = 140.1, 138.4, 138.0, 135.5, 134.9, 132.1, 131.4, 129.8, 128.3, 128.1, 127.0, 125.7, 124.6, 123.6, 121.8, 119.5, 118.2, 116.61, 116.58, 105.7, 21.6, and 21.2 ppm. *λ*_max_ (*ε* [M^−1^cm^−1^] in toluene) = 344 (47000), 371 (34000), 464 (408000), 603(20000), 657(20000), 721(74000) nm. MS (MALDI-TOF-MS): *m/z* = 1520.6715, calcd for (C_100_H_82_B_2_F_4_N_10_)^+^ = 1520.6868 ([M]^+^).

### Synthesis of 9

To a slurry of *p*-chloranil (1850 mg, 7.4 mmol) in CH_2_Cl_2_ (140 mL) at −40 °C under nitrogen, a solution of **7** (1000 mg, 6.8 mmol) in CH_2_Cl_2_ (200 mL) under N_2_ was added dropwise over several minutes. The reaction mixture was stirred for 3 h. The color of the mixture changed from brown to bright yellow. After DIPEA (7.0 mL, 41 mmol) was added, the solution was stirred for 30 min. BF_3_•OEt_2_ (6.8 mL, 61.2 mmol) was then added slowly over several min, and the mixture was stirred for 18 h, during which time the temperature was allowed to rise to 22 °C. The fluorescent solution was sonicated for 30 min and then filtered through a Celite pad to remove insoluble materials. Then, the reaction mixture was washed with saturated aq. NH_4_Cl and the organic extract was dried over MgSO_4_. The solvent was removed in vacuo. Purification using silica gel (CH_2_Cl_2_/hexanes, 1:1 V/V) gave **9** as dark red solids (488 mg, 38 %). **9**: ^1^H NMR (500 MHz, CDCl_3_) *δ* = 7.90 (s, 2H, *α*-H), 7.42 (s, 1H, *meso*-H), 7.15 (d, *J* = 4.0 Hz, 2H, *β*-H), and 6.55 (d, *J* = 4.0 Hz, 2H, *β*-H) ppm. MS (ESI-MS): *m/z* = 193.0739, calcd for (C_9_H_8_BF_2_N_2_)^+^ = 193.0743 ([M + H]^+^).

### Synthesis of 10

A mixture of BODIPY **9** (30 mg, 0.156 mmol), CuCl_2_•2H_2_O (132.9 mg, 0.78 mmol), and Cu(OTf)_2_ (282.1 mg, 0.78 mmol) in CH_3_CN (6 mL) was stirred under air at 80 °C for 20 min. After cooling down to room temperature, the reaction mixture was poured in CH_2_Cl_2_, and washed with sat aq. NH_4_Cl four times, dried over anhydrous Na_2_SO_4_, and filtered and evaporated under vacuum. Purification using a silica gel column with an eluent of CH_2_Cl_2_/hexanes, 1:1 V/V and recrystallization with n-hexane gave **10** as red solids (17 mg, 55 %). **10**: ^1^H NMR (500 MHz, CDCl_3_) *δ* = 7.14 (s, 1H, *meso*-H), 7.09 (d, 2H, *J* = 4.0 Hz, *β*-H), and 6.44 (d, 2H, *J* = 4.0 Hz, *β*-H) ppm. ^13^C NMR (126 MHz, CDCl_3_) *δ* = 145.8, 134.1, 131.4, 127.8, and 119.2 ppm. HR-MS (MALDI-TOF-MS): *m/z* = 259.9694, calcd for (C_9_H_5_BCl_2_F_2_N_2_)^+^ = 259.9887 ([M]^+^).

### Synthesis of 11

A solution of **10** (30 mg, 0.108 mmol), **5** (54.1 mg, 0.118 mmol), and Cs_2_CO_3_ (141.3 mg, 0.432 mmol) was purged with argon, and then charged with *p*-xylene (10 mL). The mixture was stirred at reflux for 48 h. The reaction mixture was passed through a short silica-gel column using CHCl_3_ as an eluent and the solvent was evaporated in vacuo. The product was purified by column chromatography on silica gel (CH_2_Cl_2_/hexanes, 1:4 V/V) and recrystallization with CH_2_Cl_2_/MeOH, **11** (3.6 mg, 0.0056 mmol, 5.2% yield) was obtained as green solids. Samples can be stored under inert atmosphere environment at low temperature. **11**: ^1^H NMR (500 MHz, CDCl_3_): *δ* = 10.71 (s, 1H, *meso*-H), 10.46 (d, 2H, *J* = 4.0 Hz, *β*-H), 10.16–10.15(m, 2H, *β*-H), 9.84 (d, 2H, *J* = 4.0 Hz, *β*-H), 9.14 (s, 2H, *β*-H), 8.94 (dd, 2H, *J* = 4.0, 1.5 Hz, *β*-H), 7.44 (s, 4H, Ar-H), 2.73 (s, 6H, Me-H), 1.91 (s, 12H, Me-H), −4.90 (t, 2H, *J* = 11.0 Hz, N-H), and −7.76 (s, 1H, N-H) ppm. ^13^C NMR (126 MHz, CDCl3) *δ* = 140.1, 138.5, 138.0, 134.6, 132.6, 131.1, 129.2, 128.3, 125.5, 124.4, 123.2, 121.8, 119.6, 118.5, 105.5, 100.6, 21.6, and 21.2 ppm; HR-MS (MALDI-TOF-MS): *m/z* = 647.2793, calcd for (C_41_H_36_BF_2_N_5_)^+^ = 647.3033 ([M]^+^).

### Synthesis of 12

A solution of **11** (9 mg, 0.014 mmol) in *p*-xylene (1 mL) was stirred in the air at 135 °C for 48 h. The solvent was evaporated in a vacuo. The product was purified by column chromatography on silica gel (CH_2_Cl_2_/hexanes, 1:2 V/V) and recrystallization with CH_2_Cl_2_/MeOH gave **12** (3.0 mg, 0.0024 mmol, 34% yield) as yellow-green solids. **12**: ^1^H NMR (500 MHz, CDCl_3_): *δ* = 10.41 (d, 4H, *J* = 4.5 Hz, *β*-H), 10.22 (dd, 4H, *J* = 4.5, 1.5 Hz, *β*-H), 9.33 (d, 4H, *J* = 4.5 Hz, *β*-H), 9.23 (s, 4H, *β*-H), 9.02 (dd, 4H, *J* = 4.0, 1.5 Hz, *β*-H), 7.49 (s, 8H, Ar-H), 2.76 (s, 12H, Me-H), 2.02 (s, 24H, Me-H), −4.41 (t, 4H, *J* = 11.5 Hz, N-H), and −7.32 (s, 2H, N-H) ppm. ^13^C NMR (126 MHz, CDCl_3_) *δ* = 140.2, 138.5, 138.1, 136.8, 134.9, 131.5, 129.0, 128.3, 125.7, 125.2, 125.1, 122.2, 120.0, 118.9, 112.1, 106.1, 21.6, and 21.3 ppm. *λ*_max_ (*ε* [M^−1^cm^−1^] in toluene) =351 (36000), 373 (33000), 480 (301000), 604(20000), 649(19000), 762(52000) nm. HR-MS (MALDI-TOF-MS): *m/z* = 1292.5397, calcd for (C_82_H_70_B_2_F_4_N_10_)^+^ = 1292.5925 ([M]^+^).

## Supplementary information

Peer Review File

Supplementary Information

Description of Additional Supplementary Files

Supplementary Data 1

Supplementary Data 2

Supplementary Data 3

Supplementary Data 4

Supplementary Data 5

Supplementary Data 6

Supplementary Data 7

Supplementary Data 8

Supplementary Data 9

## Data Availability

The X-ray crystallographic coordinates for structures reported in this study have been deposited at the Cambridge Crystallographic Data Center (CCDC), under deposition numbers 2183007, 2191271, 2183009, 2183010 (**6b**, **6c**, **10**, and **12**). These data can be obtained free of charge from The Cambridge Crystallographic Data Center via www.ccdc.cam.ac.uk/data_request/cif. The data obtained in this study are available within this article and its Supplementary information and are also from the corresponding authors upon reasonable request. Original ^1^H and ^13^C spectra, UV/vis absorption spectra and fluorescence spectra, X-ray crystal data, electrochemical data, HR-MS Spectra, TD-DFT calculation results, time-resolved fluorescence decay data, femtosecond transient absorption spectra and decay profiles of the compounds obtained in this manuscript are available in Supplementary information, the cif file of **6b** is available in Supplementary Data 1, and check cif file of **6b** is available in Supplementary Data 2, the cif file of **6c** is available in Supplementary Data 3, and check cif file of **6c** is available in Supplementary Data 4, the cif file of **10** is available in Supplementary Data 5, and check cif file of **10** is available in Supplementary Data 6, the cif file of **12** is available in Supplementary Data 7, and check cif file of **12** is available in Supplementary Data 8, optimized atomic coordinates are available in Supplementary Data 9. The authors declare that all other data supporting the findings of this study are available within the paper and its Supplementary information files.
