# Supplementary material for: Aromatic-bridged and meso-meso-linked BF2-smaragdyrin dimers exhibit fast decays in polar solvents by symmetry-breaking charge transfer
Source: Commun Chem. 2023 Feb 9;6:25. doi: 10.1038/s42004-023-00822-8 (PMC9911704; doi:10.1038/s42004-023-00822-8)
Supplement: Supplementary file 2 — Supplementary Information [file 42004_2023_822_MOESM2_ESM.pdf]

Supplementary Information for

Aromatic-bridged and *meso-meso*-linked BF_2-smaragdyrin dimers exhibit fast decays in polar solvents by symmetry-breaking charge transfer

1.NMR Spectra of Compounds

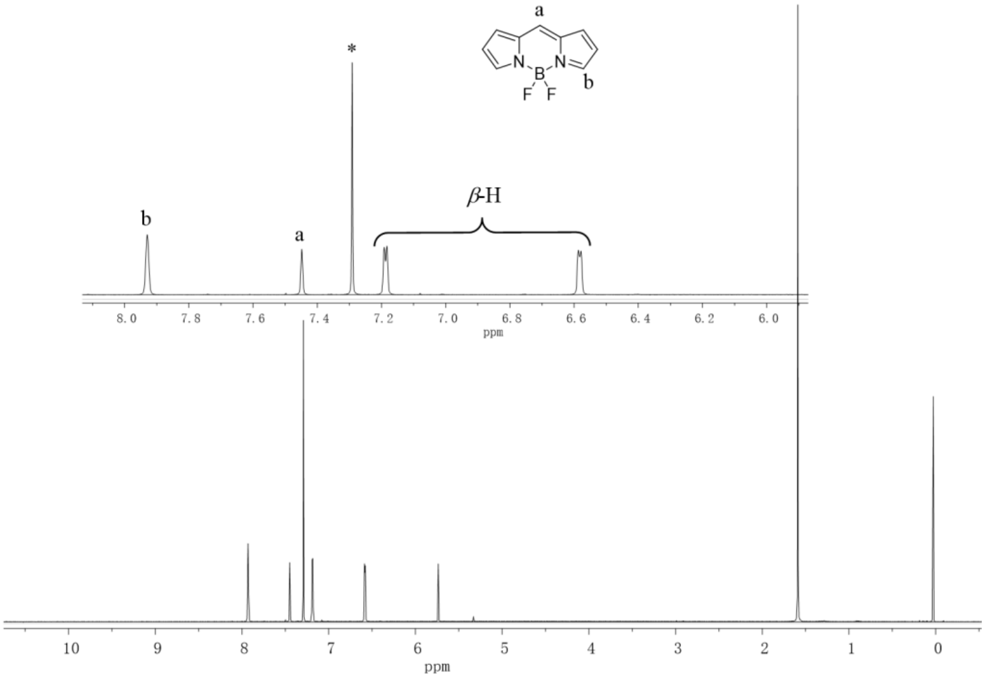

Supplementary Figure 1. ¹H NMR spectrum of **9** in $CDCl_3$. Asterisk means residual solvents or impurity.

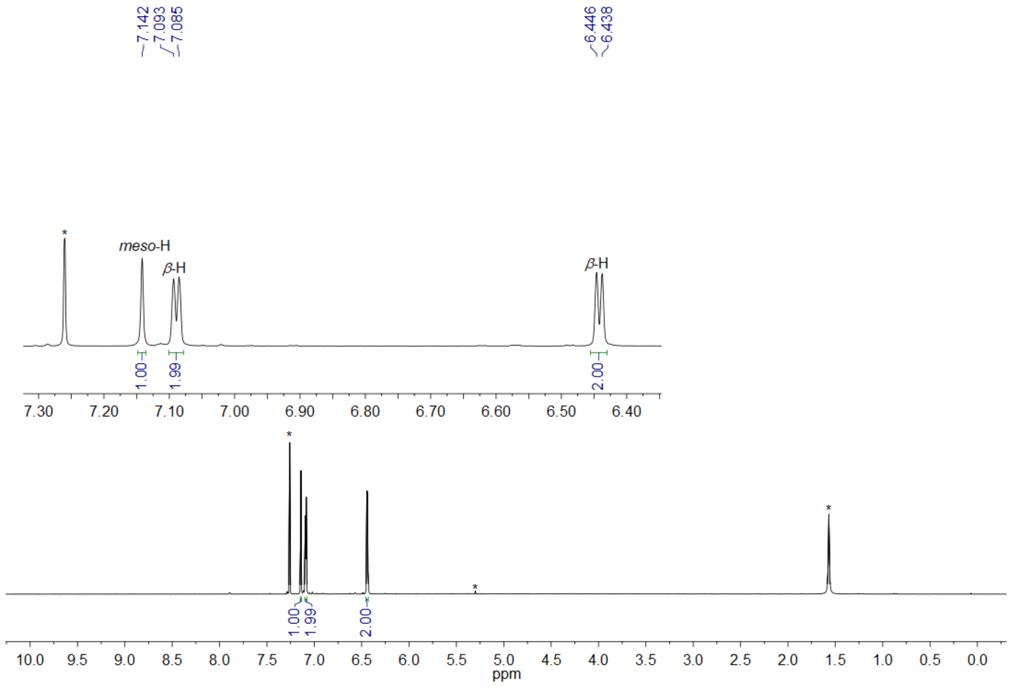

Supplementary Figure 2. ¹H NMR spectrum of **10** in $CDCl_3$. Asterisk means residual solvents or impurity.

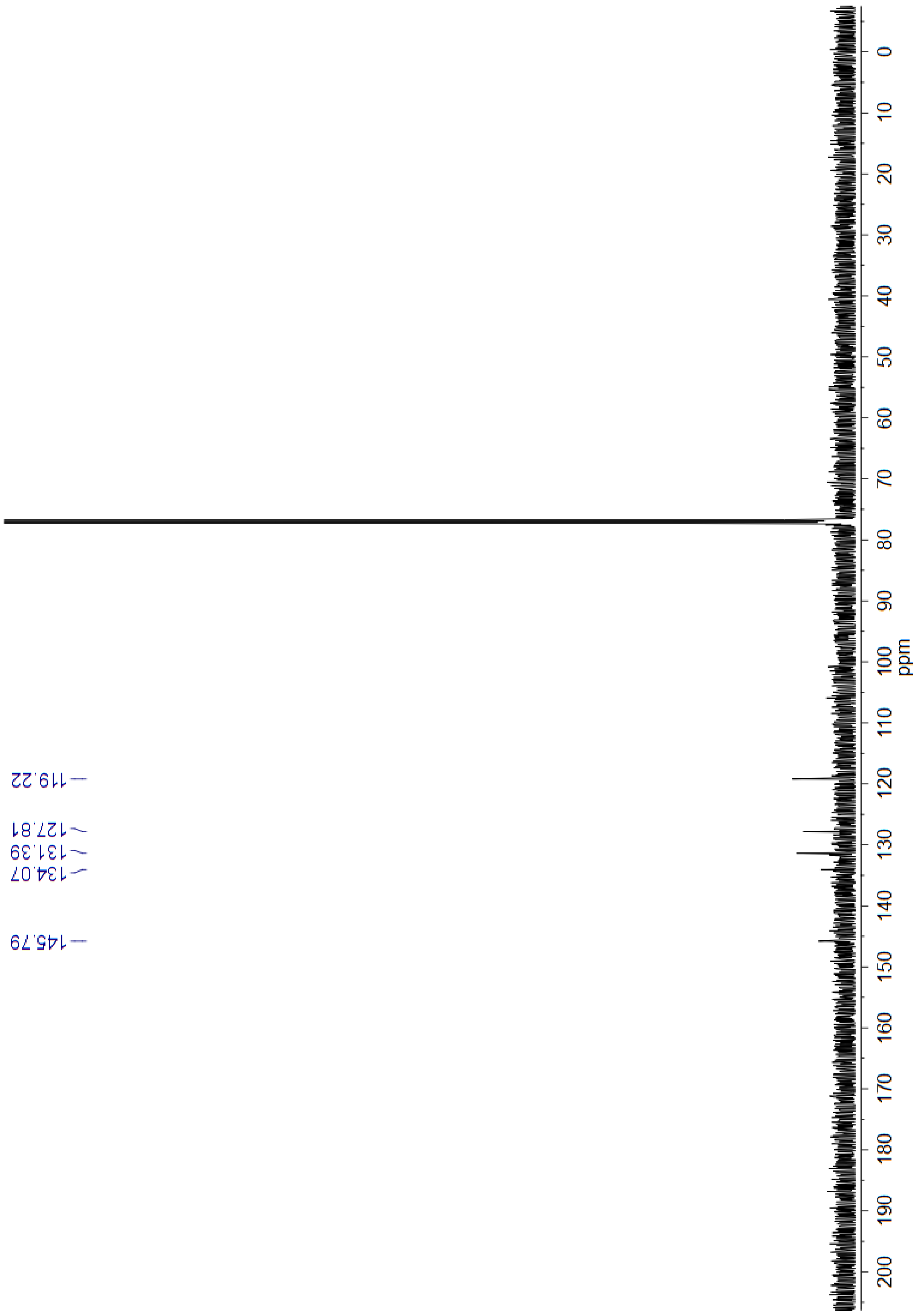

Supplementary Figure 3. ^{13}C NMR spectrum of **10** in $CDCl_3$.

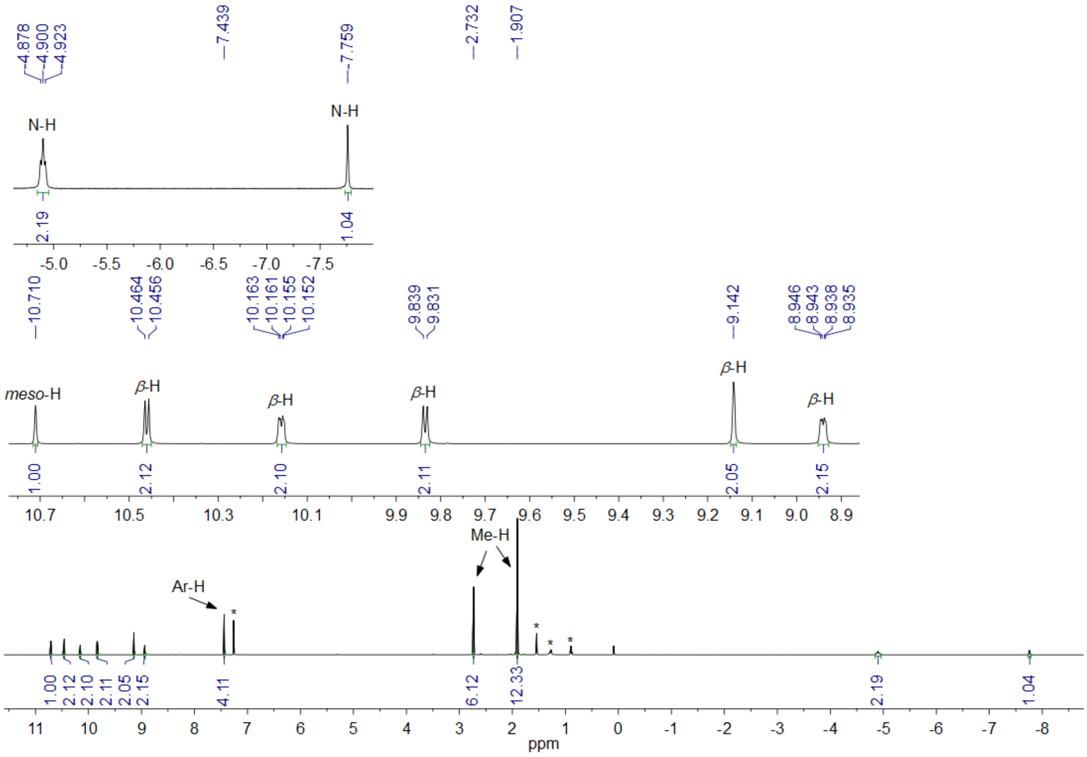

Supplementary Figure 4. ^{1}H NMR spectrum of **11** in $CDCl_3$. Asterisk means residual solvents or impurity.

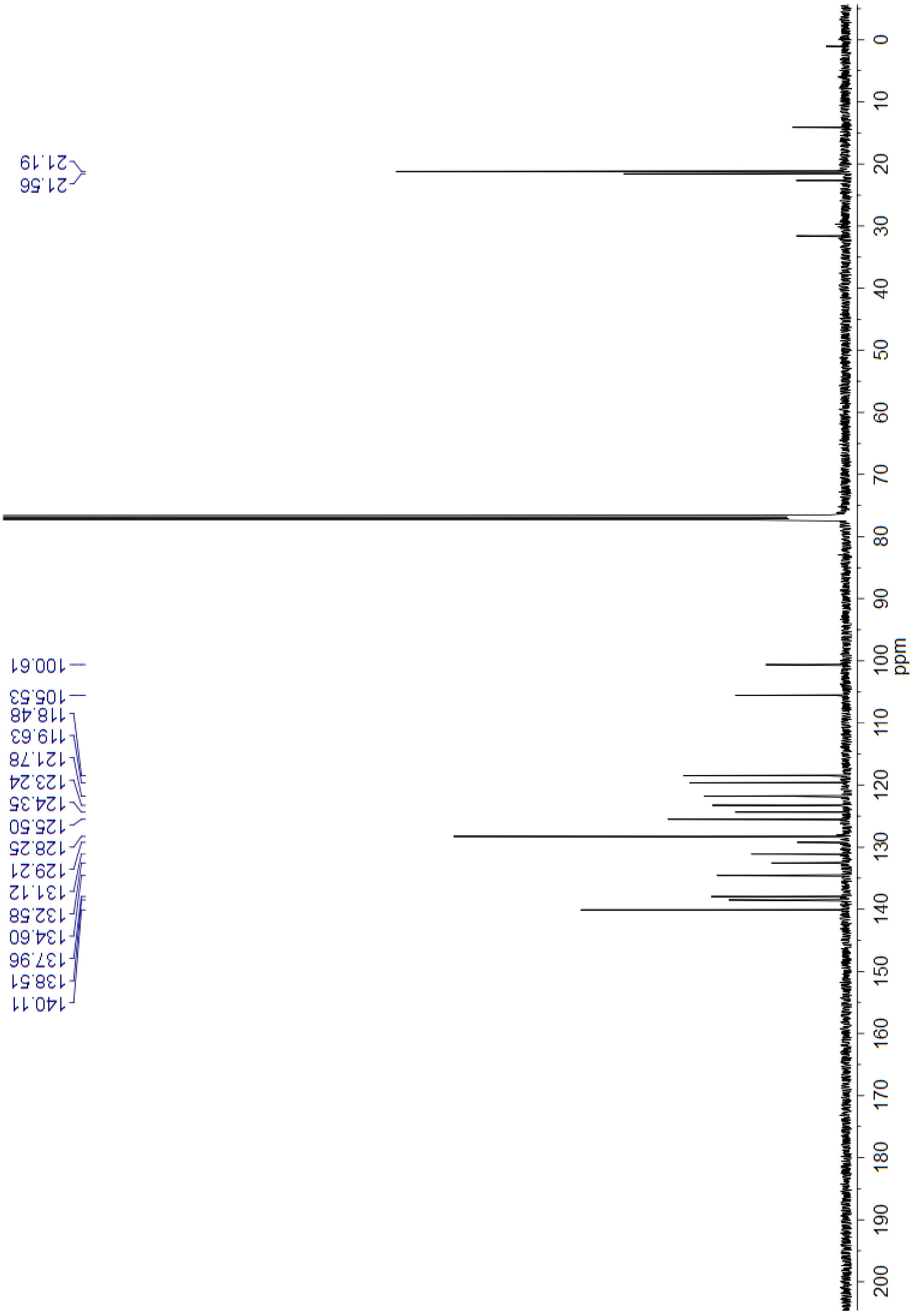

Supplementary Figure 5. ^{13}C NMR spectrum of **11** in $CDCl_3$.

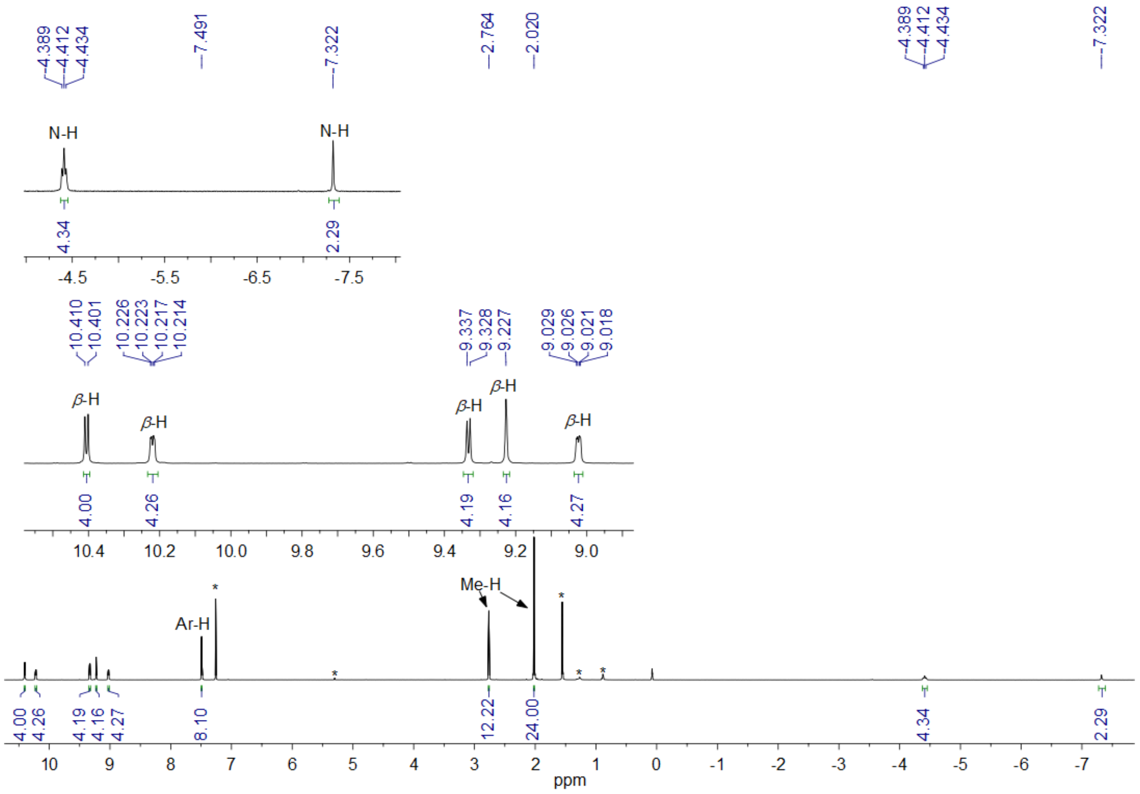

Supplementary Figure 6. ^{1}H NMR spectrum of **12** in $CDCl_3$. Asterisk means residual solvents or impurity.

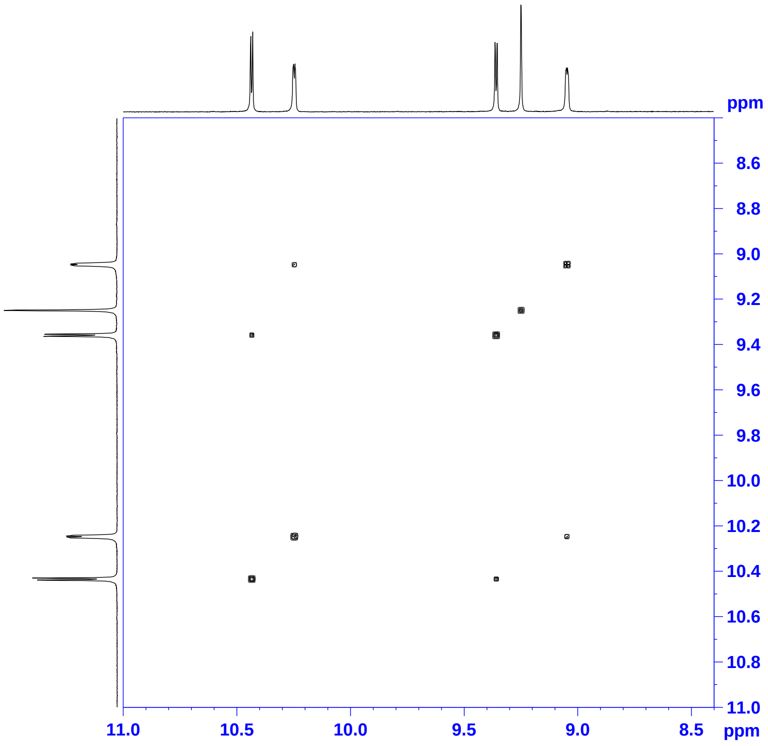

Supplementary Figure 7. H-H COSY spectrum of **12** in $CDCl_3$.

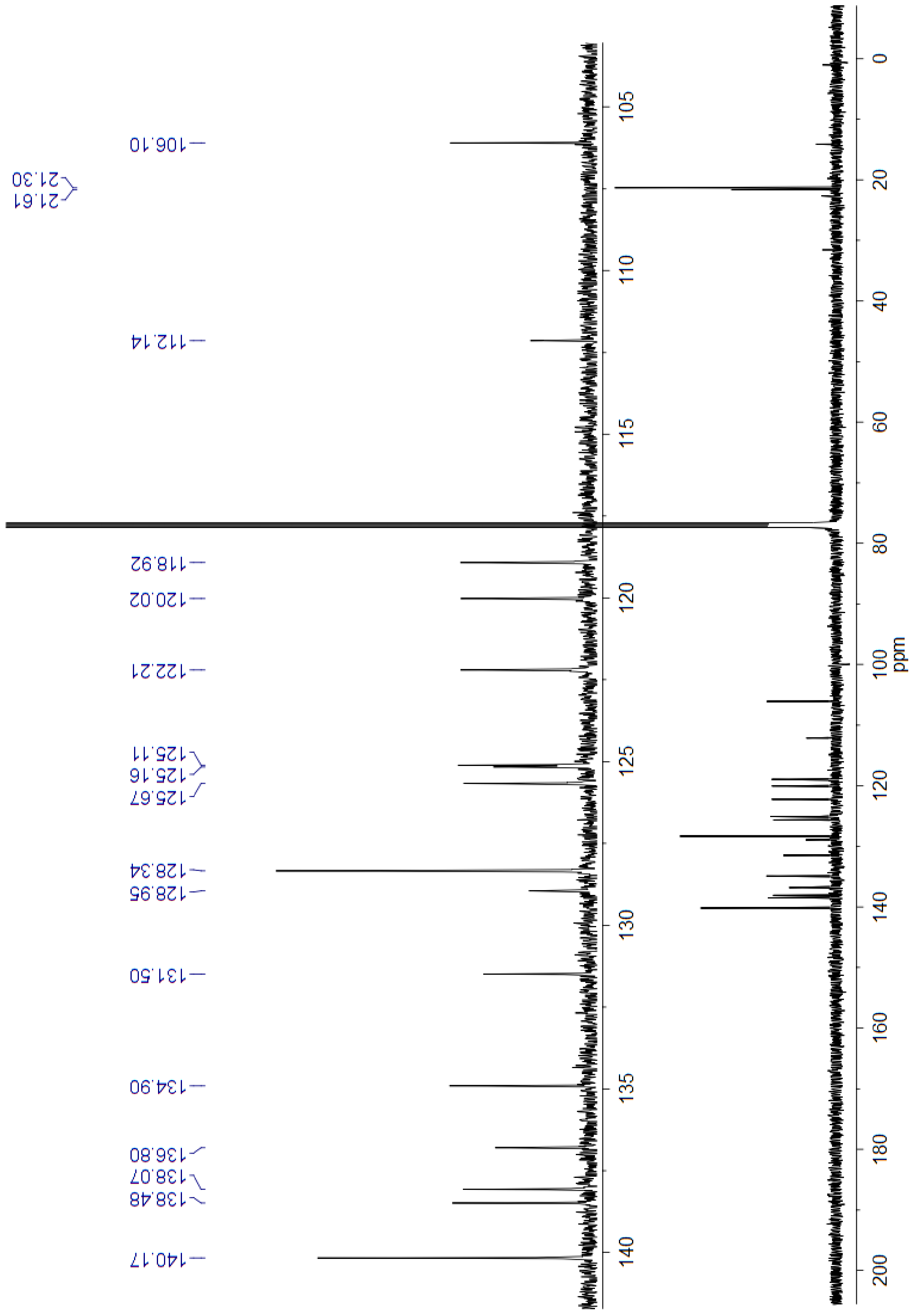

Supplementary Figure 8. ^{13}C NMR spectrum of **12** in $CDCl_3$.

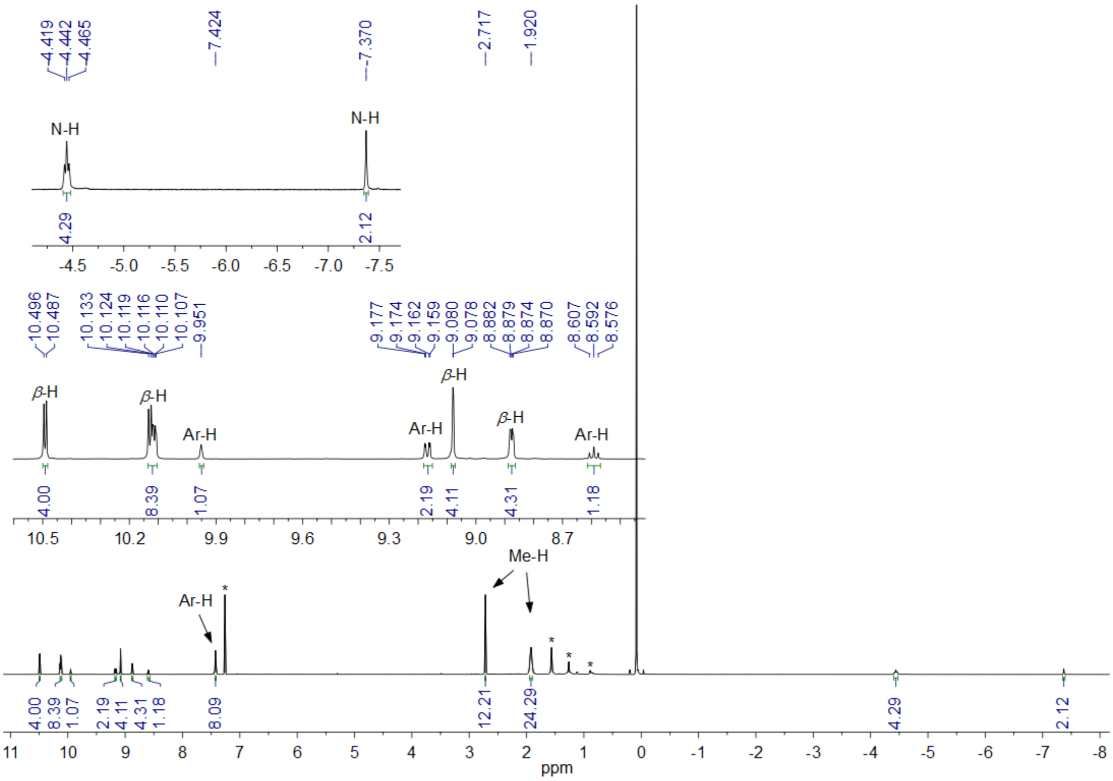

Supplementary Figure 9. ^{1}H NMR spectrum of **6a** in $CDCl_3$. Asterisk means residual solvents or impurity.

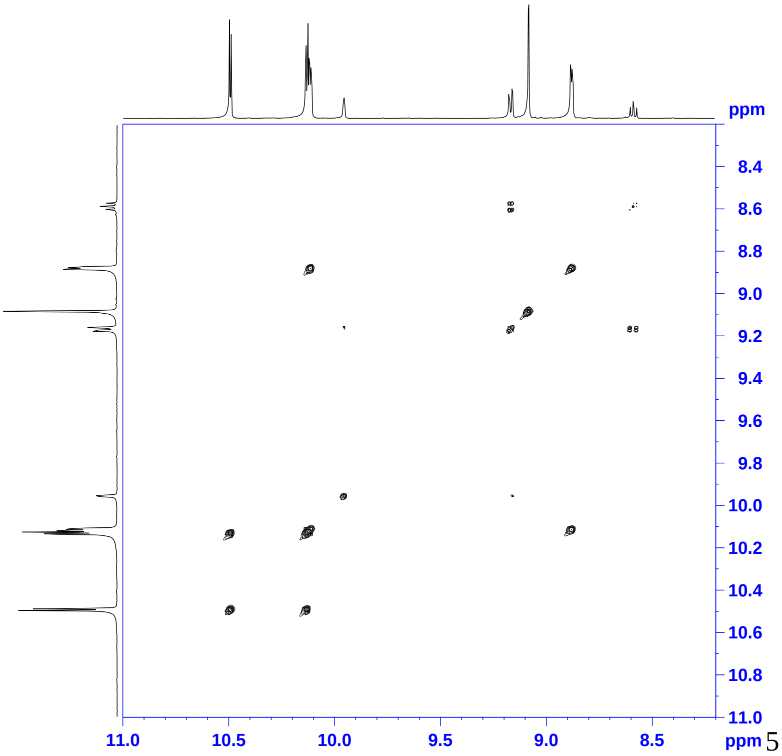

Supplementary Figure 10. H-H COSY spectrum of **6a** in $CDCl_3$.

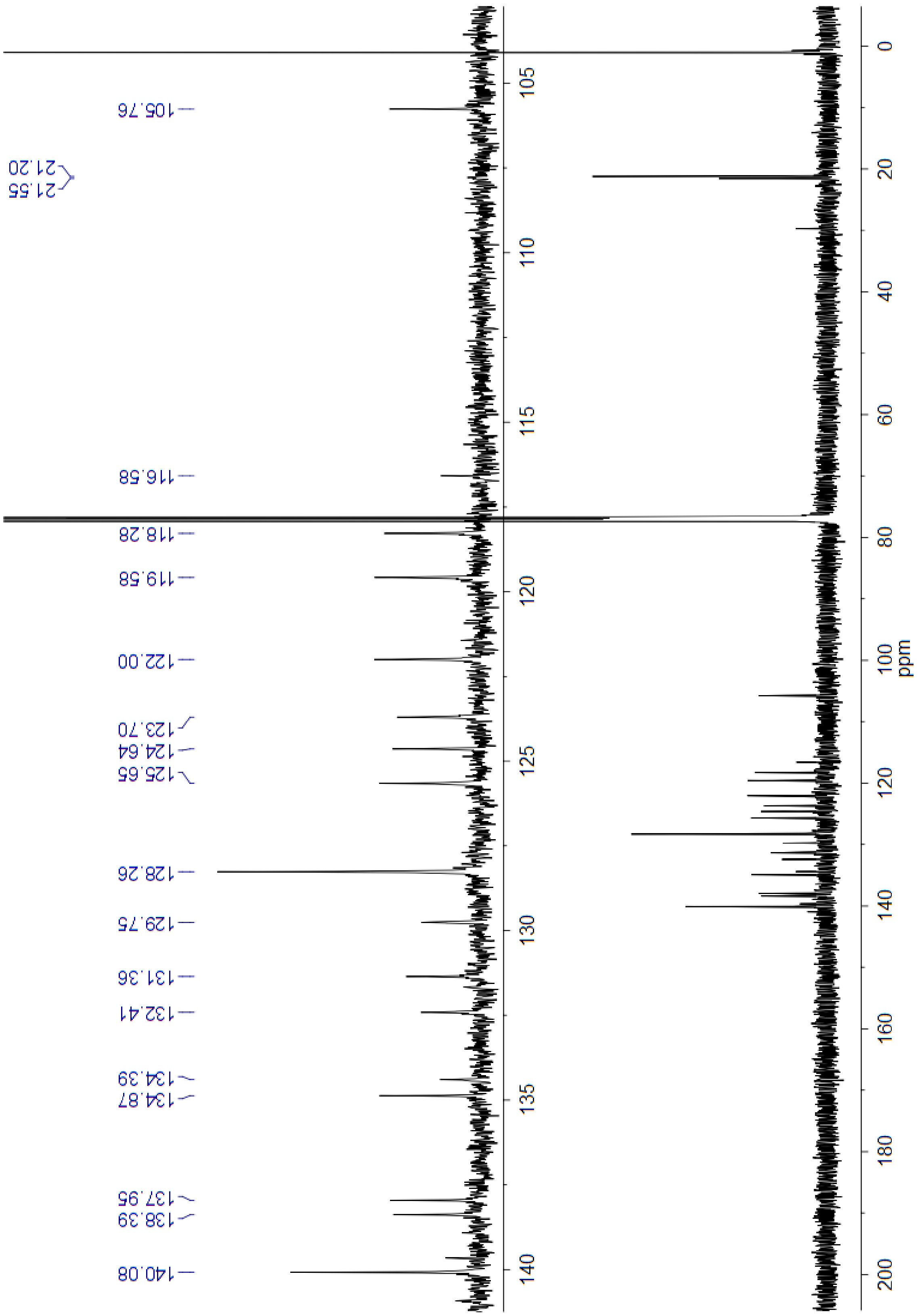

Supplementary Figure 11. ^{13}C NMR spectrum of **6a** in $CDCl_3$.

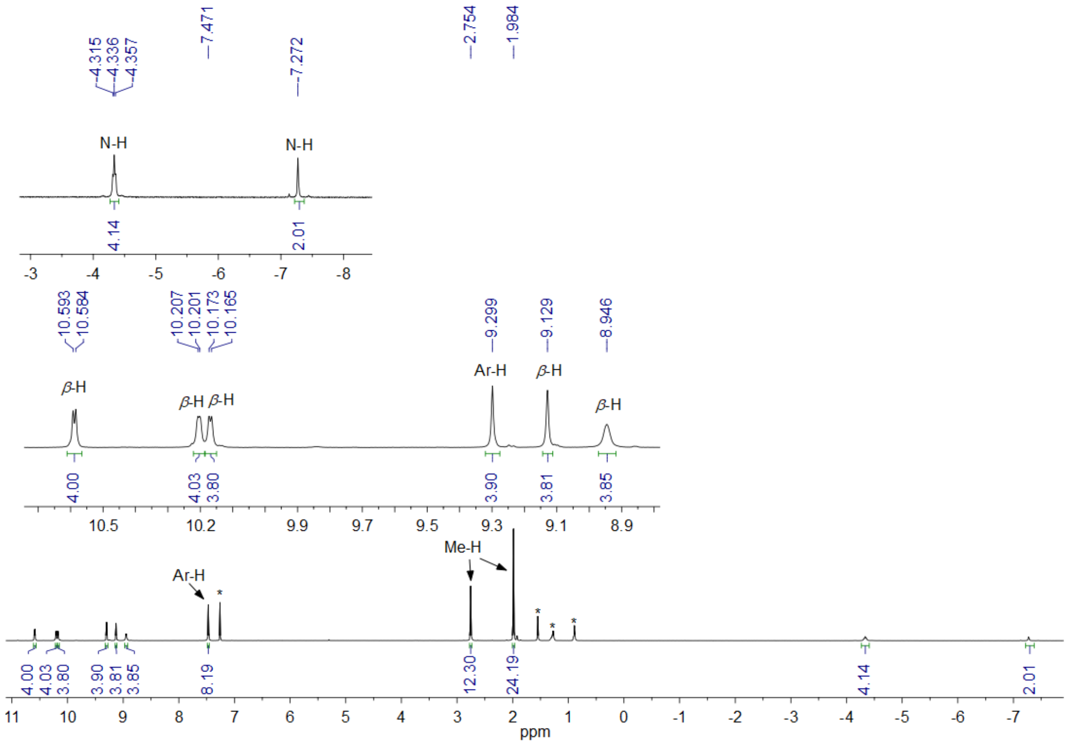

Supplementary Figure 12. ^{1}H NMR spectrum of **6b** in $CDCl_3$. Asterisk means residual solvents or impurity.

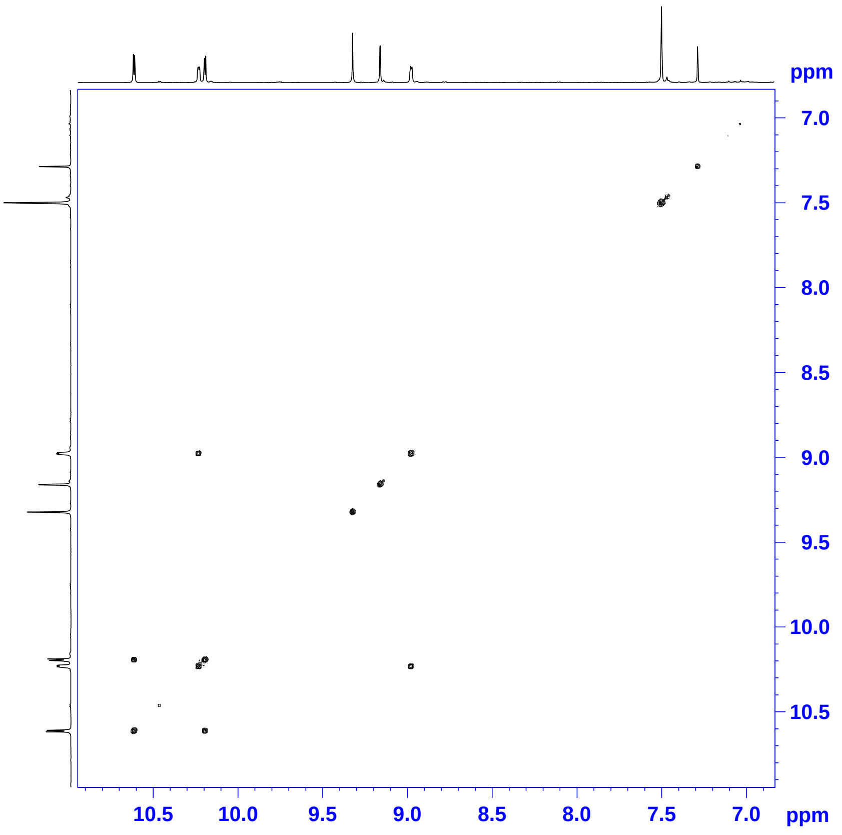

Supplementary Figure 13. H-H COSY spectrum of **6b** in $CDCl_3$.

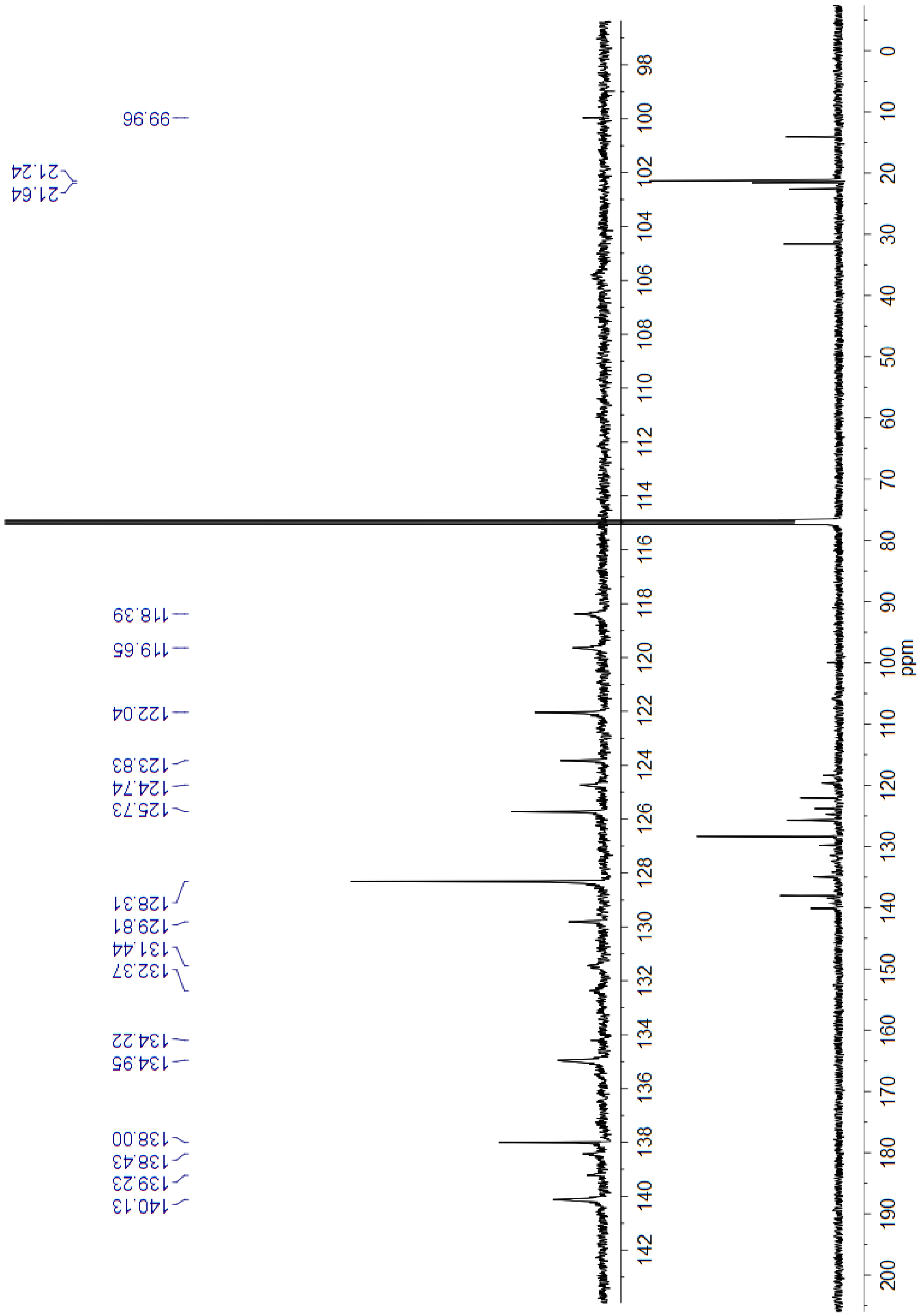

Supplementary Figure 14. ^{13}C NMR spectrum of **6b** in $CDCl_3$.

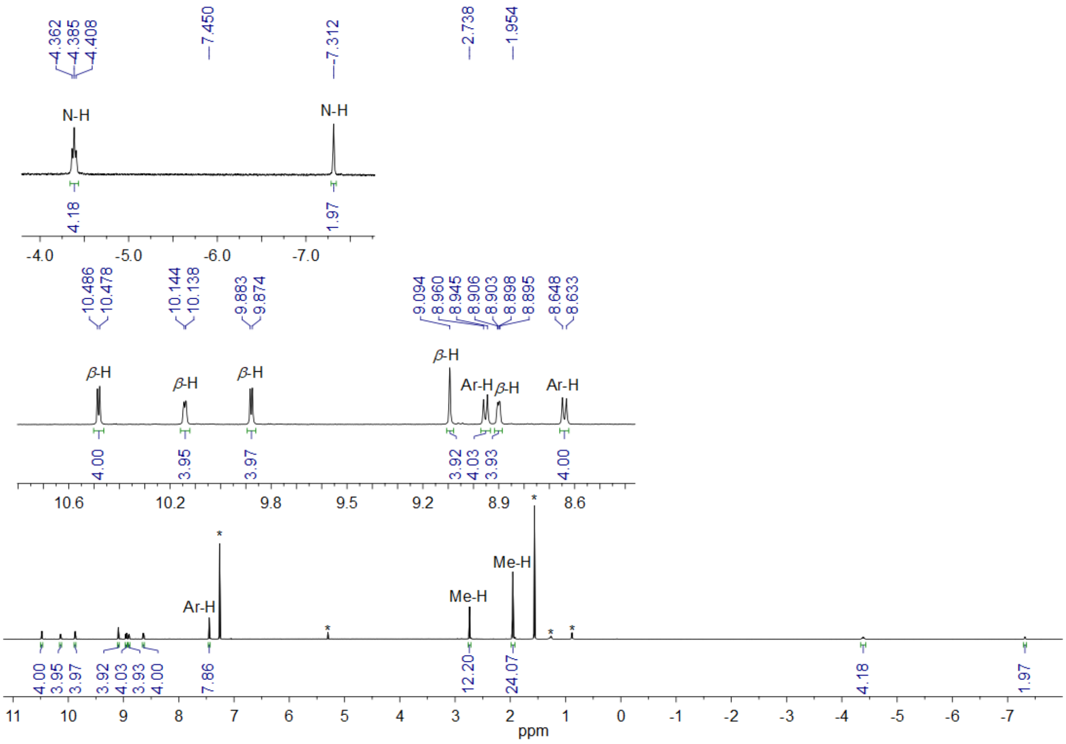

Supplementary Figure 15. ^{1}H NMR spectrum of **6c** in $CDCl_3$.

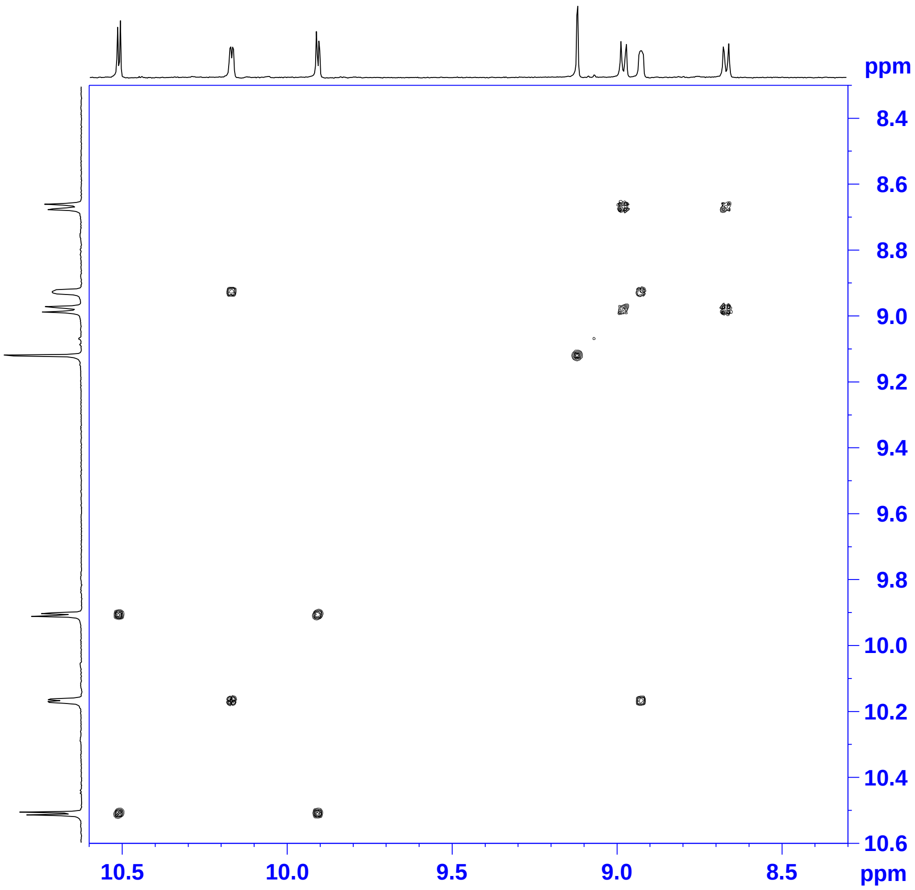

Supplementary Figure 16. H-H COSY spectrum of **6c** in $CDCl_3$.

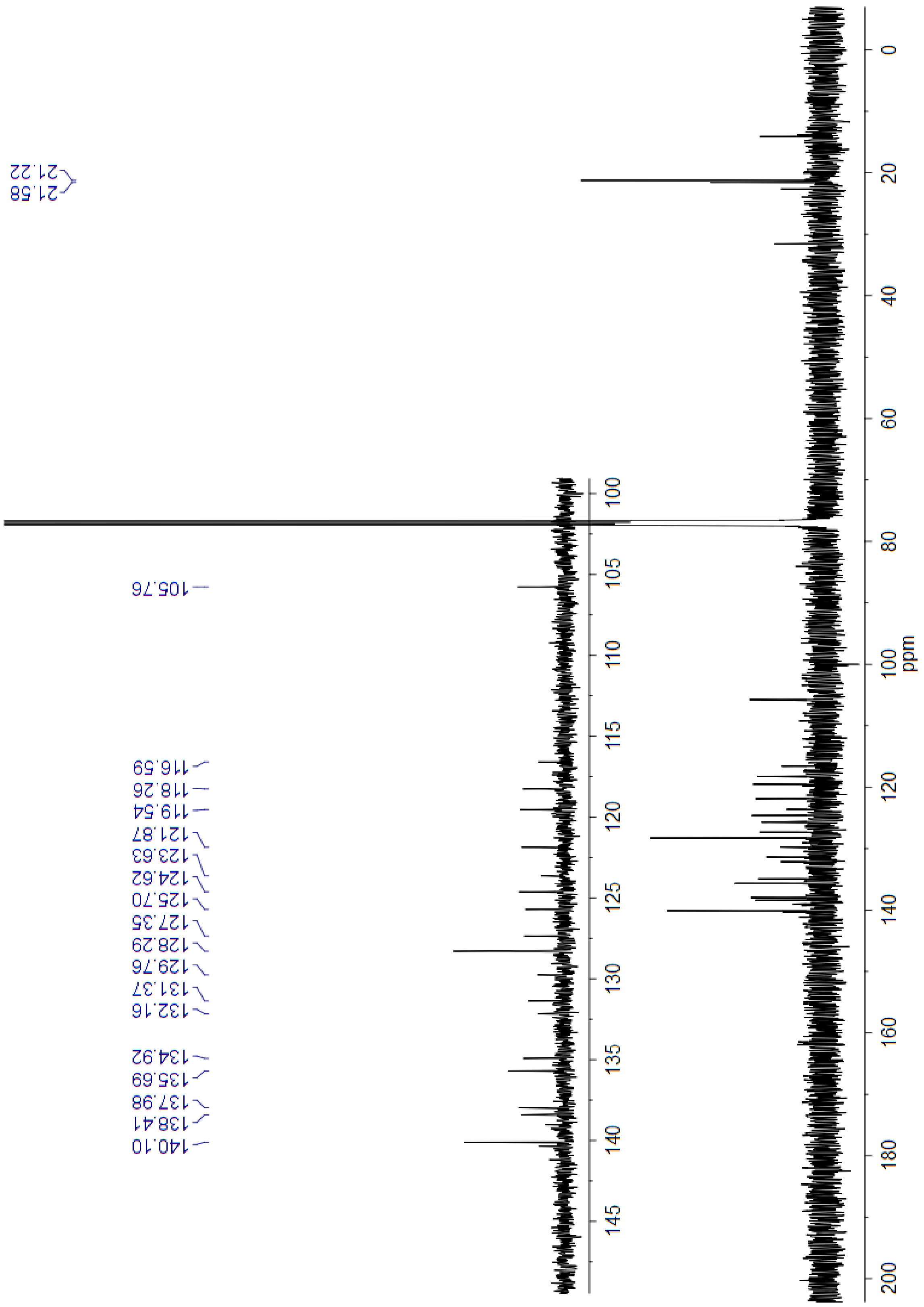

Supplementary Figure 17. ^{13}C NMR spectrum of **6c** in $CDCl_3$.

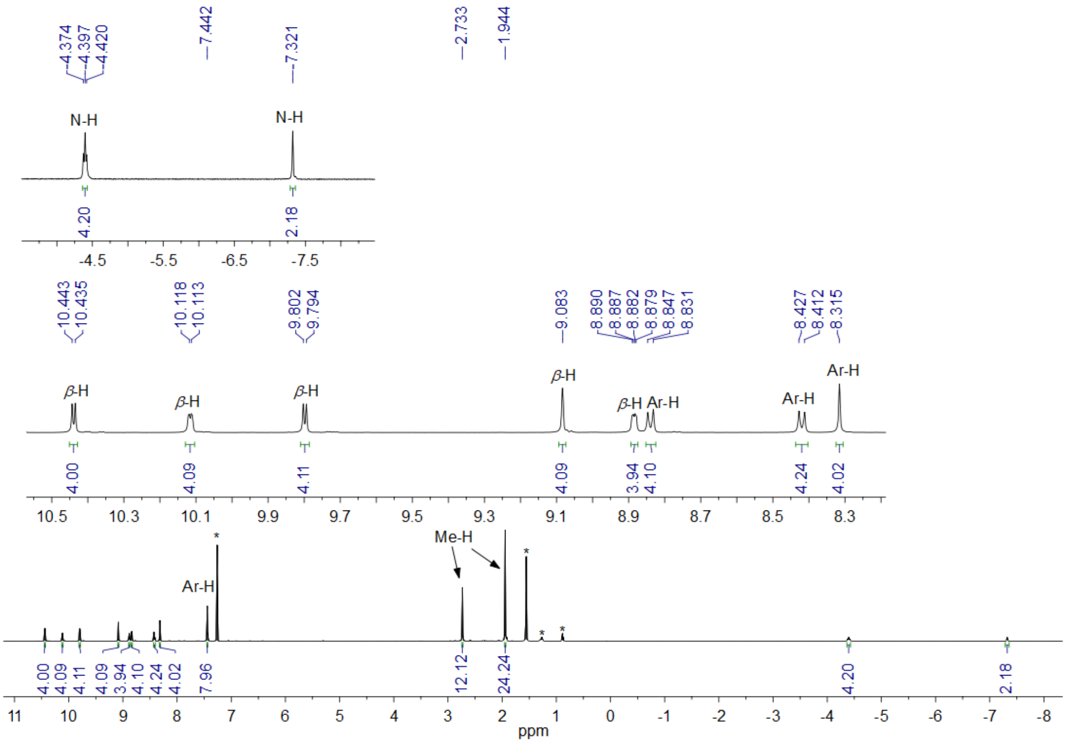

Supplementary Figure 18. ^{1}H NMR spectrum of **6d** in $CDCl_3$.

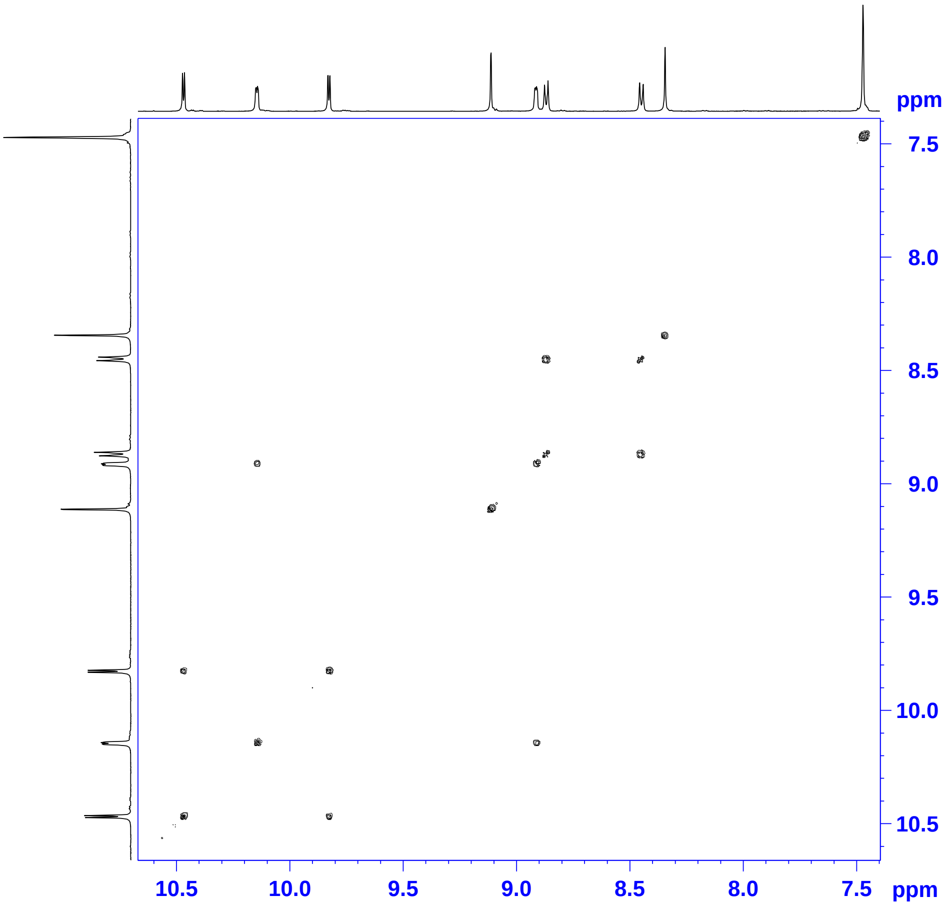

Supplementary Figure 19. H-H COSY spectrum of **6d** in $CDCl_3$.

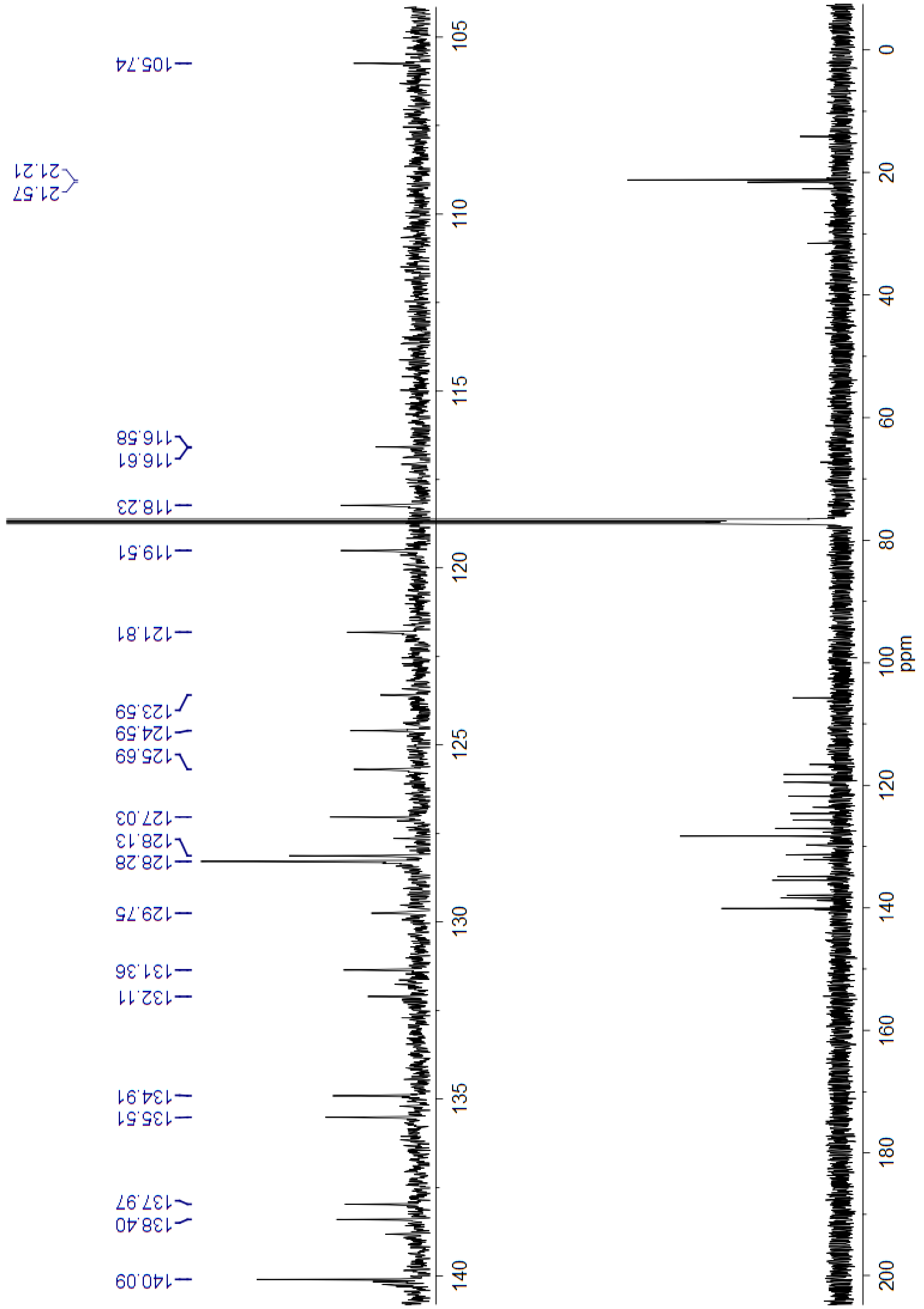

Supplementary Figure 20. ^{13}C NMR spectrum of **6d** in $CDCl_3$.

2.<u>UV/vis absorption spectra and Fluorescence spectra</u>

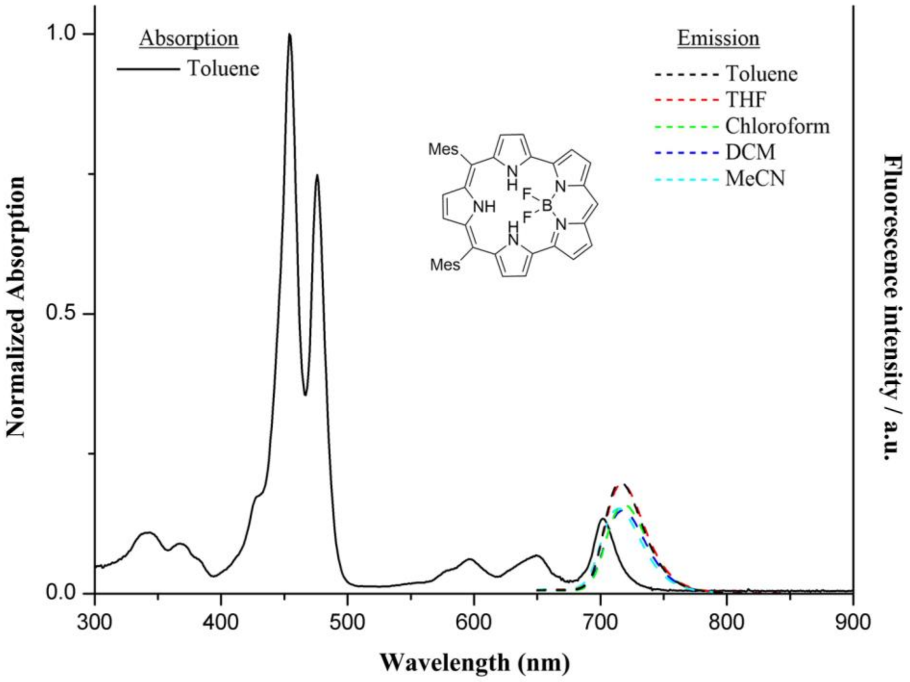

Supplementary Figure 21. UV/vis absorption spectra of **11** in Toluene and Fluorescence emission spectra of **11** in different solvents.

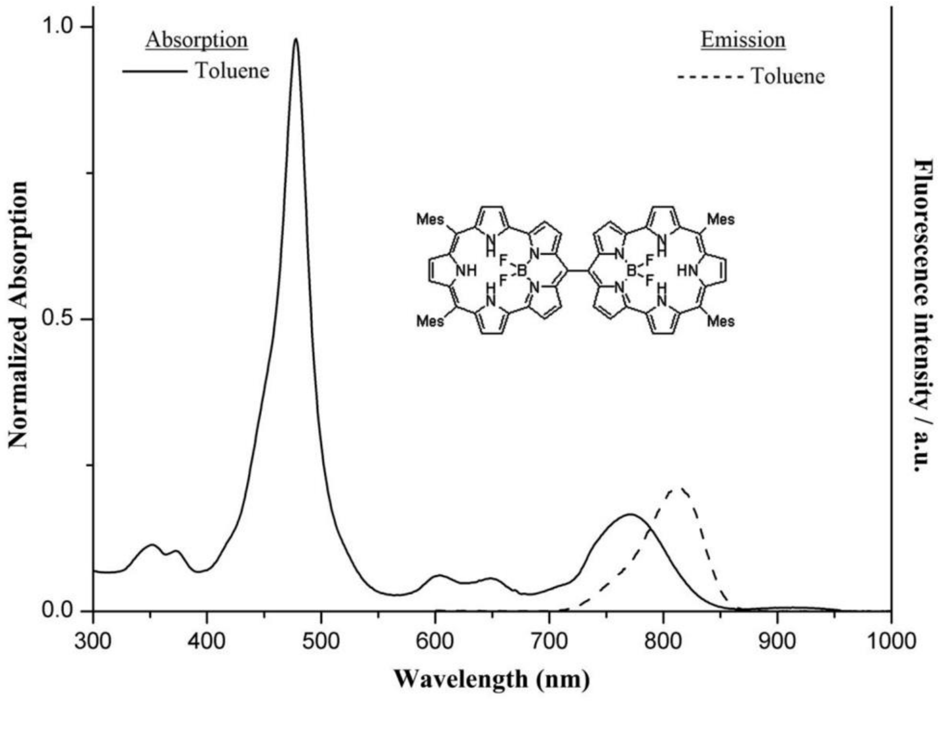

Supplementary Figure 22. UV/vis absorption and Fluorescence emission spectra of **12** in Toluene.

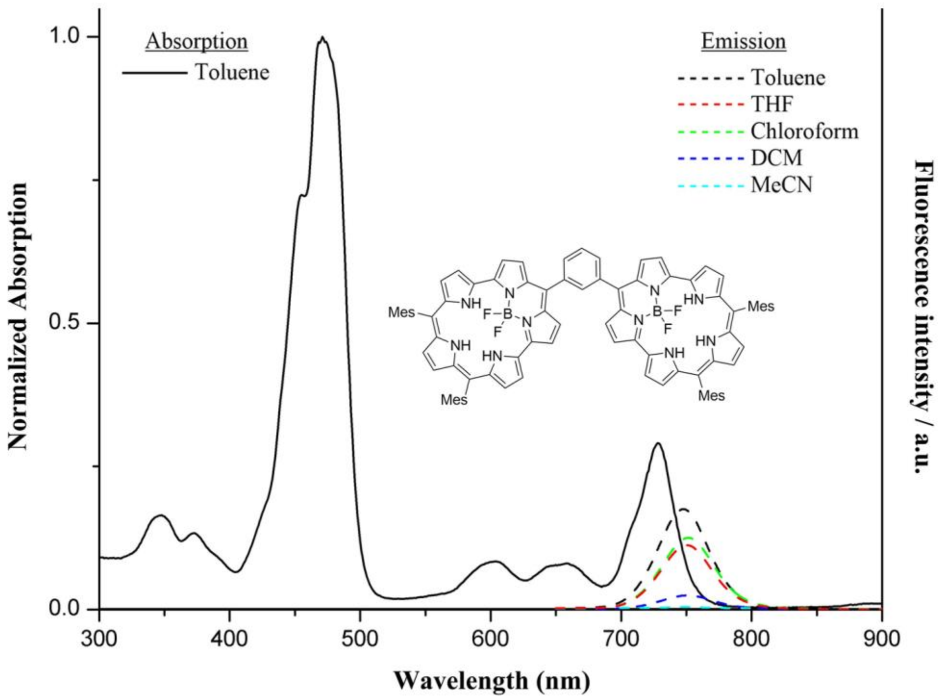

Supplementary Figure 23. UV/vis absorption spectra of **6a** in Toluene and Fluorescence emission spectra of **6a** in different solvents.

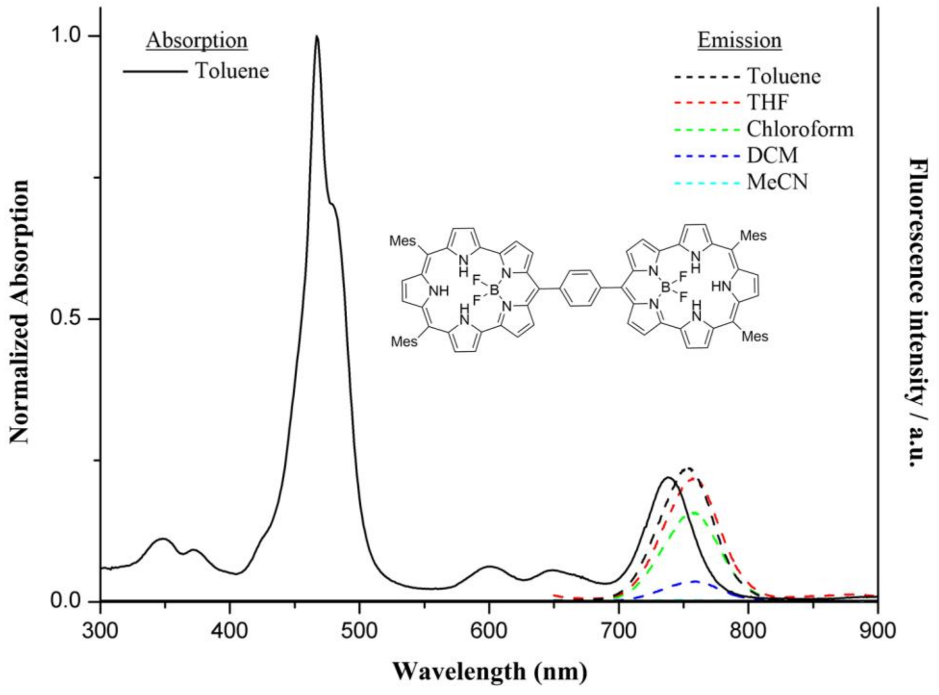

Supplementary Figure 24. UV/vis absorption spectra of **6b** in Toluene and Fluorescence emission spectra of **6b** in different solvents

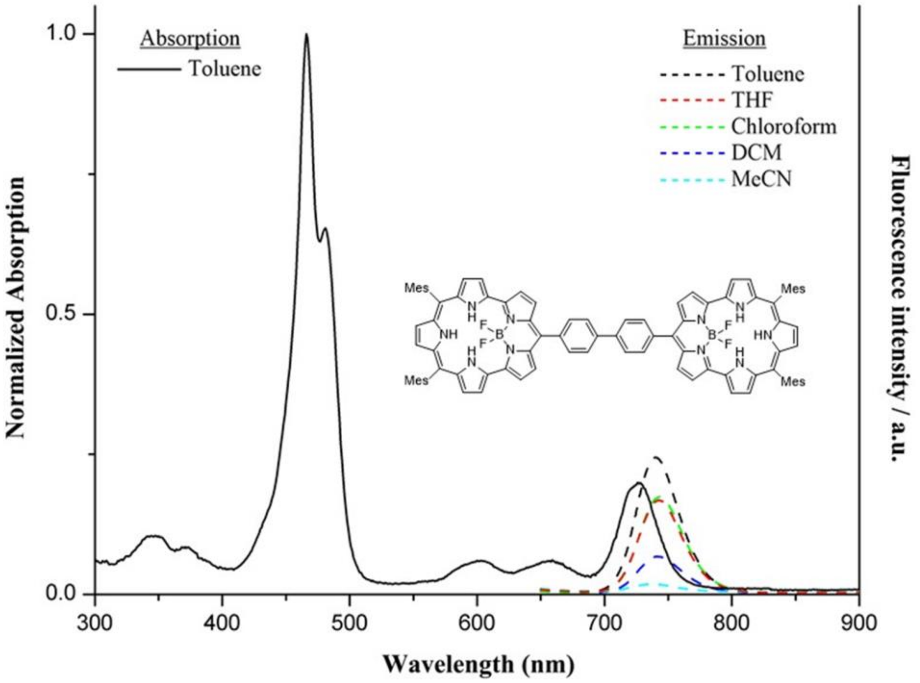

Supplementary Figure 25. UV/vis absorption spectra of **6c** in Toluene and Fluorescence emission spectra of **6c** in different solvents.

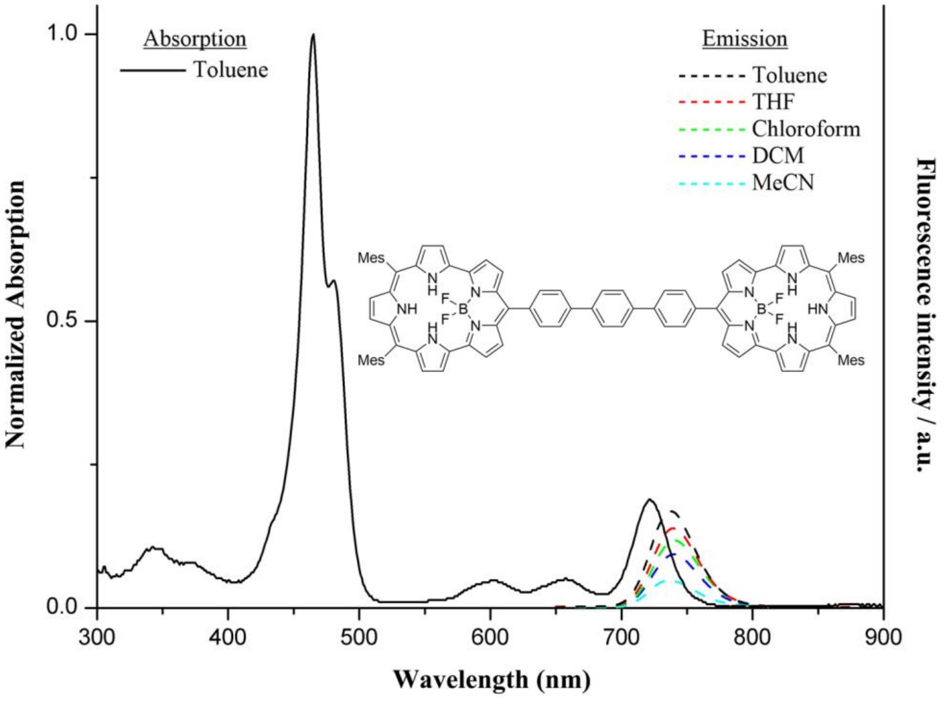

Supplementary Figure 26. UV/vis absorption spectra of **6d** in Toluene and Fluorescence emission spectra of **6d** in different solvents.

3.Electrochemical Data

Supplementary Table 1. CV and DPV of **11**, **12, 6a, 6b, 6c** and **6d** in CH_2Cl_2 with 0.1 M $n$$Bu_4NPF_6$. Potentials were determined vs ferrocene/ferrocenium ion by differential pulse voltammograms. Working electrode: glassy carbon; Counter electrode: Pt wire. Reference electrode: Ag/0.01 M $AgNO_3$.

Sample	$E_{ox.3}$ / V	$E_{ox.2}$ / V	$E_{ox.1}$ / V	$E_{red.1}$ / V	$\triangle E_{HL}$ / eV
**11**	0.97	0.53	0.00	-1.76	1.76
**12**	0.32	0.10	-0.06	-1.62	1.56
**6a**	1.00	0.60	0.02	-1.66	1.68
**6b**	1.00	0.52	0.00	-1.65	1.65
**6c**	0.94	0.44	0.00	-1.66	1.66
**6d**	0.95	0.51	-0.02	-1.66	1.64

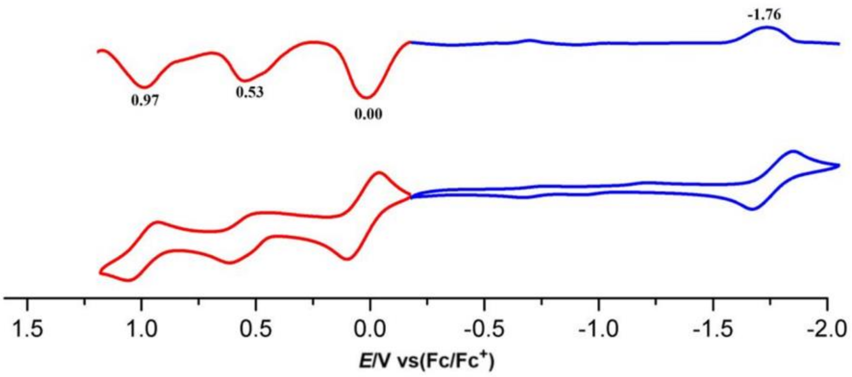

Supplementary Figure 27. Cyclic voltammogram and differential pulse voltammogram of **11**.

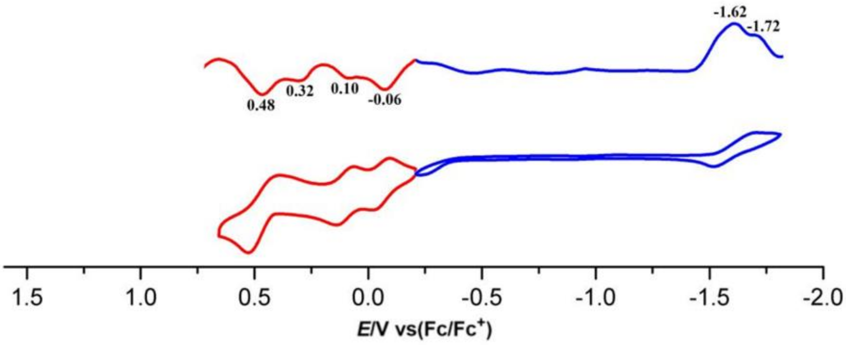

Supplementary Figure 28. Cyclic voltammogram and differential pulse voltammogram of **12**.

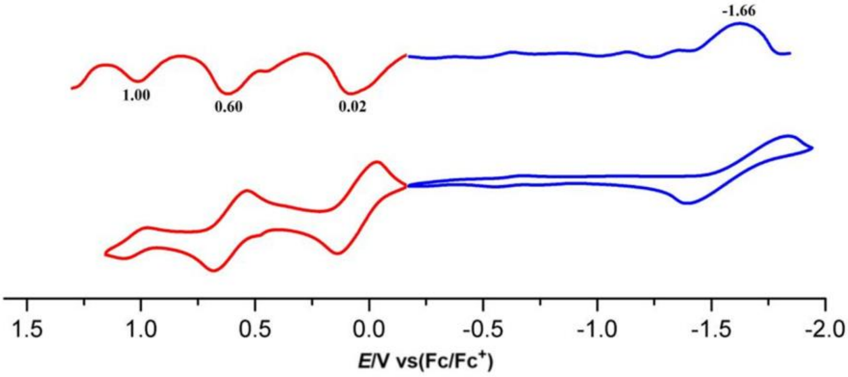

Supplementary Figure 29. Cyclic voltammogram and differential pulse voltammogram of **6a**.

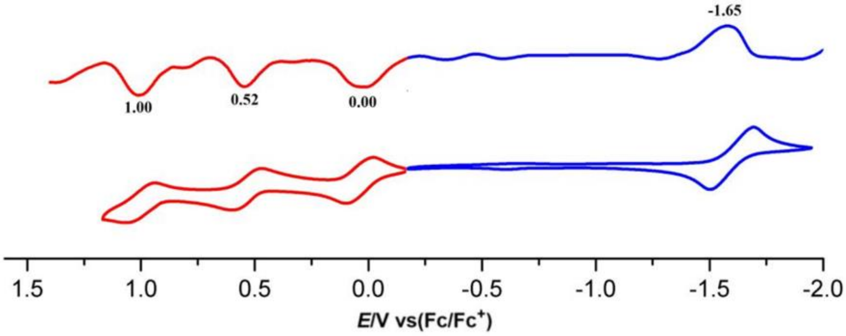

Supplementary Figure 30. Cyclic voltammogram and differential pulse voltammogram of **6b**.

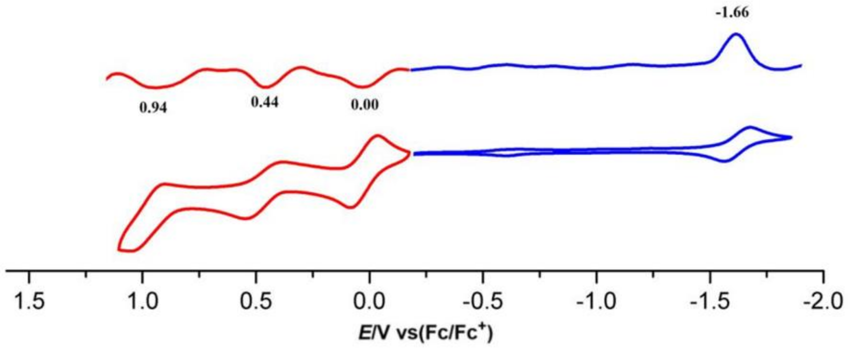

Supplementary Figure 31. Cyclic voltammogram and differential pulse voltammogram of **6c**.

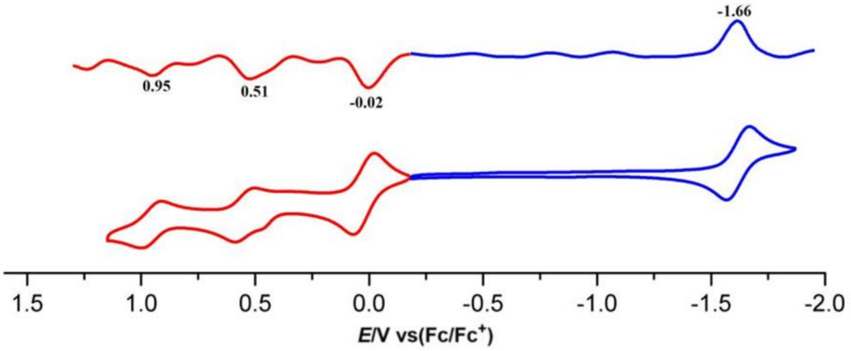

Supplementary Figure 32. Cyclic voltammogram and differential pulse voltammogram of **6d**.

Supplementary Table 2. CV and DPV of **12, 6a, 6b, 6c** and **6d** in PhCN with 0.1 M nBu$_4$NPF$_6$. Potentials were determined vs ferrocene/ferrocenium ion by differential pulse voltammograms. Working electrode: glassy carbon; Counter electrode: Pt wire. Reference electrode: Ag/0.01 M AgNO$_3$.

Sample	$E_{ox.3}$ / V	$E_{ox.2}$ / V	$E_{ox.1}$ / V	$E_{red.1}$ / V	$\triangle E_{HL}$ / eV
**12**	-	0.25	-0.02	-1.53	1.51
**6a**	0.99	0.45	0.05	-1.61	1.66
**6b**	1.03	0.47	0.05	-1.60	1.65
**6c**	0.96	0.44	0.02	-1.60	1.62
**6d**	0.93	0.40	0.02	-1.59	1.61

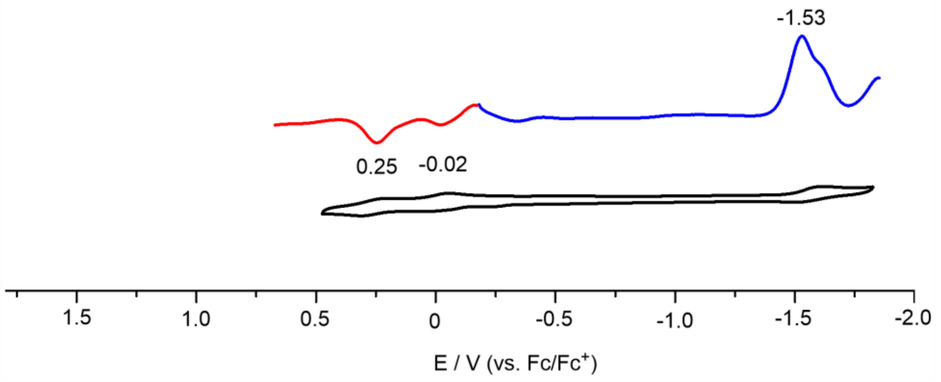

Supplementary Figure 33. Cyclic voltammogram and differential pulse voltammogram of **12**.

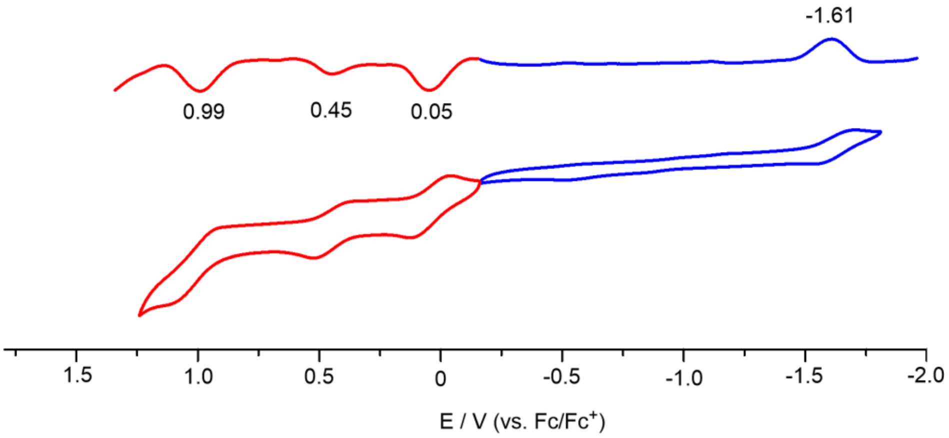

Supplementary Figure 34. Cyclic voltammogram and differential pulse voltammogram of **6a**.

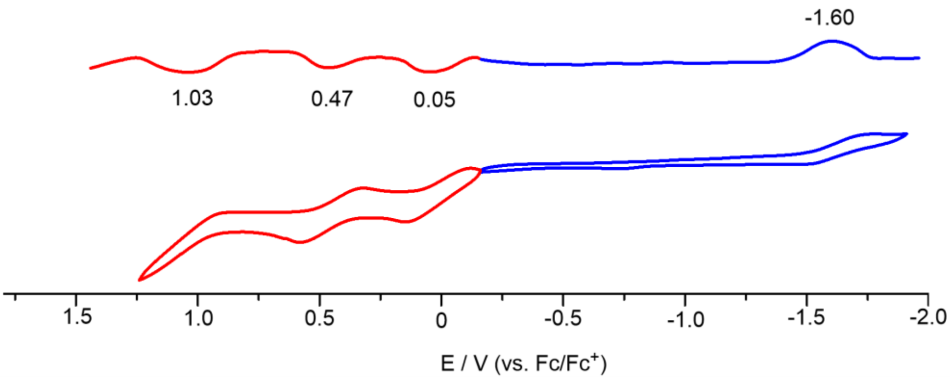

Supplementary Figure 35. Cyclic voltammogram and differential pulse voltammogram of **6b**.

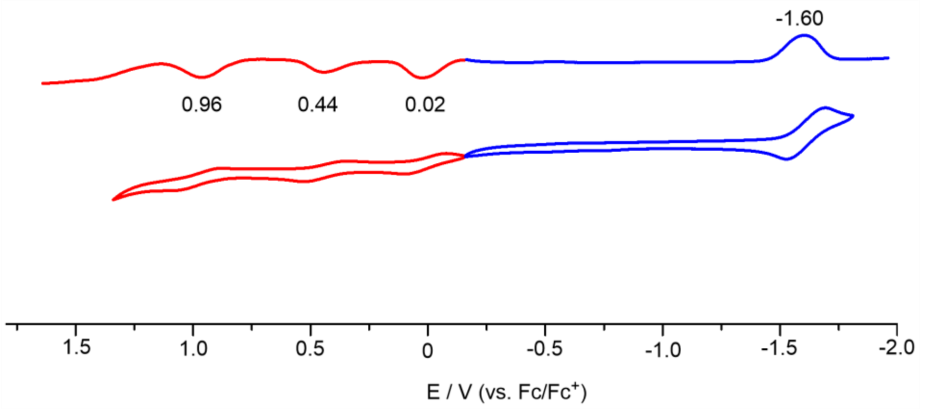

Supplementary Figure 36. Cyclic voltammogram and differential pulse voltammogram of **6c**.

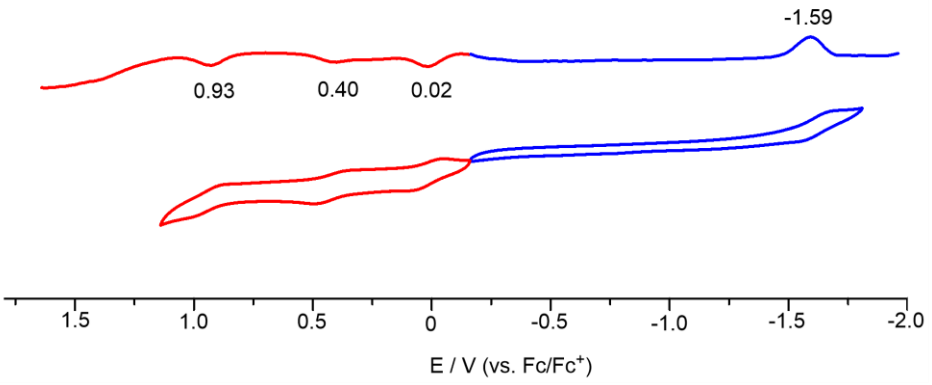

Supplementary Figure 37. Cyclic voltammogram and differential pulse voltammogram of **6d**.

4. X-Ray Crystal Data

a)

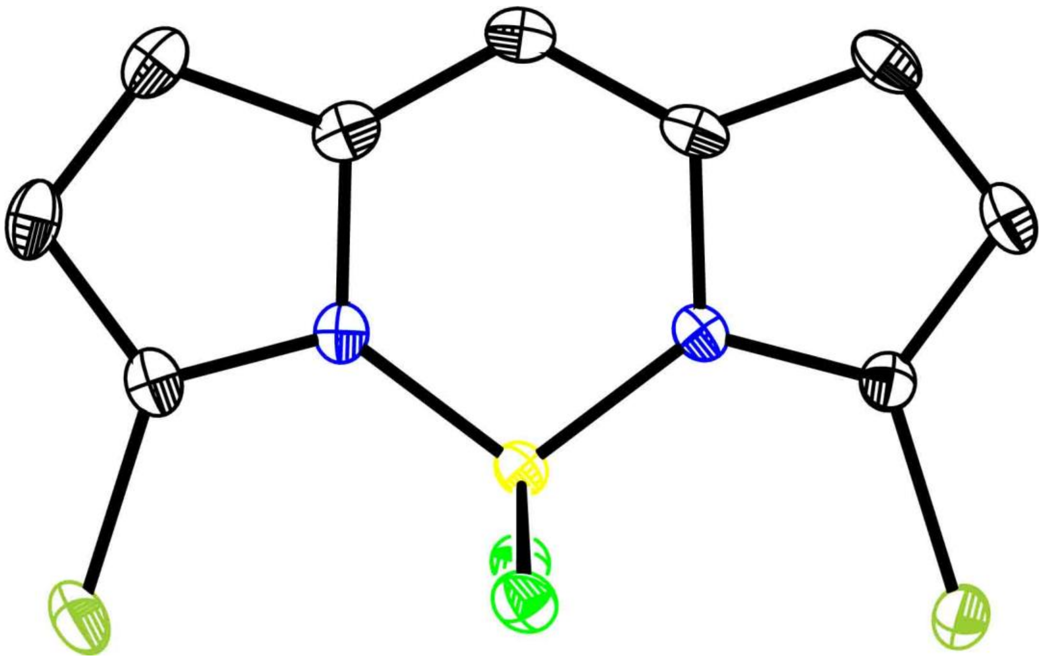

b)

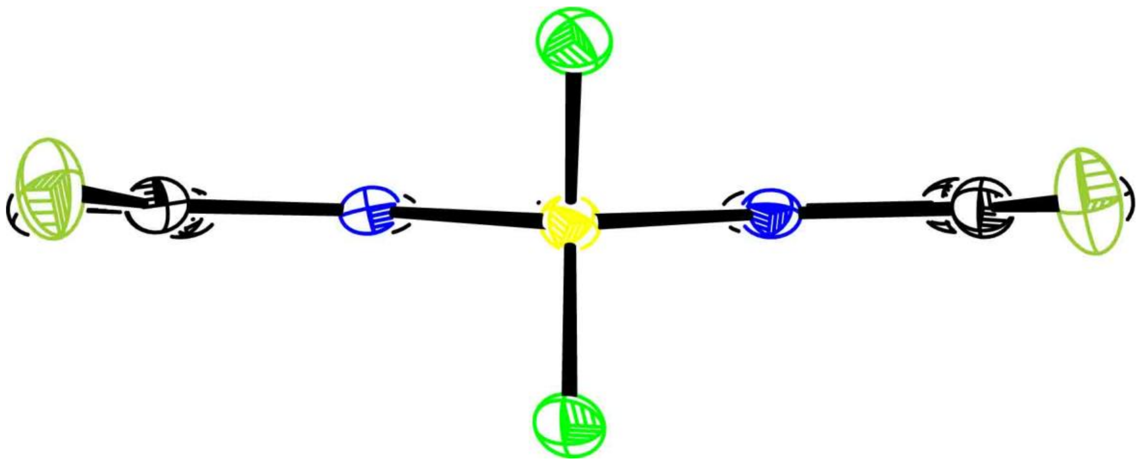

Supplementary Figure 38. X-ray crystal structure of **10**. (a) Top view and (b) side view.

Supplementary Table 3. Crystal data and refinement results for compound **10**

Empirical formula	C9 H5 B Cl2 F2 N2	
Formula weight	260.86	
Temperature	99(2) K	
Wavelength	1.54184 Å	
Crystal system	Monoclinic	
Space group	P 1 21/c 1	
Unit cell dimensions	a = 7.1012(10) Å	a= 90°.
	b = 10.1746(14) Å	b= 96.878(16)°.
	c = 13.888(2) Å	g = 90°.
Volume	996.2(3) $Å^3$	
Z	4	
Density (calculated)	1.739 Mg/m^3	
Absorption coefficient	5.893 mm^{-1}	
F(000)	520	
Crystal size	0.3 x 0.1 x 0.1 mm^3	
Theta range for data collection	5.403 to 66.592°.	
Index ranges	-4<=h<=8, -12<=k<=10, -16<=l<=13	
Reflections collected	3108	
Independent reflections	1750 [R(int) = 0.0326]	
Completeness to theta = 66.592?	99.4 %	
Absorption correction	Semi-empirical from equivalents	
Max. and min. transmission	1.00000 and 0.30595	
Refinement method	Full-matrix least-squares on F^2	
Data / restraints / parameters	1750 / 0 / 145	
Goodness-of-fit on F^2	1.052	
Final R indices [I>2sigma(I)]	R1 = 0.0442, wR2 = 0.1198	
R indices (all data)	R1 = 0.0453, wR2 = 0.1222	
Extinction coefficient	n/a	
Largest diff. peak and hole	0.441 and -0.486 e.$Å^{-3}$	
CCDC number	2183009	

a)

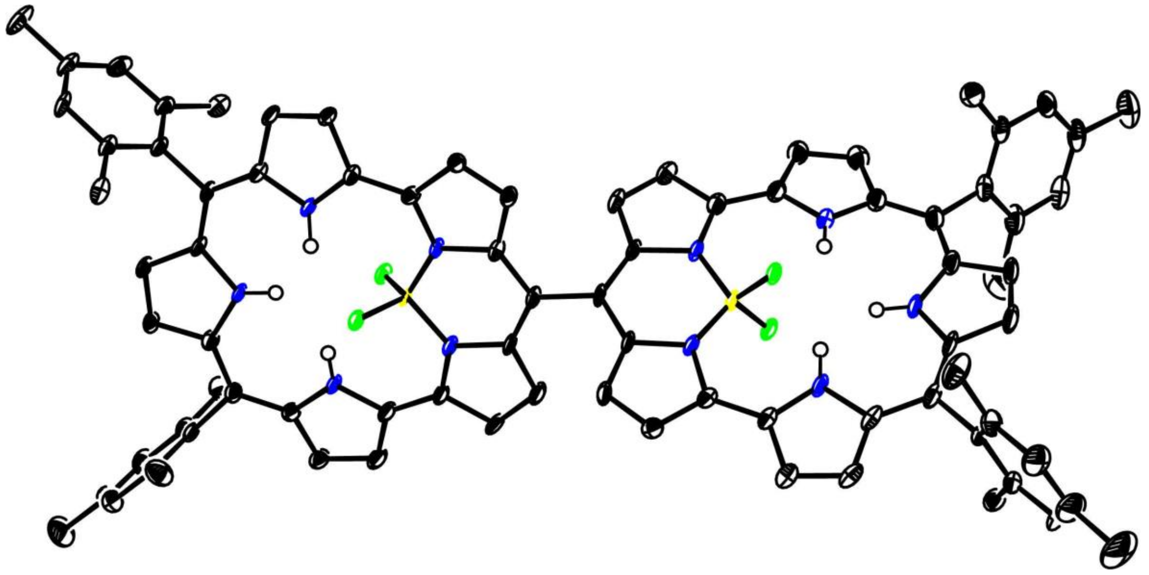

b)

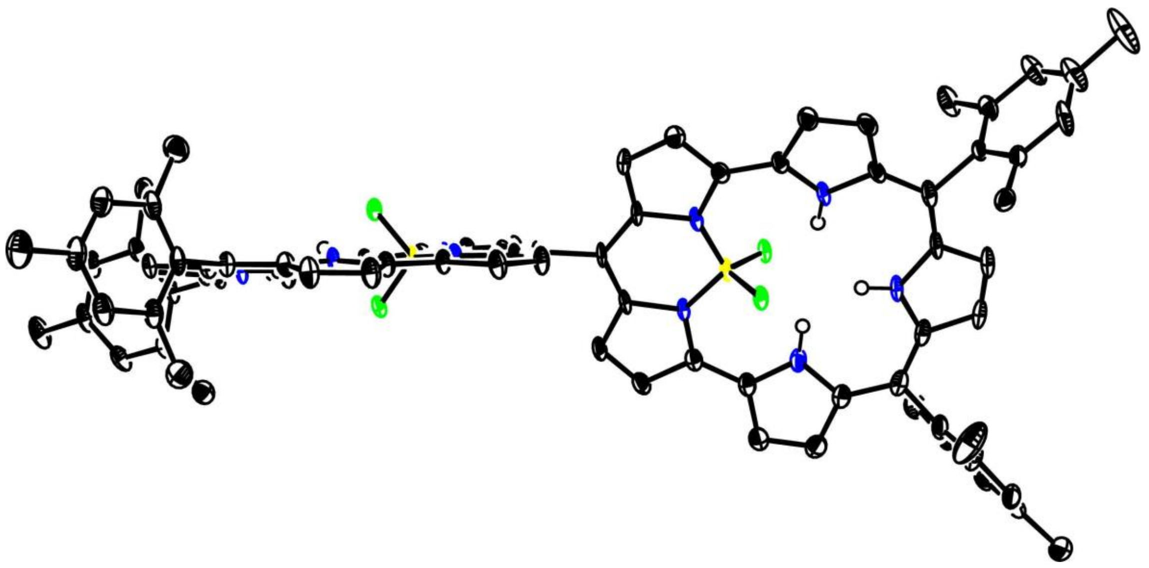

Supplementary Figure 39. X-ray crystal structure of **12**. (a) Top view and (b) side view.

Supplementary Table 4. Crystal data and refinement results for compound **12**

Empirical formula	C87 H80 B2 Cl5 F4 N10	
Formula weight	1540.48	
Temperature	100.01(10) K	
Wavelength	1.54184 Å	
Crystal system	Triclinic	
Space group	P-1	
Unit cell dimensions	a = 12.1103(3) Å	a= 104.055(2)°.
	b = 17.8722(5) Å	b= 97.125(2)°.
	c = 21.2502(6) Å	g = 101.364(2)°.
Volume	4302.2(2) $Å^3$	
Z	2	
Density (calculated)	1.189 Mg/m^3	
Absorption coefficient	1.993 mm^{-1}	
F(000)	1606	
Crystal size	0.3 x 0.1 x 0.02 mm^3	
Theta range for data collection	2.179 to 66.599°	
Index ranges	-14<=h<=14, -13<=k<=21, -25<=l<=22	
Reflections collected	29241	
Independent reflections	15209 [R(int) = 0.0508]	
Completeness to theta = 66.599?	100.0 %	
Absorption correction	Semi-empirical from equivalents	
Max. and min. transmission	1.00000 and 0.78758	
Refinement method	Full-matrix least-squares on F^2	
Data / restraints / parameters	15209 / 150 / 1090	
Goodness-of-fit on F^2	1.031	
Final R indices [I>2sigma(I)]	R1 = 0.0605, wR2 = 0.1439	
R indices (all data)	R1 = 0.0810, wR2 = 0.1563	
Extinction coefficient	n/a	
Largest diff. peak and hole	1.052 and -0.703 e.$Å^{-3}$	
CCDC number	2183010	

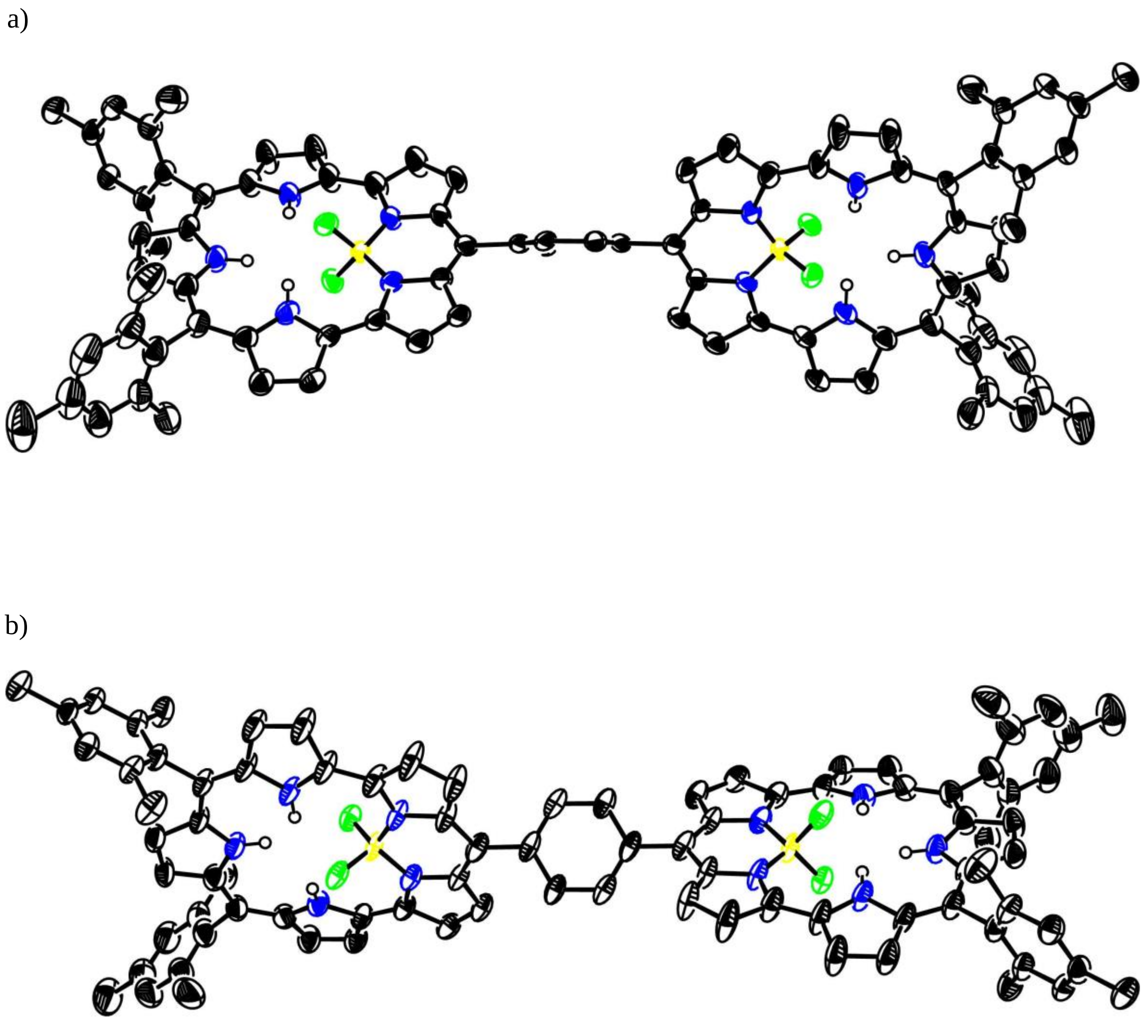

Supplementary Figure 40. X-ray crystal structure of **6b**. (a) Top view and (b) side view.

Supplementary Table 5. Crystal data and refinement results for compound **6b**

Empirical formula	C88 H74 B2 F4 N10	
Formula weight	1369.19	
Temperature	97(2) K	
Wavelength	1.54184 Å	
Crystal system	Monoclinic	
Space group	I 1 2/a 1	
Unit cell dimensions	a = 21.0388(7) Å	a= 90°.
	b = 31.2465(10) Å	b= 105.226(4)°.
	c = 29.3757(10) Å	g = 90°.
Volume	18633.4(11) $Å^3$	
Z	8	
Density (calculated)	0.976 Mg/m^3	
Absorption coefficient	0.505 mm^{-1}	
F(000)	5744	
Crystal size	0.2 x 0.05 x 0.05 mm^3	
Theta range for data collection	3.592 to 66.601°.	
Index ranges	-24<=h<=25, -33<=k<=37, -27<=l<=34	
Reflections collected	56417	
Independent reflections	16448 [R(int) = 0.0793]	
Completeness to theta = 66.601?	99.9 %	
Absorption correction	Semi-empirical from equivalents	
Max. and min. transmission	1.00000 and 0.27252	
Refinement method	Full-matrix least-squares on F^2	
Data / restraints / parameters	16448 / 391 / 972	
Goodness-of-fit on F^2	1.063	
Final R indices [I>2sigma(I)]	R1 = 0.0772, wR2 = 0.2055	
R indices (all data)	R1 = 0.1184, wR2 = 0.2397	
Extinction coefficient	n/a	
Largest diff. peak and hole	0.422 and -0.305 e.$Å^{-3}$	
CCDC number	2183007	

a)

b)

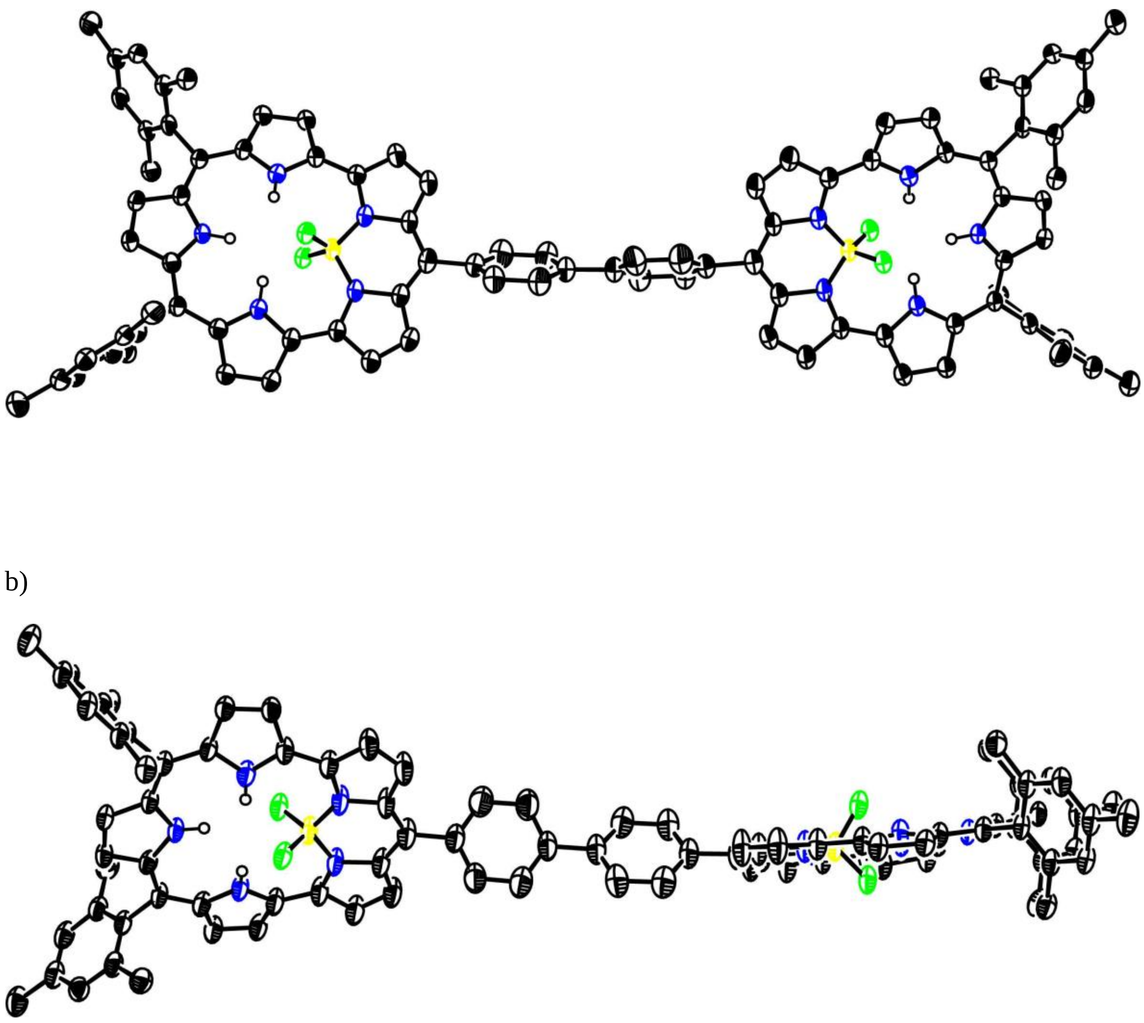

Supplementary Figure 41. X-ray crystal structure of **6c**. (a) Top view and (b) side view.

Supplementary Table 6. Crystal data and refinement results for compound **6c**

Empirical formula	C94 H78 B2 F4 N10	
Formula weight	1445.28	
Temperature	99.98(11) K	
Wavelength	1.54184 Å	
Crystal system	Monoclinic	
Space group	I 1 2/a 1	
Unit cell dimensions	a = 11.3281(7) Å	a= 90°.
	b = 30.8697(15) Å	b= 93.070(4)°.
	c = 32.0384(9) Å	g = 90°.
Volume	11187.6(9) $Å^3$	
Z	4	
Density (calculated)	0.858 Mg/m^3	
Absorption coefficient	0.440 mm^{-1}	
F(000)	3032	
Crystal size	0.3 x 0.02 x 0.02 mm^3	
Theta range for data collection	2.762 to 66.600°	
Index ranges	-8<=h<=13, -36<=k<=36, -37<=l<=38	
Reflections collected	35573	
Independent reflections	9884 [R(int) = 0.0839]	
Completeness to theta = 66.600?	99.9 %	
Absorption correction	Semi-empirical from equivalents	
Max. and min. transmission	1.00000 and 0.37938	
Refinement method	Full-matrix least-squares on F^2	
Data / restraints / parameters	9884 / 36 / 502	
Goodness-of-fit on F^2	1.041	
Final R indices [I>2sigma(I)]	R1 = 0.0820, wR2 = 0.2386	
R indices (all data)	R1 = 0.1239, wR2 = 0.2697	
Extinction coefficient	n/a	
Largest diff. peak and hole	0.477 and -0.354 e.$Å^{-3}$	
CCDC number	2191271	

5. HR-MS Spectra of Compounds

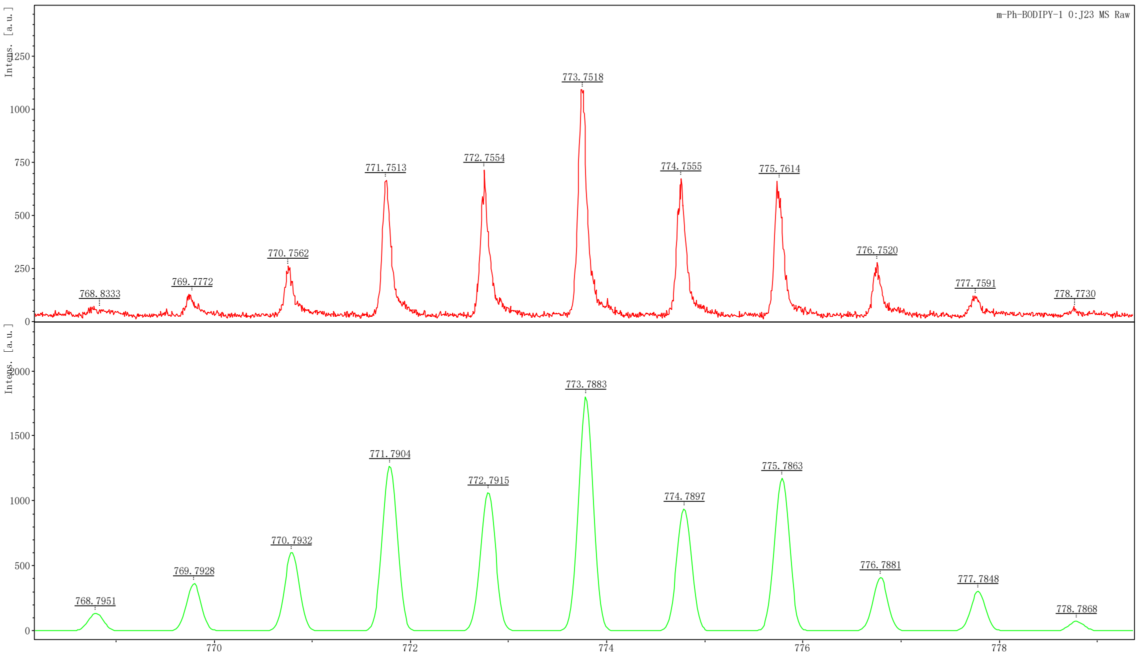

Supplementary Figure 42. MALDI-TOF-MS spectrum of **4a**.

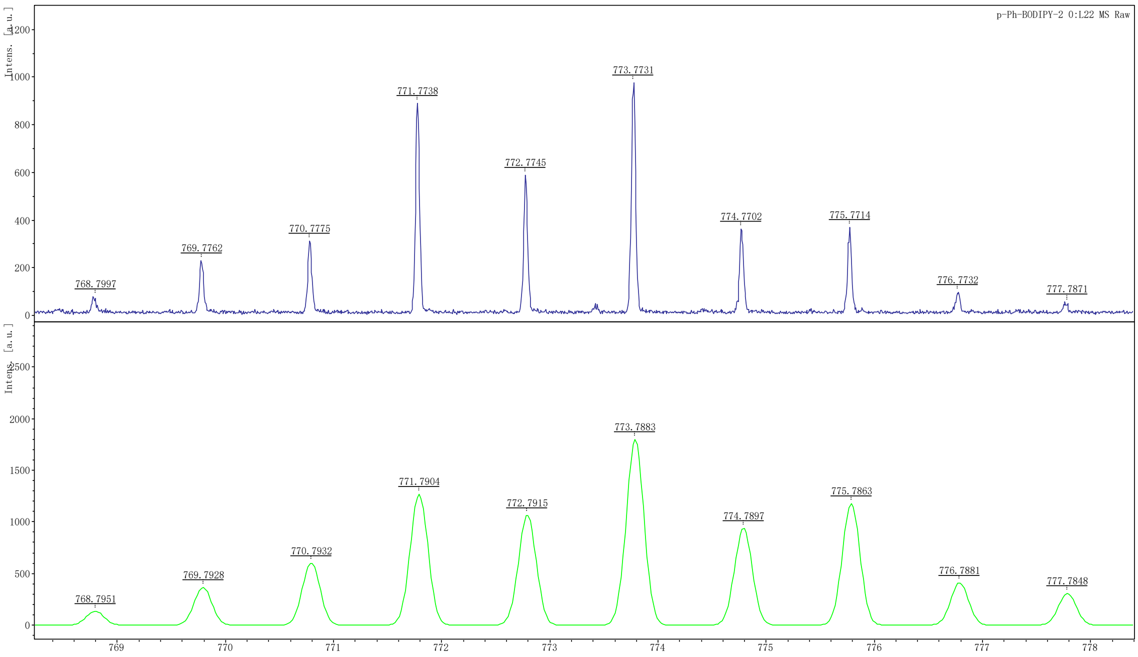

Supplementary Figure 43. MALDI-TOF-MS spectrum of **4b**.

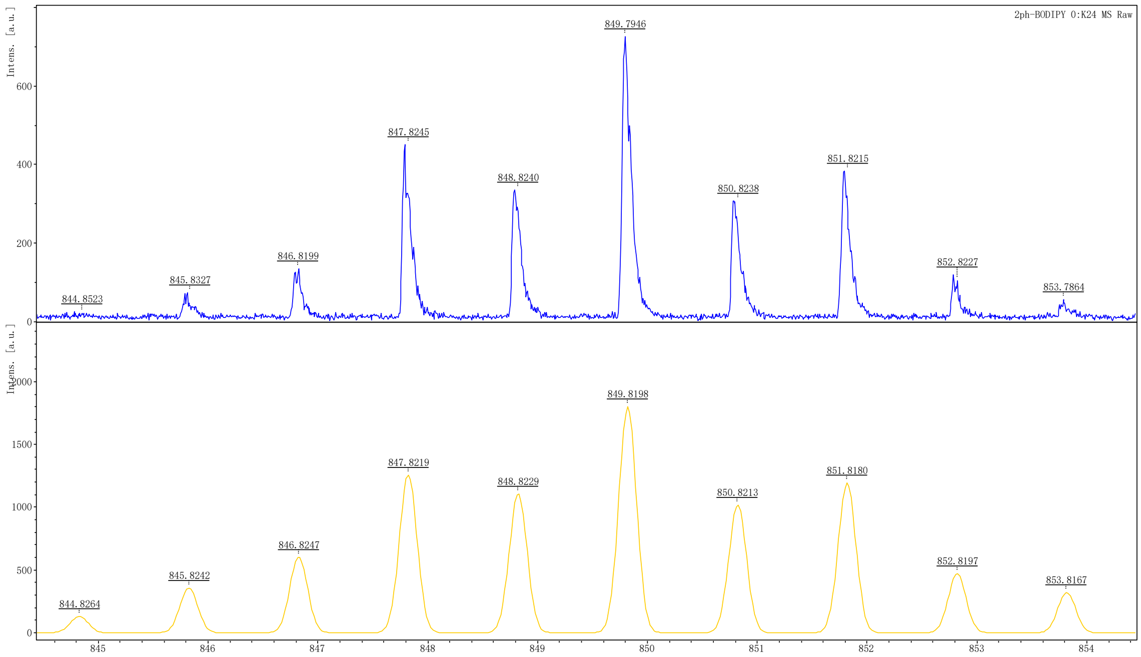

Supplementary Figure 44. MALDI-TOF-MS spectrum of **4c**.

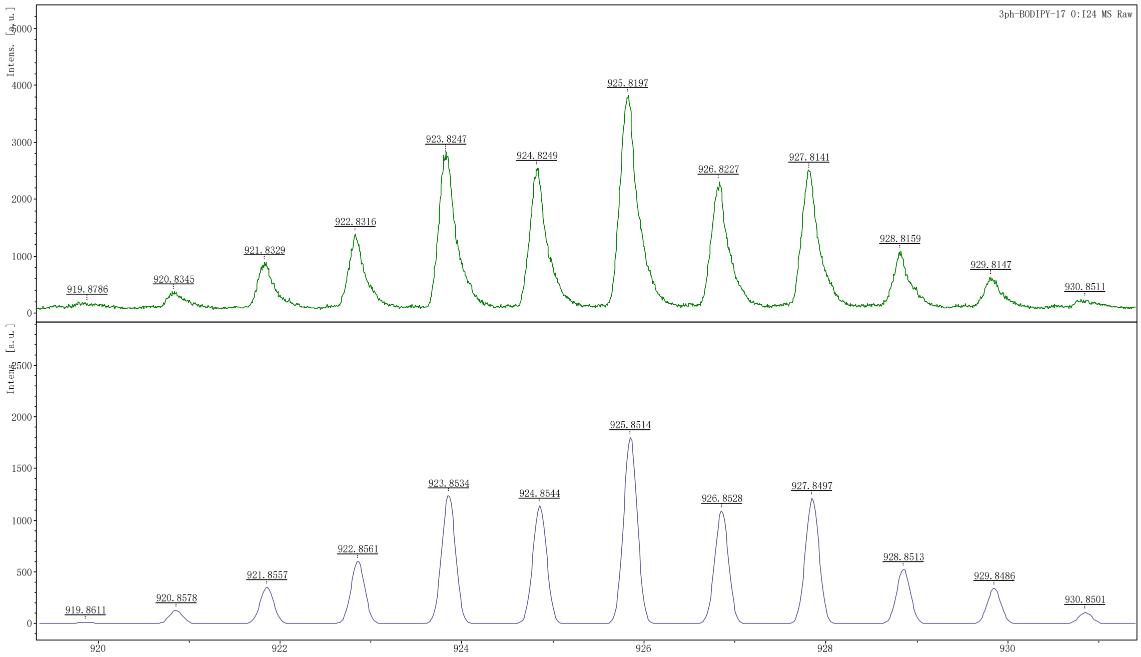

Supplementary Figure 45. MALDI-TOF-MS spectrum of **4d**.

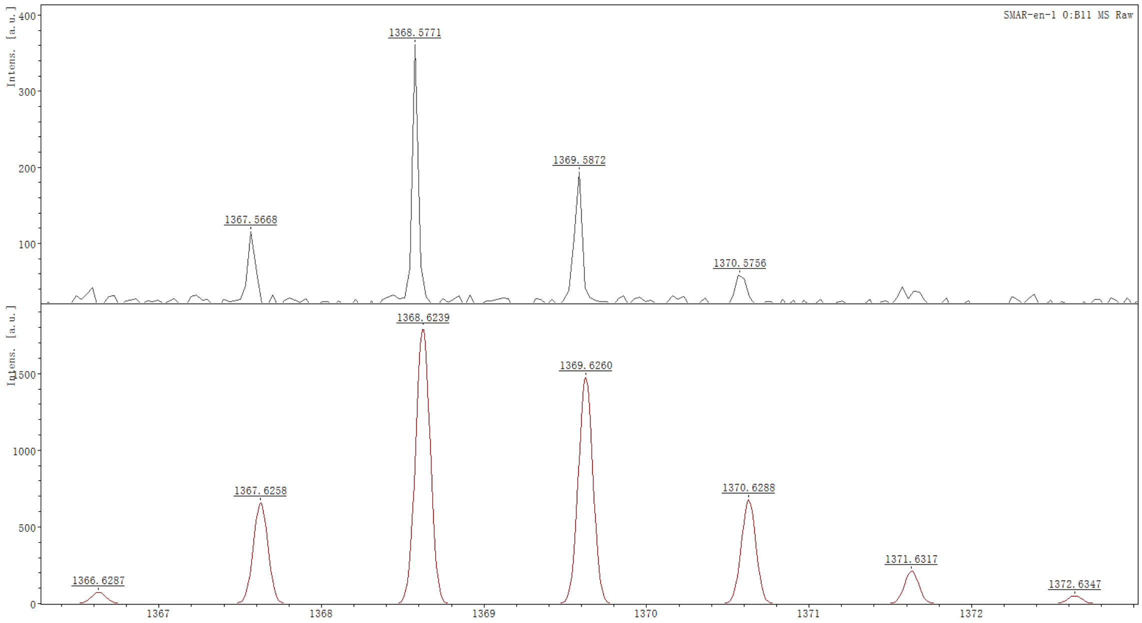

Supplementary Figure 46. MALDI-TOF-MS spectrum of **6a**.

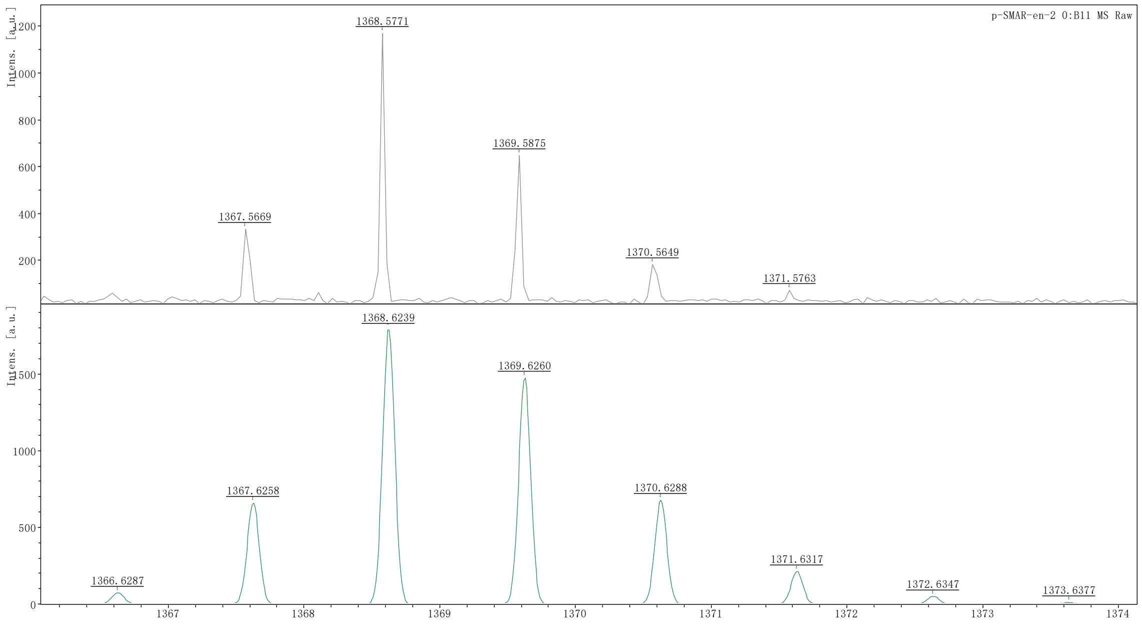

Supplementary Figure 47. MALDI-TOF-MS spectrum of **6b**.

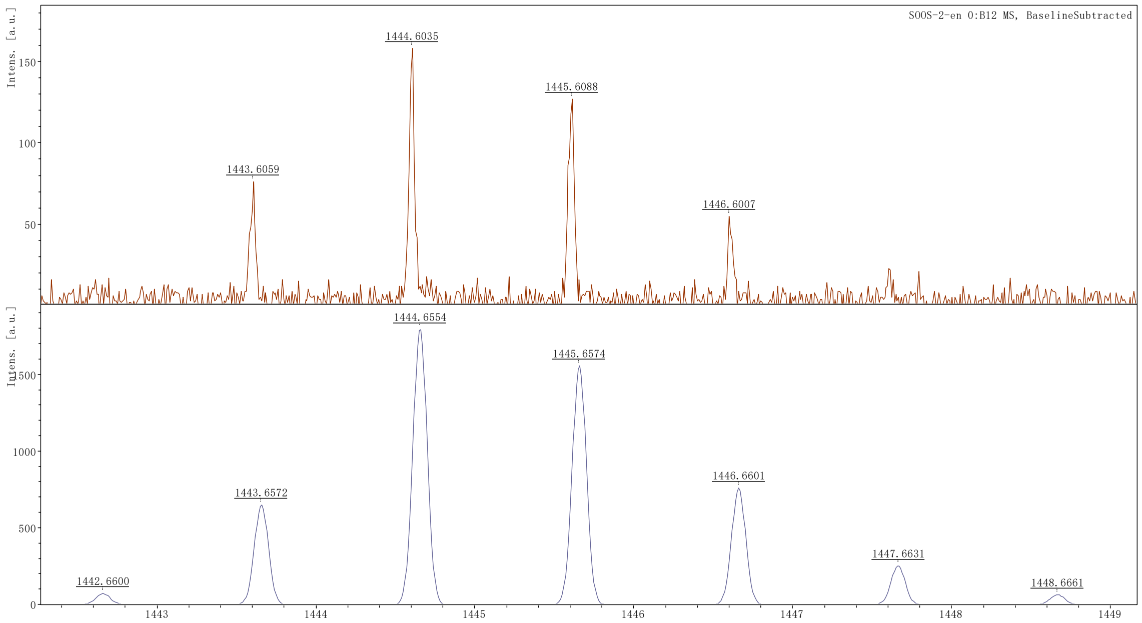

Supplementary Figure 48. MALDI-TOF-MS spectrum of **6c**.

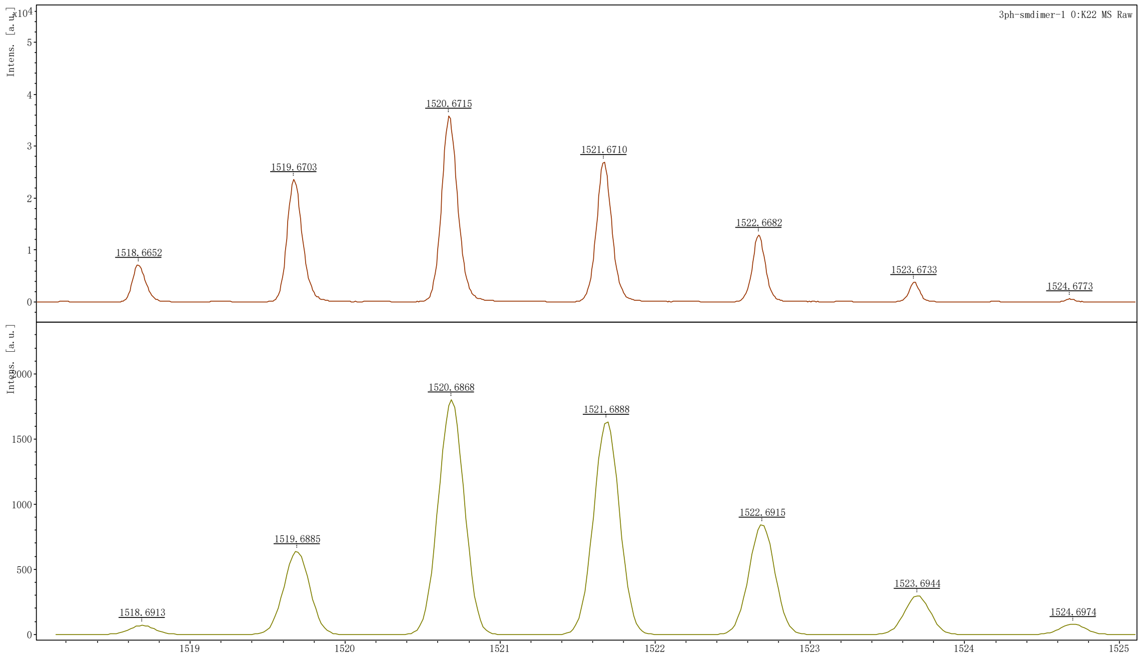

Supplementary Figure 49. MALDI-TOF-MS spectrum of **6d**.

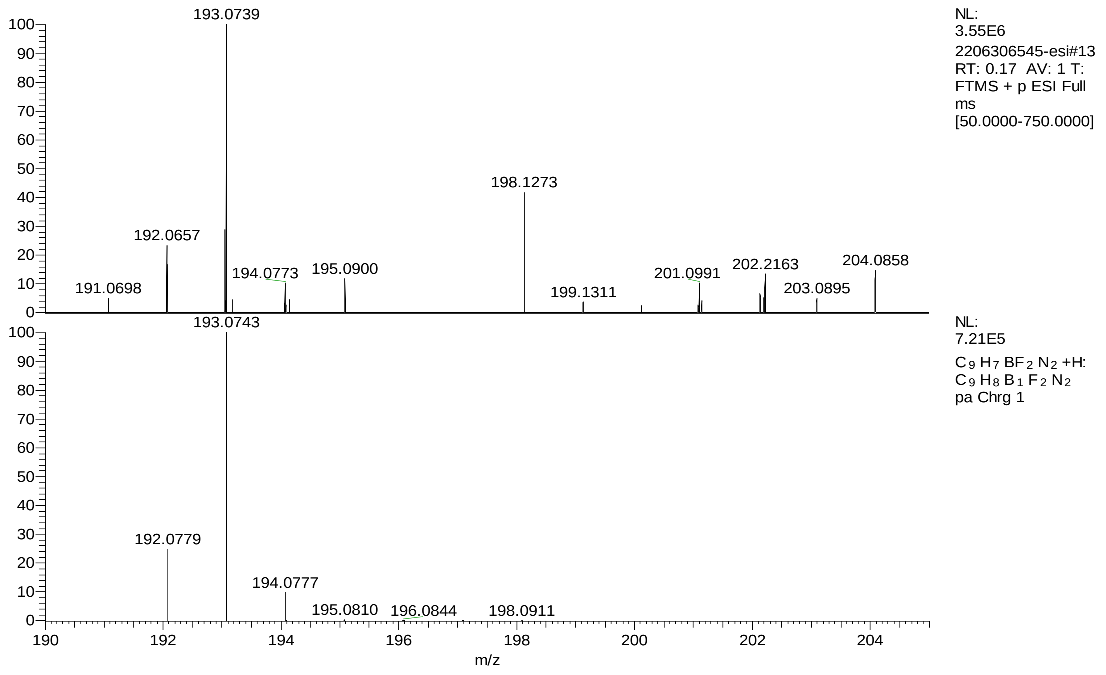

Supplementary Figure 50. ESI-MS spectrum of **9**.

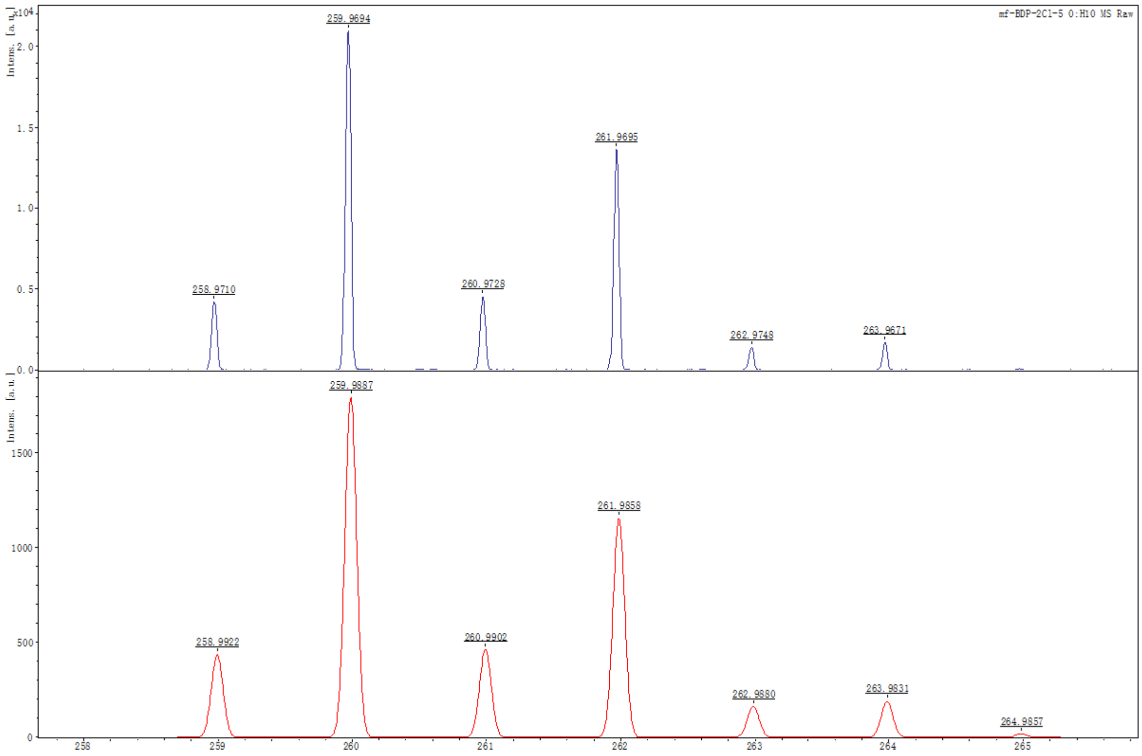

Supplementary Figure 51. MALDI-TOF-MS spectrum of **10**.

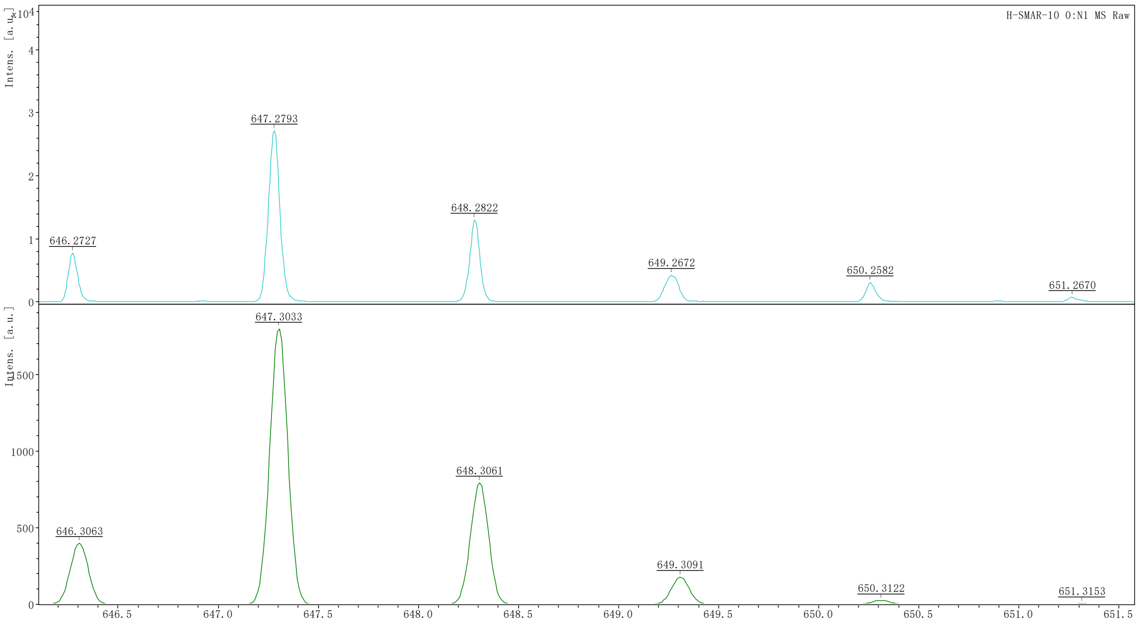

Supplementary Figure 52. MALDI-TOF-MS spectrum of **11**.

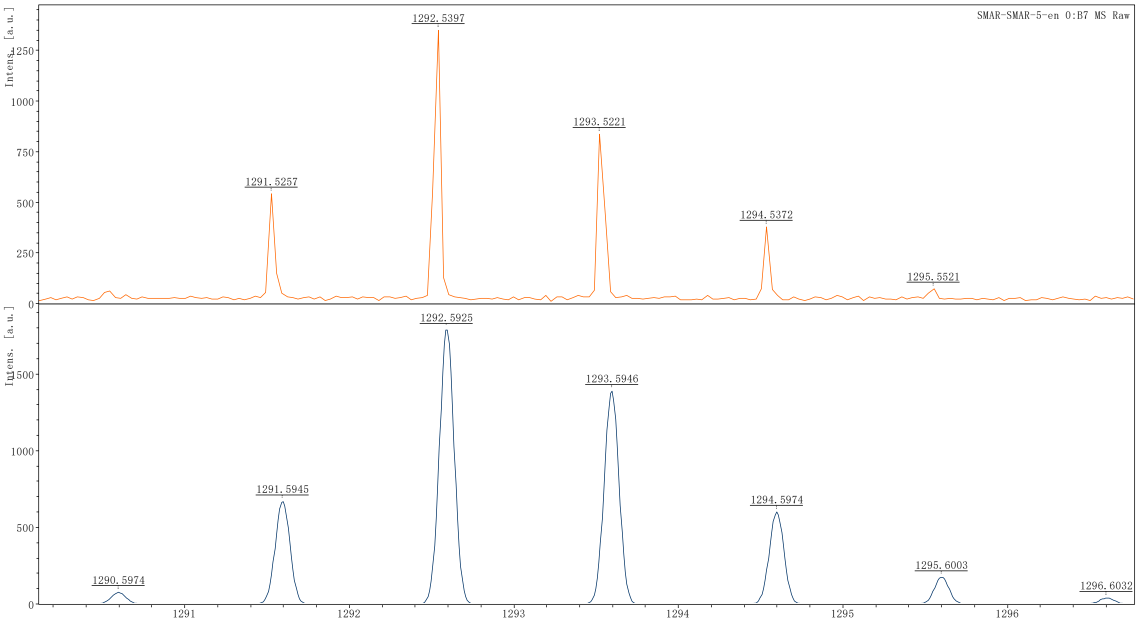

Supplementary Figure 53. MALDI-TOF-MS spectrum of **12**.

6. TD-DFT Calculation

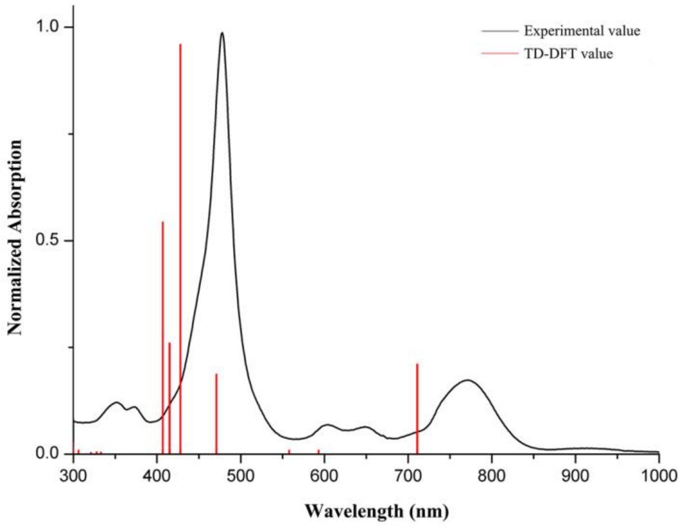

Wavelength (nm)	Oscillator Strengths	Major Transitions
711	0.6312	HOMO-3→LUMO+3 (2%); HOMO-2→LUMO+2 (4%); HOMO→LUMO (93%)
471	0.5643	HOMO-3→LUMO+3 (23%); HOMO-2→LUMO+2 (71%); HOMO-1→LUMO+1 (3%)
428	2.7471	HOMO-3→LUMO+3 (67%); HOMO-2→LUMO+2 (12%); HOMO-1→LUMO+1 (12%); HOMO→LUMO (5%)
415	0.7699	HOMO-3→LUMO (24%); HOMO-2→LUMO+1 (21%); HOMO-1→LUMO+2 (33%); HOMO→LUMO+3 (21%)
407	1.5710	HOMO-3→LUMO+1 (31%); HOMO-2→LUMO (16%); HOMO-1→LUMO-3 (33%); HOMO→LUMO+2 (19%)
300	0.0753	HOMO-14→LUMO+3 (7%); HOMO-13→LUMO+2 (14%); HOMO-1→LUMO+8 (14%); HOMO→LUMO+9 (48%)

Supplementary Figure 54. Calculated vertical transitions and major transitions of **12** calculated by TD-DFT using B3LYP employing the 6-31G(d) basis set.

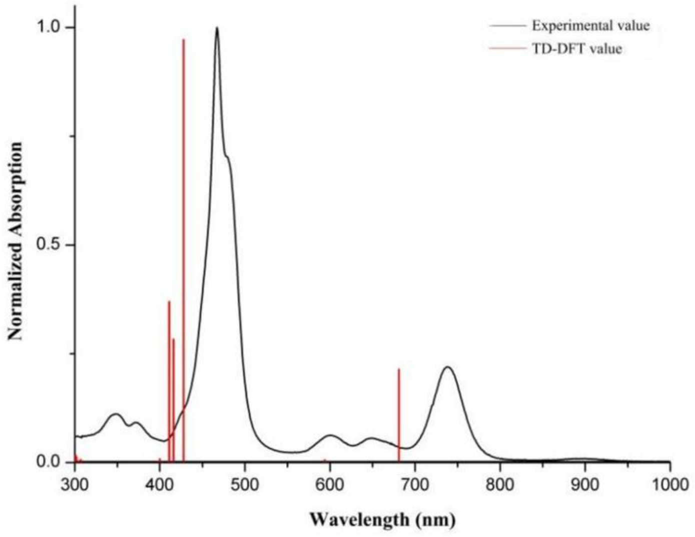

Wavelength (nm)	Oscillator Strengths	Major Transitions
681	0.6312	HOMO→LUMO (90%); HOMO-2→LUMO+2 (4%);
		HOMO-3 → LUMO+3 (3%)
428	0.5643	HOMO-1→ LUMO (2%); HOMO-3→LUMO+3 (32%);
		HOMO-2 → LUMO+2 (40%); HOMO-1 → LUMO-1 (14%);
		HOMO→LUMO (6%); HOMO→LUMO-4 (2%)
416	2.7471	HOMO-3→LUMO+1 (5%); HOMO-2→LUMO (19%);
		HOMO-2 → LUMO-1 (23%); HOMO-1 → LUMO+2 (24%);
		HOMO-1 → LUMO+3 (3%); - HOMO→LUMO+2 (25%)
411	0.7699	HOMO-3 → LUMO (18%); HOMO-3 → LUMO+l (24%);
		HOMO-2→LUMO+1 (5%); HOMO-1 → LUMO+2 (3%);
		HOMO-1 → LUNIO+3 (30%); HOMO→LUMO+3 (19%)

Supplementary Figure 55. Calculated vertical transitions and major transitions of **6b** calculated by TD-DFT using B3LYP employing the 6-31G(d) basis set.

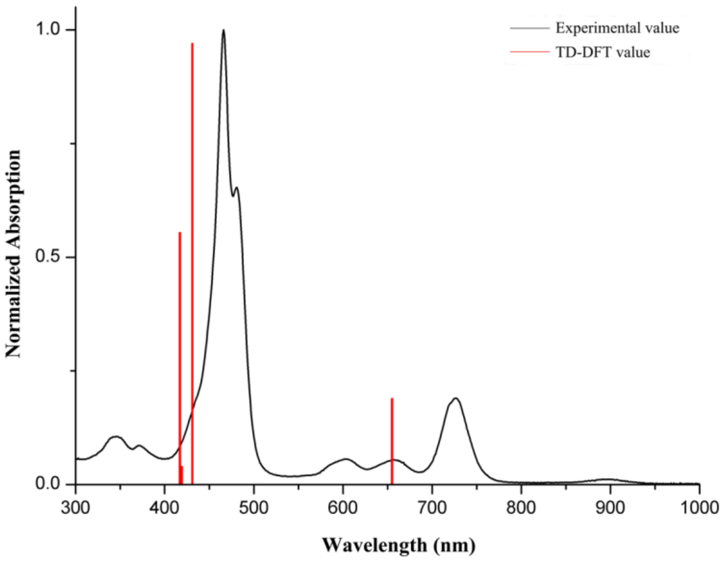

Wavelength (nm)	Oscillator Strengths	Major Transitions
655	0.7191	HOMO-3→LUMO+3 (7%); HOMO-2→LUMO+2 (7%); HOMO-1→LUMO+1 (6%); HOMO→LUMO (79%)
432	3.6960	HOMO-4→LUMO (5%); HOMO-3→LUMO+3 (34%); HOMO-2→LUMO+2 (32%); HOMO-1→LUMO+1 (11%); HOMO→LUMO (6%); HOMO→LUMO+4 (8%)
419	0.1494	HOMO-3→LUMO (20%); HOMO-2→LUMO+1 (27%); HOMO-1→LUMO+3 (28%); HOMO→LUMO+3 (24%)
417	2.1108	HOMO-3→LUMO+1 (27%); HOMO-2→LUMO (20%); HOMO-1→LUMO+3 (27%); HOMO→LUMO+2 (24%)

Supplementary Figure 56. Calculated vertical transitions and major transitions of **6c** calculated by TD-DFT using B3LYP employing the 6-31G(d) basis set.

7.<u>Time-Resolved Fluorescence Decay.</u>

Supplementary Note 1

A time-correlated single-photon-counting (TCSPC) system was used for measurements of spontaneous fluorescence decay. As an excitation light source, we used a mode-locked Ti:sapphire laser (Spectra Physics, MaiTai BB) which provides ultrashort pulse (80 fs at full width half maximum, fwhm) with high repetition rate (80 MHz). This high repetition rate slows down to 800 kHz by using homemade pulse-picker. The pulse-picked output pulse was frequency doubled by a 1-mm-thick BBO crystal (type-I, θ = 29.2°, EKSMA). The fluorescence was collected by a microchannel plate photomultiplier (MCP-PMT, Hamamatsu, R3809U-51) with a thermoelectric cooler (Hamamatsu, C4878) connected to a TCSPC board (Becker & Hickel SPC-130). The overall instrumental response function was about 25 ps (FWHM). A vertically polarized pump pulse by a Glan-laser polarizer was irradiated to samples, and a sheet polarizer set at an angle complementary to the magic angle (54.7°), was placed in the fluorescence collection path to obtain polarization-independent fluorescence decays.

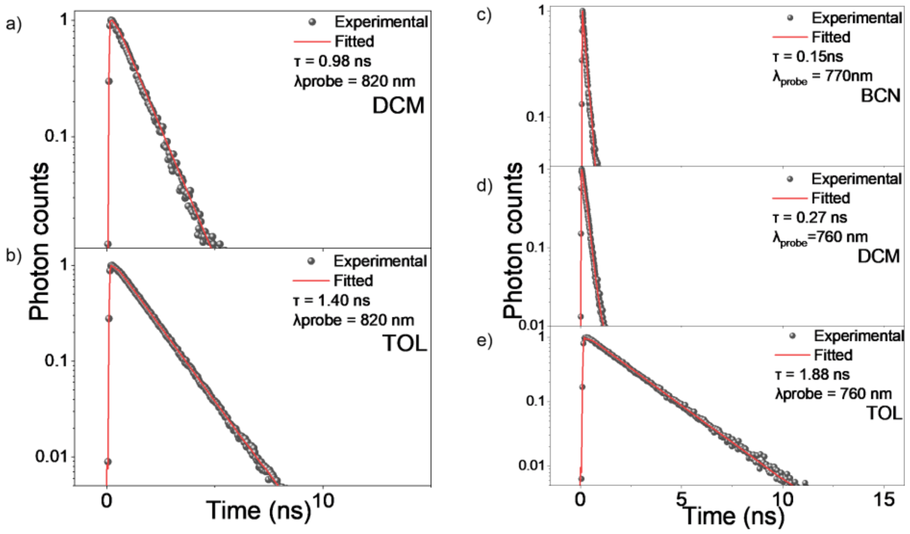

Supplementary Figure 57. Fluorescence lifetimes of **12** and **6b** were obtained from TCSPC measurement. a) **12** in dichloromethane, b) **12** in toluene, c) **6b** in benzonitrile, d) **6b** in dichloromethane, and e) **6b** in toluene.

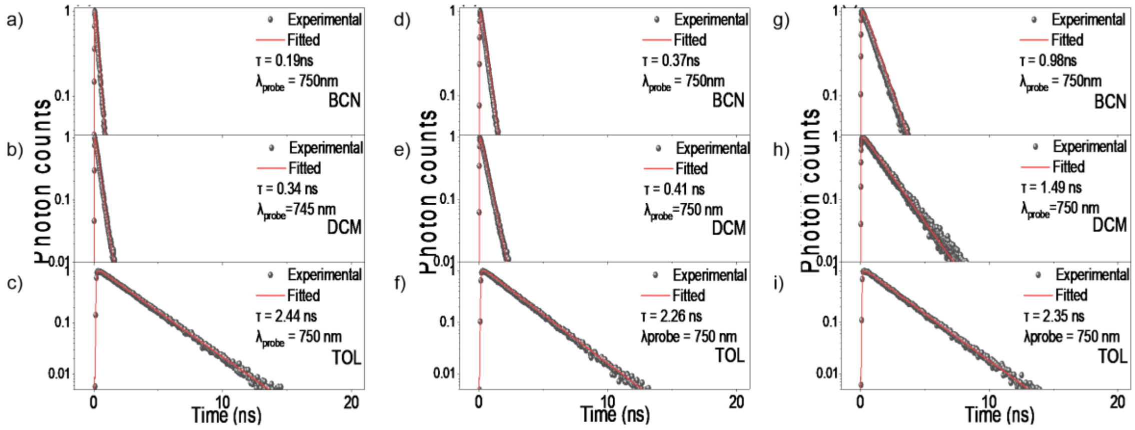

Supplementary Figure 58. Fluorescence lifetimes of **6a**, **6c** and **6d** were obtained from TCSPC measurement. a) **6a** in benzonitrile, b) **6a** in dichloromethane, c) **6a** in toluene, d) **6c** in benzonitrile, e) **6c** in dichloromethane, f) **6c** in toluene, g) **6d** in benzonitrile, h) **6d** in dichloromethane, and i) **6d** in toluene.

8.<u>Femtosecond Transient Absorption Spectra and Decay Profiles.</u>

Supplementary Note 2

A femtosecond time-resolved transient absorption (fs-TA) spectrometer consists of Optical Parametric Amplifiers (Palitra, Quantronix) pumped by a Ti:sapphire regenerative amplifier system (Integra-C, Quantronix) operating at 1 kHz repetition rate and an optical detection system. The generated OPA pulses had a pulse width of ~ 100 fs and an average power of 6 mW in the range 280–2700 nm which were used as pump pulses. White light continuum (WLC) probe pulses were generated using a sapphire window (3 mm thick) by focusing of small portion of the fundamental 800 nm pulses which was picked off by a quartz plate before entering the OPA. The time delay between pump and probe beams was carefully controlled by making the pump beam travel along a variable optical delay (ILS250, Newport). Intensities of the spectrally dispersed WLC probe pulses are monitored by a High Speed spectrometer (Ultrafast Systems). To obtain the time-resolved transient absorption difference signal (ΔA) at a specific time, the pump pulses were chopped at 500 Hz and absorption spectra intensities were saved alternately with or without pump pulse. Typically, 4000 pulses excite samples to obtain the fs-TA spectra at each delay time. The polarization angle between pump and probe beam was set at the magic angle (54.7°) using a Glan-laser polarizer with a halfwave retarder in order to prevent polarization-dependent signals. Cross-correlation FWHM in pump-probe experiments was less than 200 fs and chirp of WLC probe pulses was measured to be 800 fs in the 400–800 nm region. To minimize chirp, all reflection optics in the probe beam path and a quartz cell of the 2 mm path length were used. After fs-TA experiments, the absorption spectra of all compounds were carefully examined to detect if there were artifacts due to degradation and photo-oxidation of samples. The three-dimensional data sets of ΔA versus time and wavelength were subjected to singular value decomposition and global fitting to obtain the kinetic time constants and their associated spectra using Surface Xplorer software (Ultrafast Systems).

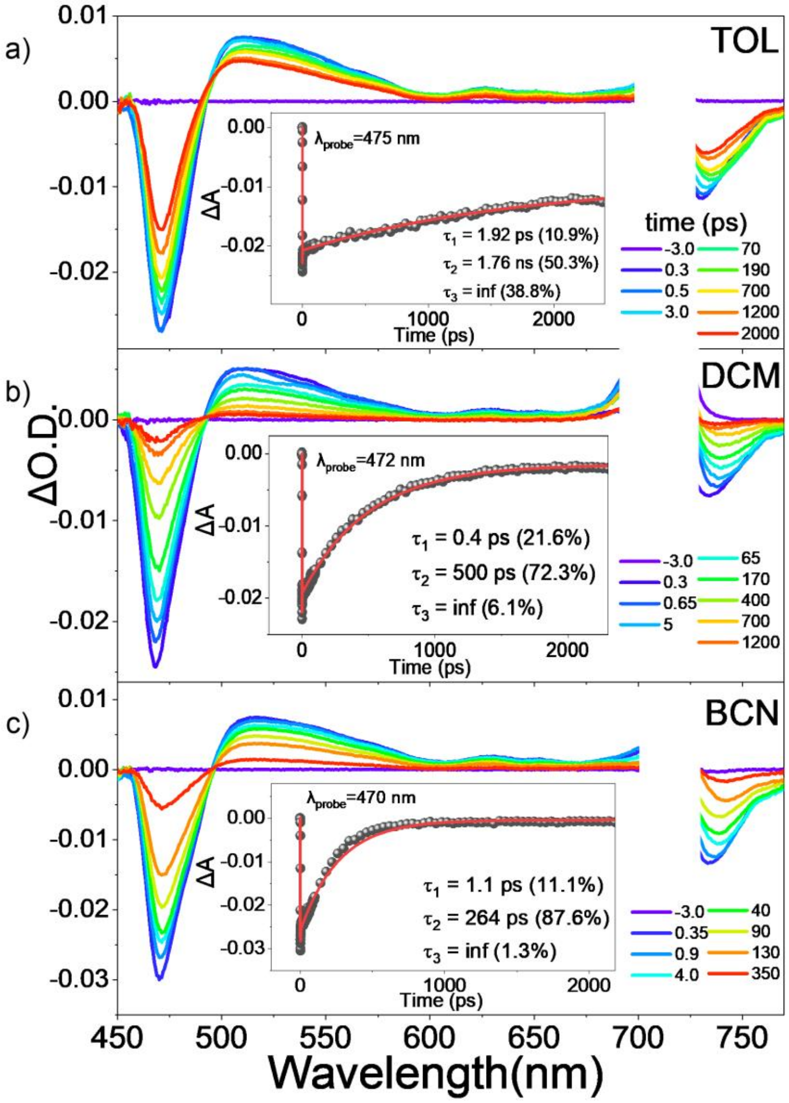

Supplementary Figure 59. Femtosecond–Transient absorption (fs-TA) spectra and decay profiles of **6a** in toluene a), in dichloromethane b), in benzonitrile c). The samples were pumped at 710 nm with probing visible region to the near-IR region (450-780 nm).

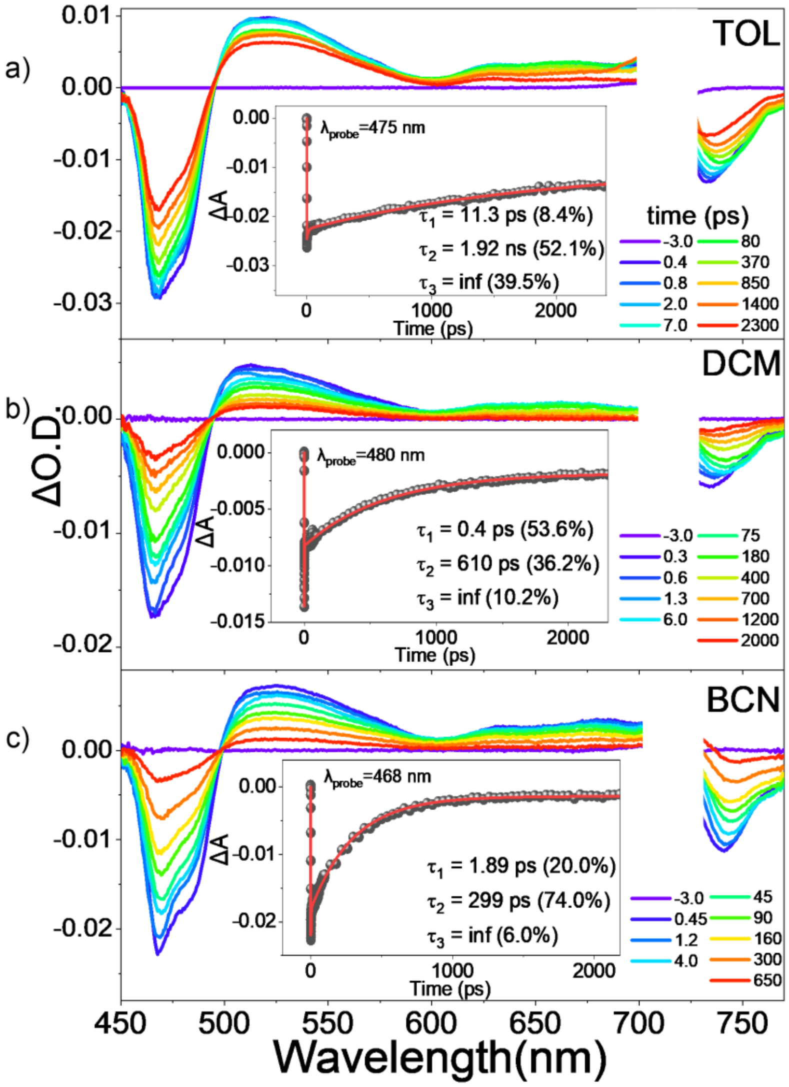

Supplementary Figure 60. Femtosecond–Transient absorption (fs-TA) spectra and decay profiles of **6c** in toluene a), in dichloromethane b), in benzonitrile c). The samples were pumped at 710 nm with probing visible region to the near-IR region (450-780 nm).

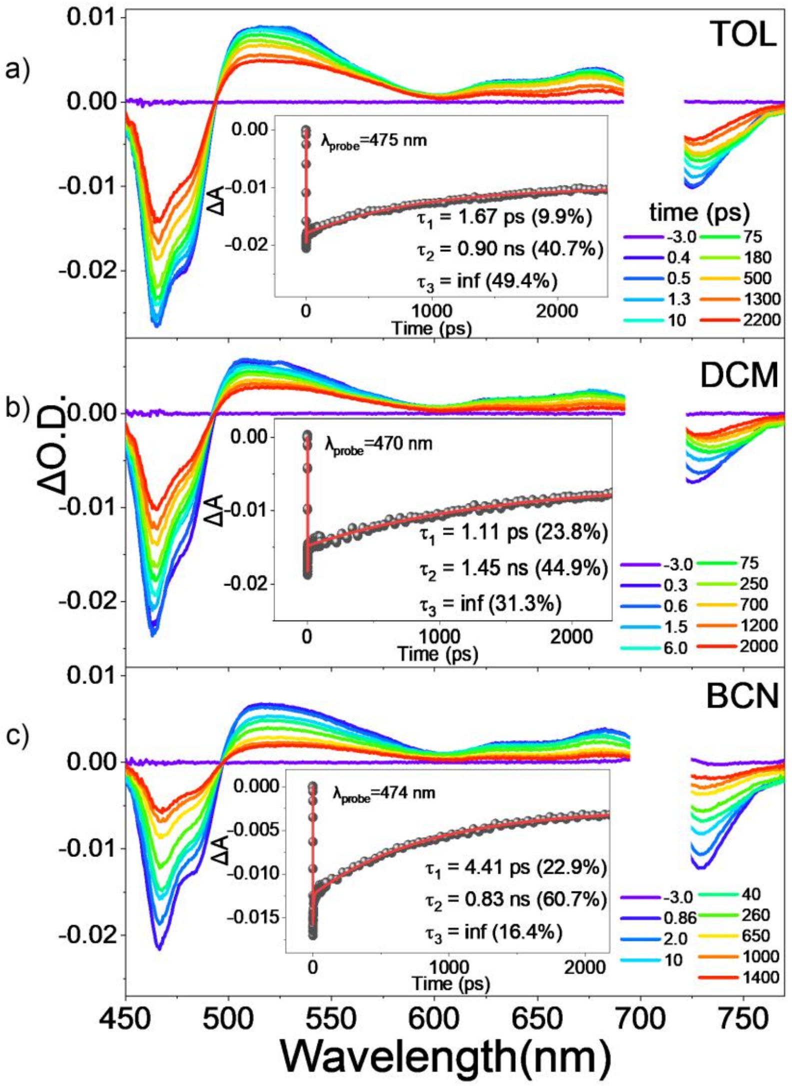

Supplementary Figure 61. Femtosecond–Transient absorption (fs-TA) spectra and decay profiles of **6d** in toluene a), in dichloromethane b), in benzonitrile c). The samples were pumped at 710 nm with probing visible region to the near-IR region (450-780 nm).
