# Supplementary material for: Aromatic-bridged and meso-meso-linked BF2-smaragdyrin dimers exhibit fast decays in polar solvents by symmetry-breaking charge transfer
Source: Commun Chem. 2023 Feb 9;6:25. doi: 10.1038/s42004-023-00822-8 (PMC9911704; doi:10.1038/s42004-023-00822-8)
Supplement: Supplementary file 3 — Description of Additional Supplementary Files [file 42004_2023_822_MOESM3_ESM.pdf]

Description of Additional Supplementary Files

File name: Supplementary Data 1

Description: cif file of **6b**

File name: Supplementary Data 2

Description: check cif file of **6b**

File name: Supplementary Data 3

Description: cif file of **6c**

File name: Supplementary Data 4

Description: check cif file of **6c**

File name: Supplementary Data 5

Description: cif file of **10**

File name: Supplementary Data 6

Description: check cif file of **10**

File name: Supplementary Data 7

Description: cif file of **12**

File name: Supplementary Data 8

Description: check cif file of **12**

File name: Supplementary Data 9

Description: optimized atomic coordinates
