# Supplementary material for: Aromatic-bridged and meso-meso-linked BF2-smaragdyrin dimers exhibit fast decays in polar solvents by symmetry-breaking charge transfer
Source: Commun Chem. 2023 Feb 9;6:25. doi: 10.1038/s42004-023-00822-8 (PMC9911704; doi:10.1038/s42004-023-00822-8)

checkCIF/PLATON report

Structure factors have been supplied for datablock(s) exp_991_sq_sq

THIS REPORT IS FOR GUIDANCE ONLY. IF USED AS PART OF A REVIEW PROCEDURE FOR PUBLICATION, IT SHOULD NOT REPLACE THE EXPERTISE OF AN EXPERIENCED CRYSTALLOGRAPHIC REFEREE.

No syntax errors found. CIF dictionary Interpreting this report

Datablock: exp_991_sq_sq

```
Bond precision:   C-C = 0.0057 A          Wavelength=1.54184

Cell:             a=21.0388(7)     b=31.2465(10)      c=29.3757(10)
                  alpha=90         beta=105.226(4)    gamma=90
Temperature:      97 K
```

	Calculated	Reported
Volume	18633.4(11)	18633.4(11)
Space group	I 2/a	I 1 2/a 1
Hall group	-I 2ya	-I 2ya
Moiety formula	C88 H74 B2 F4 N10 [+ solvent]	2(C44 H37 B F2 N5)
Sum formula	C88 H74 B2 F4 N10 [+ solvent]	C88 H74 B2 F4 N10
Mr	1369.19	1369.19
Dx,g cm-3	0.976	0.976
Z	8	8
Mu (mm-1)	0.505	0.505
F000	5744.0	5744.0
F000'	5760.64	
h,k,lmax	25,37,34	25,37,34
Nref	16469	16448
Tmin,Tmax	0.970,0.975	0.273,1.000
Tmin'	0.904	

```
Correction method= # Reported T Limits: Tmin=0.273 Tmax=1.000
AbsCorr = MULTI-SCAN

Data completeness= 0.999            Theta(max)= 66.601
```

R(reflections)= 0.0772(9895) wR2(reflections)= 0.2397(16448)

S = 1.063 Npar= 972

The following ALERTS were generated. Each ALERT has the format
test-name_ALERT_alert-type_alert-level.
Click on the hyperlinks for more details of the test.

Alert level B

```
PLAT416_ALERT_2_B Short Intra D-H..H-D       H1        ..H2         .         1.80 Ang.
                                                 x,y,z  =     1_555 Check
PLAT416_ALERT_2_B Short Intra D-H..H-D       H1        ..H2         .         1.80 Ang.
                                           3/2-x,y,1-z  =     2_656 Check
PLAT416_ALERT_2_B Short Intra D-H..H-D       H5        ..H6         .         1.81 Ang.
                                                 x,y,z  =     1_555 Check
PLAT416_ALERT_2_B Short Intra D-H..H-D       H7        ..H11        .         1.87 Ang.
                                                 x,y,z  =     1_555 Check
```

Alert level C

```
PLAT213_ALERT_2_C Atom C37            has ADP max/min Ratio .....         3.1 prolat
PLAT213_ALERT_2_C Atom C46            has ADP max/min Ratio .....         3.2 prolat
PLAT220_ALERT_2_C NonSolvent   Resd 1  C   Ueq(max)/Ueq(min) Range        3.2 Ratio
PLAT241_ALERT_2_C High   'MainMol' Ueq as Compared to Neighbors of         C5 Check
PLAT241_ALERT_2_C High   'MainMol' Ueq as Compared to Neighbors of         C6 Check
PLAT241_ALERT_2_C High   'MainMol' Ueq as Compared to Neighbors of        C53 Check
PLAT250_ALERT_2_C Large U3/U1 Ratio for Average U(i,j) Tensor ....        2.1 Note
PLAT250_ALERT_2_C Large U3/U1 Ratio for Average U(i,j) Tensor ....        2.5 Note
PLAT340_ALERT_3_C Low Bond Precision on  C-C Bonds ..............     0.00567 Ang.
PLAT416_ALERT_2_C Short Intra D-H..H-D       H7        ..H8         .         1.91 Ang.
                                                 x,y,z  =     1_555 Check
PLAT906_ALERT_3_C Large K Value in the Analysis of Variance ......      2.090 Check
PLAT911_ALERT_3_C Missing FCF Refl Between Thmin & STh/L=    0.595         17 Report
```

Alert level G

```
PLAT003_ALERT_2_G Number of Uiso or Uij Restrained non-H Atoms ...        110 Report
PLAT007_ALERT_5_G Number of Unrefined Donor-H Atoms ..............          7 Report
PLAT042_ALERT_1_G Calc. and Reported MoietyFormula Strings  Differ     Please Check
PLAT072_ALERT_2_G SHELXL First  Parameter in WGHT  Unusually Large       0.11 Report
PLAT177_ALERT_4_G The CIF-Embedded .res File Contains DELU Records          1 Report
PLAT186_ALERT_4_G The CIF-Embedded .res File Contains ISOR Records          2 Report
PLAT301_ALERT_3_G Main Residue  Disorder ..............(Resd  1  )         4% Note
PLAT606_ALERT_4_G Solvent Accessible VOID(S) in Structure ........          ! Info
PLAT720_ALERT_4_G Number of Unusual/Non-Standard Labels ..........          4 Note
PLAT860_ALERT_3_G Number of Least-Squares Restraints .............        391 Note
PLAT869_ALERT_4_G ALERTS Related to the Use of SQUEEZE  Suppressed          ! Info
PLAT883_ALERT_1_G No Info/Value for _atom_sites_solution_primary .     Please Do !
PLAT909_ALERT_3_G Percentage of I>2sig(I) Data at Theta(Max) Still        36% Note
PLAT910_ALERT_3_G Missing # of FCF Reflection(s) Below Theta(Min).          4 Note
PLAT913_ALERT_3_G Missing # of Very Strong Reflections in FCF ....          2 Note
PLAT933_ALERT_2_G Number of HKL-OMIT Records in Embedded .res File         14 Note
PLAT941_ALERT_3_G Average HKL Measurement Multiplicity ...........        3.4 Low
PLAT978_ALERT_2_G Number C-C Bonds with Positive Residual Density.          1 Info
```

```
  0 ALERT level A = Most likely a serious problem - resolve or explain
  4 ALERT level B = A potentially serious problem, consider carefully
 12 ALERT level C = Check. Ensure it is not caused by an omission or oversight
 18 ALERT level G = General information/check it is not something unexpected

  2 ALERT type 1 CIF construction/syntax error, inconsistent or missing data
 17 ALERT type 2 Indicator that the structure model may be wrong or deficient
  9 ALERT type 3 Indicator that the structure quality may be low
  5 ALERT type 4 Improvement, methodology, query or suggestion
  1 ALERT type 5 Informative message, check
```

It is advisable to attempt to resolve as many as possible of the alerts in all categories. Often the minor alerts point to easily fixed oversights, errors and omissions in your CIF or refinement strategy, so attention to these fine details can be worthwhile. In order to resolve some of the more serious problems it may be necessary to carry out additional measurements or structure refinements. However, the purpose of your study may justify the reported deviations and the more serious of these should normally be commented upon in the discussion or experimental section of a paper or in the "special_details" fields of the CIF. checkCIF was carefully designed to identify outliers and unusual parameters, but every test has its limitations and alerts that are not important in a particular case may appear. Conversely, the absence of alerts does not guarantee there are no aspects of the results needing attention. It is up to the individual to critically assess their own results and, if necessary, seek expert advice.

Publication of your CIF in IUCr journals

A basic structural check has been run on your CIF. These basic checks will be run on all CIFs submitted for publication in IUCr journals (*Acta Crystallographica*, *Journal of Applied Crystallography*, *Journal of Synchrotron Radiation*); however, if you intend to submit to *Acta Crystallographica Section C* or *E* or *IUCrData*, you should make sure that full publication checks are run on the final version of your CIF prior to submission.

Publication of your CIF in other journals

Please refer to the *Notes for Authors* of the relevant journal for any special instructions relating to CIF submission.

PLATON version of 18/05/2022; check.def file version of 17/05/2022

Datablock exp_991_sq_sq - ellipsoid plot

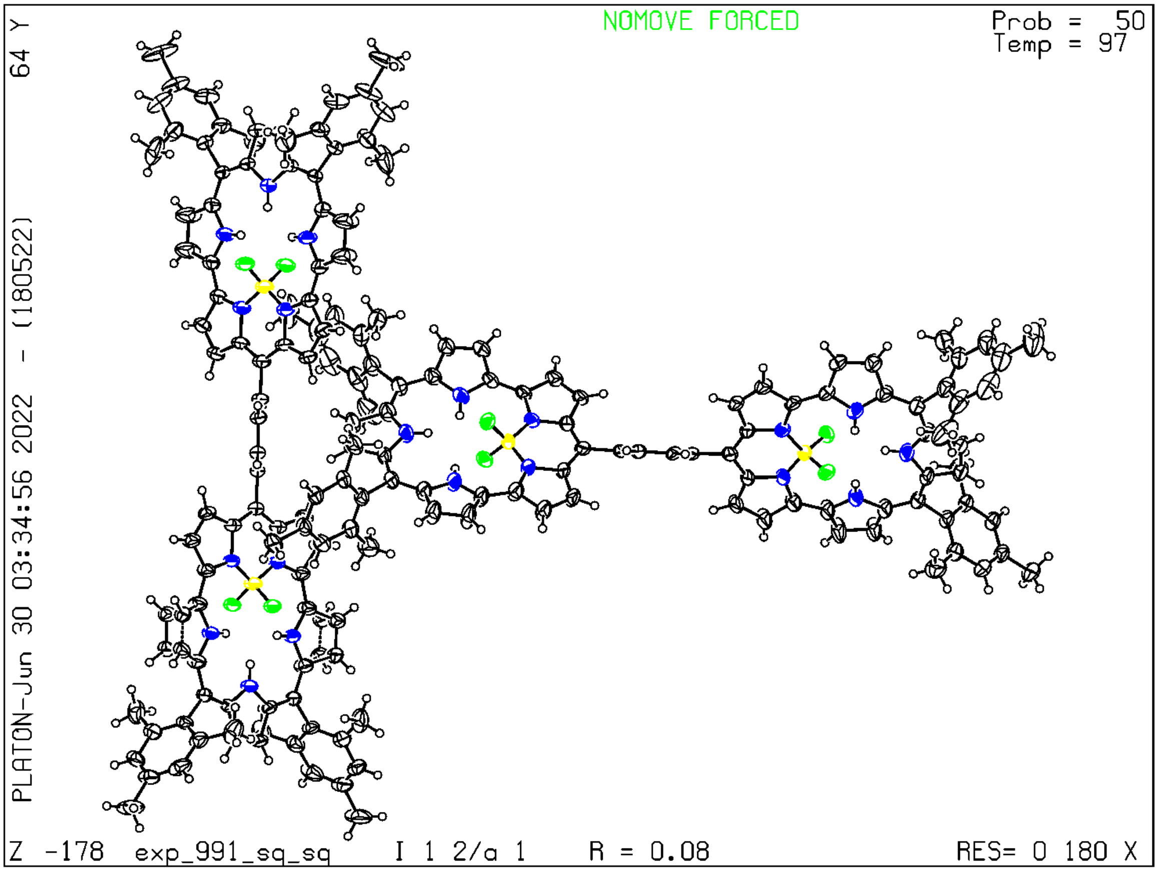

Supplement: Supplementary file 5 — Supplementary Data 2 [file 42004_2023_822_MOESM5_ESM.pdf]
