# Supplementary material for: Aromatic-bridged and meso-meso-linked BF2-smaragdyrin dimers exhibit fast decays in polar solvents by symmetry-breaking charge transfer
Source: Commun Chem. 2023 Feb 9;6:25. doi: 10.1038/s42004-023-00822-8 (PMC9911704; doi:10.1038/s42004-023-00822-8)

checkCIF/PLATON report

Structure factors have been supplied for datablock(s) exp_996

THIS REPORT IS FOR GUIDANCE ONLY. IF USED AS PART OF A REVIEW PROCEDURE FOR PUBLICATION, IT SHOULD NOT REPLACE THE EXPERTISE OF AN EXPERIENCED CRYSTALLOGRAPHIC REFEREE.

No syntax errors found. CIF dictionary Interpreting this report

Datablock: exp_996

Bond precision:	C-C = 0.0030 A	Wavelength=1.54184	
Cell:	a=7.1012(10)	b=10.1746(14)	c=13.888(2)
	alpha=90	beta=96.878(16)	gamma=90
Temperature:	99 K		

	Calculated	Reported
Volume	996.2(2)	996.2(3)
Space group	P 21/c	P 1 21/c 1
Hall group	-P 2ybc	-P 2ybc
Moiety formula	C9 H5 B Cl2 F2 N2	C9 H5 B Cl2 F2 N2
Sum formula	C9 H5 B Cl2 F2 N2	C9 H5 B Cl2 F2 N2
Mr	260.86	260.86
Dx,g cm-3	1.739	1.739
Z	4	4
Mu (mm-1)	5.893	5.893
F000	520.0	520.0
F000'	524.39	
h,k,lmax	8,12,16	8,12,16
Nref	1759	1750
Tmin,Tmax	0.518,0.555	0.306,1.000
Tmin'	0.163	

Correction method= # Reported T Limits: Tmin=0.306 Tmax=1.000
AbsCorr = MULTI-SCAN

Data completeness= 0.995 Theta(max)= 66.592

R(reflections)= 0.0442(1672) wR2(reflections)= 0.1222(1750)

S = 1.052 Npar= 145

The following ALERTS were generated. Each ALERT has the format
test-name_ALERT_alert-type_alert-level.
Click on the hyperlinks for more details of the test.

Alert level C

PLAT911_ALERT_3_C Missing FCF Refl Between Thmin & STh/L= 0.595 10 Report

Alert level G

PLAT883_ALERT_1_G No Info/Value for _atom_sites_solution_primary . Please Do !
PLAT909_ALERT_3_G Percentage of I>2sig(I) Data at Theta(Max) Still 93% Note
PLAT941_ALERT_3_G Average HKL Measurement Multiplicity .... 1.8 Low
PLAT978_ALERT_2_G Number C-C Bonds with Positive Residual Density. 2 Info

0 **ALERT level A** = Most likely a serious problem - resolve or explain
0 **ALERT level B** = A potentially serious problem, consider carefully
1 **ALERT level C** = Check. Ensure it is not caused by an omission or oversight
4 **ALERT level G** = General information/check it is not something unexpected

1 ALERT type 1 CIF construction/syntax error, inconsistent or missing data
1 ALERT type 2 Indicator that the structure model may be wrong or deficient
3 ALERT type 3 Indicator that the structure quality may be low
0 ALERT type 4 Improvement, methodology, query or suggestion
0 ALERT type 5 Informative message, check

It is advisable to attempt to resolve as many as possible of the alerts in all categories. Often the minor alerts point to easily fixed oversights, errors and omissions in your CIF or refinement strategy, so attention to these fine details can be worthwhile. In order to resolve some of the more serious problems it may be necessary to carry out additional measurements or structure refinements. However, the purpose of your study may justify the reported deviations and the more serious of these should normally be commented upon in the discussion or experimental section of a paper or in the "special_details" fields of the CIF. checkCIF was carefully designed to identify outliers and unusual parameters, but every test has its limitations and alerts that are not important in a particular case may appear. Conversely, the absence of alerts does not guarantee there are no aspects of the results needing attention. It is up to the individual to critically assess their own results and, if necessary, seek expert advice.

Publication of your CIF in IUCr journals

A basic structural check has been run on your CIF. These basic checks will be run on all CIFs submitted for publication in IUCr journals (*Acta Crystallographica*, *Journal of Applied Crystallography*, *Journal of Synchrotron Radiation*); however, if you intend to submit to *Acta Crystallographica Section C* or *E* or *IUCrData*, you should make sure that full publication checks are run on the final version of your CIF prior to submission.

Publication of your CIF in other journals

Please refer to the *Notes for Authors* of the relevant journal for any special instructions relating to CIF submission.

PLATON version of 18/05/2022; check.def file version of 17/05/2022

Datablock exp_996 - ellipsoid plot

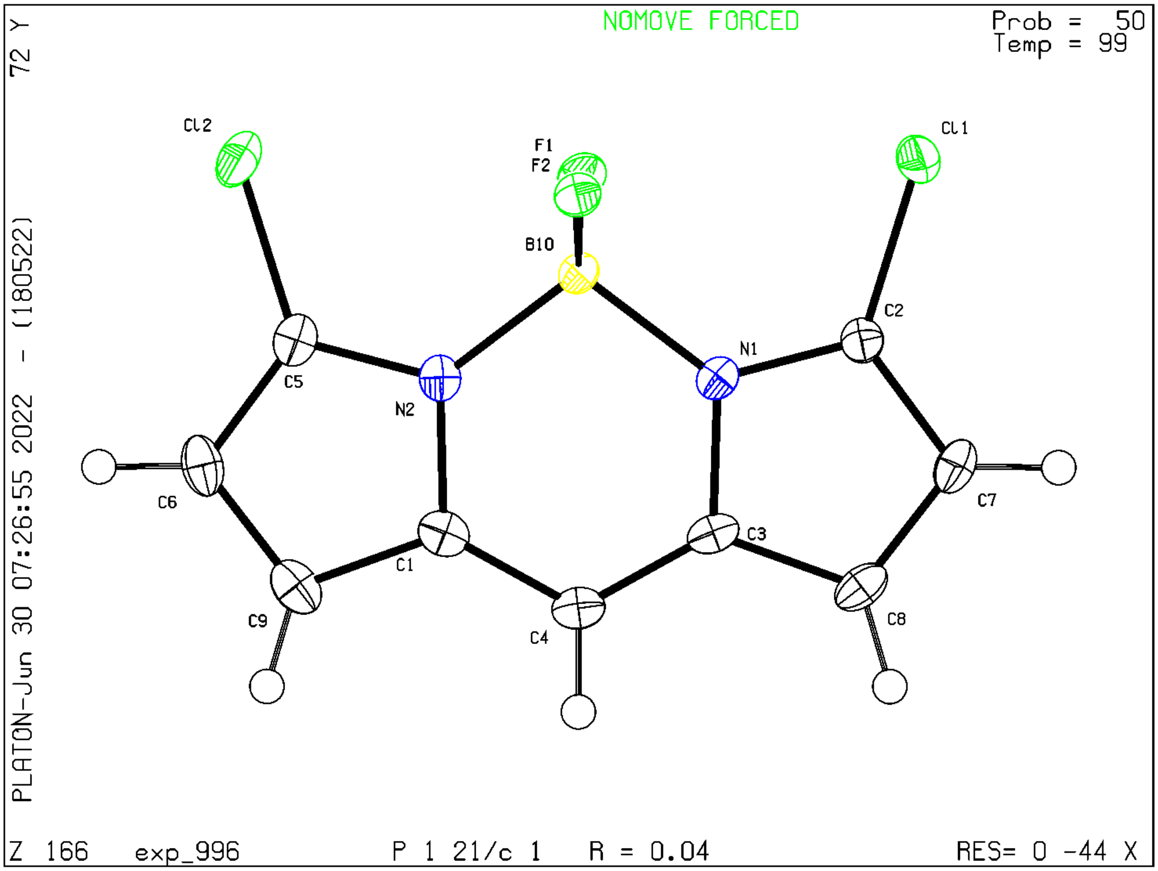

Supplement: Supplementary file 9 — Supplementary Data 6 [file 42004_2023_822_MOESM9_ESM.pdf]
