# Supplementary material for: Aromatic-bridged and meso-meso-linked BF2-smaragdyrin dimers exhibit fast decays in polar solvents by symmetry-breaking charge transfer
Source: Commun Chem. 2023 Feb 9;6:25. doi: 10.1038/s42004-023-00822-8 (PMC9911704; doi:10.1038/s42004-023-00822-8)

checkCIF/PLATON report

Structure factors have been supplied for datablock(s) exp_1516_sq_sq

THIS REPORT IS FOR GUIDANCE ONLY. IF USED AS PART OF A REVIEW PROCEDURE FOR PUBLICATION, IT SHOULD NOT REPLACE THE EXPERTISE OF AN EXPERIENCED CRYSTALLOGRAPHIC REFEREE.

No syntax errors found. CIF dictionary Interpreting this report

Datablock: exp_1516_sq_sq

Bond precision: C-C = 0.0048 A Wavelength=1.54184

Cell:	a=12.1103(3)	b=17.8722(5)	c=21.2502(6)
	alpha=104.055(2)	beta=97.125(2)	gamma=101.364(2)
Temperature:	100 K		

	Calculated	Reported
Volume	4302.2(2)	4302.2(2)
Space group	P -1	P -1
Hall group	-P 1	-P 1
Moiety formula	C82 H70 B2 F4 N10, 2.5(C2 H4 Cl2) [+ solvent]	2.5(C2 H4 Cl2), C82 H70 B2 F4 N10
Sum formula	C87 H80 B2 Cl5 F4 N10 [+ solvent]	C87 H80 B2 Cl5 F4 N10
Mr	1540.48	1540.48
Dx,g cm-3	1.189	1.189
Z	2	2
Mu (mm-1)	1.993	1.993
F000	1606.0	1606.0
F000'	1613.79	
h,k,lmax	14,21,25	14,21,25
Nref	15214	15209
Tmin,Tmax	0.787,0.961	0.788,1.000
Tmin'	0.550	

Correction method= # Reported T Limits: Tmin=0.788 Tmax=1.000
AbsCorr = MULTI-SCAN

Data completeness= 1.000 Theta(max)= 66.599

R(reflections)= 0.0605(11590)　　wR2(reflections)= 0.1563(15209)

S = 1.031　　Npar= 1090

The following ALERTS were generated. Each ALERT has the format
test-name_ALERT_alert-type_alert-level.
Click on the hyperlinks for more details of the test.

Alert level B

```
PLAT351_ALERT_3_B Long   C-H (X0.96,N1.08A)  C10       - H10H     .        1.17 Ang.
PLAT416_ALERT_2_B Short Intra D-H..H-D       H13       ..H17      .        1.89 Ang.
                                                       x,y,z  =      1_555 Check
PLAT416_ALERT_2_B Short Intra D-H..H-D       H13       ..H31      .        1.88 Ang.
                                                       x,y,z  =      1_555 Check
PLAT416_ALERT_2_B Short Intra D-H..H-D       H65       ..H71      .        1.81 Ang.
                                                       x,y,z  =      1_555 Check
```

Alert level C

```
PLAT213_ALERT_2_C Atom B50             has ADP max/min Ratio .....         3.7 oblate
PLAT220_ALERT_2_C NonSolvent   Resd 1  C   Ueq(max)/Ueq(min) Range         5.0 Ratio
PLAT222_ALERT_3_C NonSolvent Resd 1  H   Uiso(max)/Uiso(min) Range         5.7 Ratio
PLAT250_ALERT_2_C Large U3/U1 Ratio for Average U(i,j) Tensor ....         2.4 Note
PLAT340_ALERT_3_C Low Bond Precision on  C-C Bonds ..............      0.00476 Ang.
PLAT416_ALERT_2_C Short Intra D-H..H-D       H67       ..H71      .        1.91 Ang.
                                                       x,y,z  =      1_555 Check
PLAT906_ALERT_3_C Large K Value in the Analysis of Variance ......       3.803 Check
PLAT911_ALERT_3_C Missing FCF Refl Between Thmin & STh/L=    0.595           5 Report
PLAT977_ALERT_2_C Check Negative Difference Density on H71        .       -0.34 eA-3
```

Alert level G

```
PLAT003_ALERT_2_G Number of Uiso or Uij Restrained non-H Atoms ...          18 Report
PLAT007_ALERT_5_G Number of Unrefined Donor-H Atoms .............            5 Report
PLAT042_ALERT_1_G Calc. and Reported MoietyFormula Strings  Differ     Please Check
PLAT154_ALERT_1_G The s.u.'s on the Cell Angles are Equal ..(Note)       0.002 Degree
PLAT177_ALERT_4_G The CIF-Embedded .res File Contains DELU Records           1 Report
PLAT186_ALERT_4_G The CIF-Embedded .res File Contains ISOR Records           4 Report
PLAT301_ALERT_3_G Main Residue  Disorder .............(Resd  1  )           9% Note
PLAT413_ALERT_2_G Short Inter XH3 .. XHn     H43B      ..H12B     .        1.98 Ang.
                                                    -1+x,y,z  =      1_455 Check
PLAT606_ALERT_4_G Solvent Accessible VOID(S) in Structure .......            ! Info
PLAT720_ALERT_4_G Number of Unusual/Non-Standard Labels ..........           3 Note
PLAT790_ALERT_4_G Centre of Gravity not Within Unit Cell: Resd.  #           3 Note
              C2 H4 Cl2
PLAT860_ALERT_3_G Number of Least-Squares Restraints .............         150 Note
PLAT869_ALERT_4_G ALERTS Related to the Use of SQUEEZE  Suppressed           ! Info
PLAT883_ALERT_1_G No Info/Value for _atom_sites_solution_primary .     Please Do !
PLAT909_ALERT_3_G Percentage of I>2sig(I) Data at Theta(Max) Still         67% Note
PLAT933_ALERT_2_G Number of HKL-OMIT Records in Embedded .res File           3 Note
PLAT941_ALERT_3_G Average HKL Measurement Multiplicity ...........         1.9 Low
PLAT978_ALERT_2_G Number C-C Bonds with Positive Residual Density.           3 Info
```

```
 0 ALERT level A = Most likely a serious problem - resolve or explain
 4 ALERT level B = A potentially serious problem, consider carefully
 9 ALERT level C = Check. Ensure it is not caused by an omission or oversight
18 ALERT level G = General information/check it is not something unexpected

 3 ALERT type 1 CIF construction/syntax error, inconsistent or missing data
12 ALERT type 2 Indicator that the structure model may be wrong or deficient
 9 ALERT type 3 Indicator that the structure quality may be low
 6 ALERT type 4 Improvement, methodology, query or suggestion
 1 ALERT type 5 Informative message, check
```

It is advisable to attempt to resolve as many as possible of the alerts in all categories. Often the minor alerts point to easily fixed oversights, errors and omissions in your CIF or refinement strategy, so attention to these fine details can be worthwhile. In order to resolve some of the more serious problems it may be necessary to carry out additional measurements or structure refinements. However, the purpose of your study may justify the reported deviations and the more serious of these should normally be commented upon in the discussion or experimental section of a paper or in the "special_details" fields of the CIF. checkCIF was carefully designed to identify outliers and unusual parameters, but every test has its limitations and alerts that are not important in a particular case may appear. Conversely, the absence of alerts does not guarantee there are no aspects of the results needing attention. It is up to the individual to critically assess their own results and, if necessary, seek expert advice.

Publication of your CIF in IUCr journals

A basic structural check has been run on your CIF. These basic checks will be run on all CIFs submitted for publication in IUCr journals (*Acta Crystallographica*, *Journal of Applied Crystallography*, *Journal of Synchrotron Radiation*); however, if you intend to submit to *Acta Crystallographica Section C* or *E* or *IUCrData*, you should make sure that full publication checks are run on the final version of your CIF prior to submission.

Publication of your CIF in other journals

Please refer to the *Notes for Authors* of the relevant journal for any special instructions relating to CIF submission.

PLATON version of 18/05/2022; check.def file version of 17/05/2022

Datablock exp_1516_sq_sq - ellipsoid plot

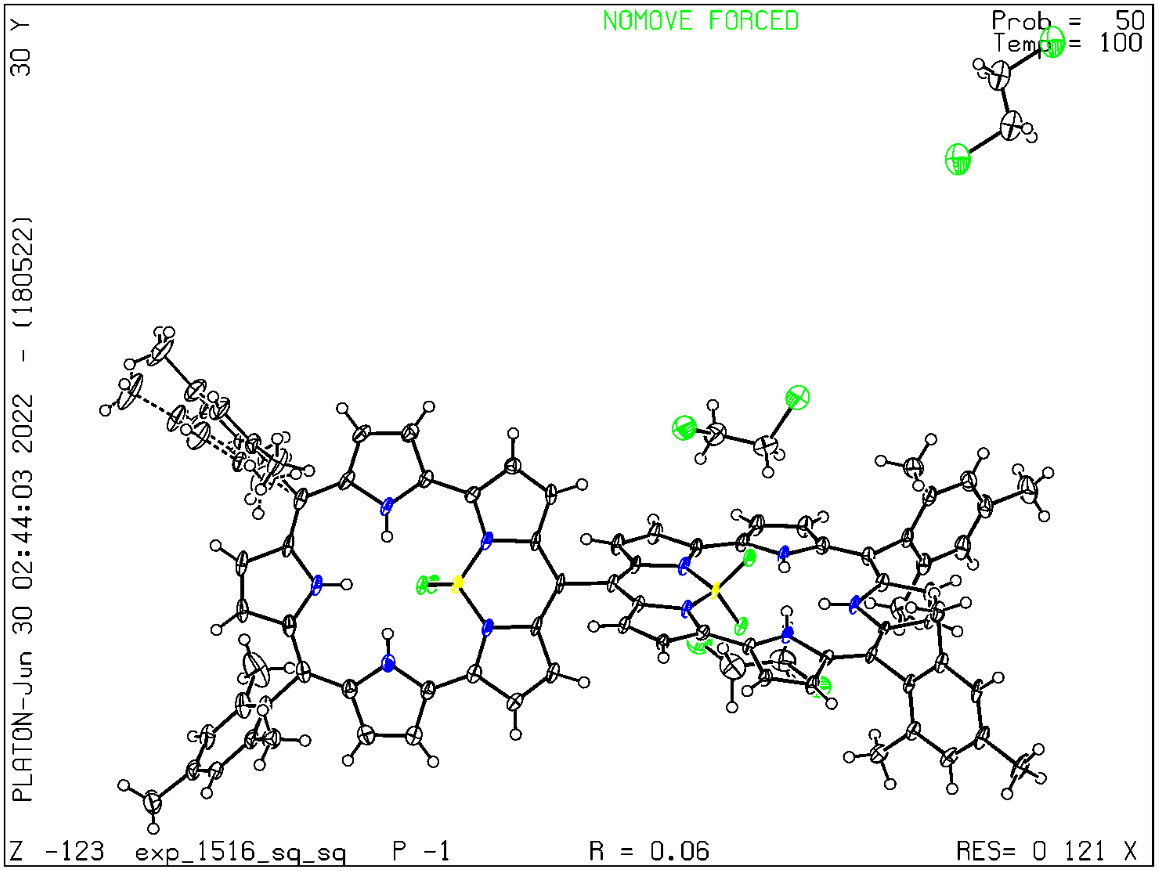

Supplement: Supplementary file 11 — Supplementary Data 8 [file 42004_2023_822_MOESM11_ESM.pdf]
