# Supplementary material for: Aromatic-bridged and meso-meso-linked BF2-smaragdyrin dimers exhibit fast decays in polar solvents by symmetry-breaking charge transfer
Source: Commun Chem. 2023 Feb 9;6:25. doi: 10.1038/s42004-023-00822-8 (PMC9911704; doi:10.1038/s42004-023-00822-8)
Supplement: Supplementary file 12 — Supplementary Data 9 [file 42004_2023_822_MOESM12_ESM.pdf]

Supplementary Data **9. Optimized atomic coordinates**

Supplementary Table 1. Atomic coordinates of the optimized structure of **12**.

Center Number	Atomic Number	Atomic Type	Coordinates (Angstroms) X	Y	Z
1	9	0	-4.872798	-0.084735	11.740604
2	9	0	-2.924673	0.243775	10.603518
3	9	0	1.248633	-2.408155	18.430727
4	9	0	0.427582	-0.473165	19.312389
5	7	0	-5.473708	0.629122	8.465355
6	7	0	-2.952575	-1.433351	12.357326
7	7	0	-4.284721	-1.912673	9.667339
8	7	0	-4.468656	2.487027	10.677167
9	7	0	3.222768	-0.606647	18.916264
10	7	0	-3.038366	0.998931	12.904038
11	7	0	0.916866	-0.524671	16.936650
12	7	0	-1.024969	-1.780206	17.871553
13	7	0	-0.311160	-2.846542	20.623910
14	7	0	2.498330	-2.032967	21.516113
15	6	0	-5.811574	1.906885	7.988712
16	6	0	-5.008665	-3.863358	8.842210
17	6	0	-5.716874	-0.336259	7.473688
18	6	0	-6.214350	1.723005	6.627612
19	6	0	-2.220679	-1.635677	13.521133
20	6	0	-5.568660	-1.727470	7.549188
21	6	0	-3.860679	-2.889061	10.540724
22	6	0	-6.158310	0.389255	6.321484
23	6	0	-5.239771	3.372500	9.954948
24	6	0	-2.521294	-3.652516	12.546469
25	6	0	2.052776	-2.852175	22.567112

26	6	0	3.175277	0.149568	17.766527
27	6	0	−4.124765	3.012847	11.902399
28	6	0	−2.311751	0.731260	14.057973
29	6	0	0.184801	−0.323846	15.772638
30	6	0	−3.026954	−2.057903	16.825688
31	6	0	−4.319864	−4.123684	10.015576
32	6	0	2.139326	0.105120	16.798239
33	6	0	−2.846256	2.927193	14.084010
34	6	0	−4.987709	−2.458572	8.614648
35	6	0	−7.454305	−2.857067	6.300187
36	6	0	−1.709292	−1.538420	16.686550
37	6	0	0.321370	−6.066510	25.432869
38	6	0	−1.569439	−2.843907	20.064977
39	6	0	−3.150599	−2.657511	11.746980
40	6	0	3.201598	−3.100995	23.383678
41	6	0	−1.940234	−3.026731	13.627809
42	6	0	4.318427	0.988332	17.791146
43	6	0	−3.129411	−2.600169	18.088072
44	6	0	−6.380216	4.321274	7.966220
45	6	0	−5.387295	4.525729	10.776192
46	6	0	0.984366	0.439511	14.876511
47	6	0	2.176460	0.711883	15.511459
48	6	0	−0.352456	−3.254776	21.940002
49	6	0	−8.440102	−2.392363	7.349585
50	6	0	−6.082368	−2.537445	6.389041
51	6	0	3.855596	−1.715606	21.693541
52	6	0	−5.688990	−3.740903	4.322371
53	6	0	0.548729	−5.447406	24.199703
54	6	0	0.044486	−3.938161	26.496532
55	6	0	−1.121135	−0.824899	15.626771
56	5	0	−3.482228	−0.072149	11.908036

57	6	0	−2.459835	−3.278031	21.079881
58	6	0	−2.190722	1.944170	14.792618
59	6	0	−5.548201	5.238358	7.286675
60	6	0	−3.715967	−2.658481	5.449488
61	6	0	4.333804	−0.295591	19.670730
62	6	0	0.764164	−3.347727	22.806773
63	6	0	−7.042786	−4.072581	4.218345
64	6	0	4.670998	−0.883854	20.914488
65	6	0	5.025038	0.720265	18.952131
66	6	0	−5.191156	−2.987547	5.390461
67	6	0	−7.907434	−3.619105	5.217650
68	6	0	−5.776861	3.133022	8.665924
69	6	0	−4.706710	4.304654	11.962058
70	6	0	−1.878176	−2.429466	18.744103
71	6	0	−1.886570	−0.570928	14.377132
72	6	0	−1.720227	−3.528690	22.223765
73	6	0	−3.375594	2.339474	12.901080
74	6	0	4.273690	−2.425571	22.863836
75	6	0	0.069048	−5.331384	26.594801
76	5	0	0.386499	−1.269975	18.161059
77	6	0	−6.127375	6.337467	6.645160
78	6	0	−4.048758	5.046527	7.233240
79	6	0	−7.775739	4.528573	7.994356
80	6	0	6.041019	−0.567071	21.451529
81	6	0	0.529357	−4.037340	24.124064
82	6	0	−7.550400	−4.918078	3.073374
83	6	0	6.205303	0.452386	22.415001
84	6	0	0.273580	−3.276407	25.284964
85	6	0	−8.700240	3.568238	8.709955
86	6	0	5.022890	1.238361	22.937433
87	6	0	−8.311616	5.646812	7.345145

88	6	0	-0.146612	-6.023830	27.920539
89	6	0	0.814719	-6.294895	22.975463
90	6	0	-7.506728	6.564366	6.666135
91	6	0	7.489502	0.743020	22.886729
92	6	0	-8.103509	7.779702	5.995075
93	6	0	0.234235	-1.764598	25.242134
94	6	0	7.031044	-2.368679	19.944843
95	6	0	7.167711	-1.274415	20.980226
96	6	0	9.989395	0.358270	22.991237
97	6	0	8.432904	-0.955470	21.485705
98	6	0	8.616975	0.048073	22.440067
99	1	0	-3.895485	-0.977194	9.685897
100	1	0	-3.999960	1.673600	10.295547
101	1	0	2.655890	-1.439141	19.029158
102	1	0	0.525647	-2.790867	20.055102
103	1	0	-5.515980	-4.578497	8.210729
104	1	0	-6.483968	2.533851	5.966174
105	1	0	-6.374958	-0.071078	5.368235
106	1	0	-2.492779	-4.707659	12.311049
107	1	0	-3.790709	-2.019686	16.062373
108	1	0	-4.190877	-5.086560	10.490447
109	1	0	-2.938665	3.971217	14.350578
110	1	0	0.340657	-7.153631	25.485183
111	1	0	3.194809	-3.749090	24.248257
112	1	0	-1.372718	-3.484217	14.425317
113	1	0	4.553504	1.739162	17.049333
114	1	0	-3.991804	-3.090407	18.518823
115	1	0	-5.970104	5.395895	10.510397
116	1	0	0.684804	0.740153	13.882868
117	1	0	3.017245	1.262176	15.111706
118	1	0	-8.106671	-2.653266	8.360104

119	1	0	−9.422405	−2.846987	7.187829
120	1	0	−8.567658	−1.303352	7.328481
121	1	0	−4.998640	−4.075836	3.550316
122	1	0	−0.160460	−3.346216	27.386618
123	1	0	−3.533994	−3.345559	20.975779
124	1	0	−1.673443	2.047619	15.735567
125	1	0	−3.547039	−1.580282	5.548956
126	1	0	−3.232034	−3.135573	6.309879
127	1	0	−3.204438	−3.000379	4.544446
128	1	0	5.925416	1.209402	19.295441
129	1	0	−8.966209	−3.863888	5.155462
130	1	0	−4.659865	4.967584	12.815010
131	1	0	−2.094904	−3.840001	23.188292
132	1	0	5.289217	−2.430217	23.232914
133	1	0	−5.481947	7.034915	6.114109
134	1	0	−3.782532	4.042865	6.883474
135	1	0	−3.593036	5.168202	8.223226
136	1	0	−3.586849	5.775300	6.559983
137	1	0	−8.604254	−4.711478	2.858521
138	1	0	−7.469297	−5.989185	3.302836
139	1	0	−6.976201	−4.739907	2.157652
140	1	0	−8.358868	3.364436	9.730671
141	1	0	−9.714976	3.974556	8.765265
142	1	0	−8.755309	2.601695	8.194405
143	1	0	−9.388452	5.802800	7.372456
144	1	0	−0.728378	−5.401058	28.608228
145	1	0	0.809289	−6.247497	28.413219
146	1	0	−0.674945	−6.975359	27.795352
147	1	0	0.891379	−7.353104	23.243798
148	1	0	1.745906	−6.002236	22.477295
149	1	0	0.013364	−6.193075	22.234128

150	1	0	-9.161273	7.627280	5.756491
151	1	0	-8.039202	8.664224	6.643038
152	1	0	-7.577857	8.023414	5.065089
153	1	0	-0.109619	-1.357755	26.198174
154	1	0	1.224682	-1.341716	25.035379
155	1	0	-0.436508	-1.400161	24.456220
156	1	0	10.103016	1.426320	23.207264
157	1	0	10.778397	0.068536	22.289289
158	1	0	-5.502041	0.401634	9.454357
159	1	0	1.866441	-1.420927	21.009369
160	1	0	10.173614	-0.182190	23.929583
161	1	0	9.297513	-1.506845	21.120851
162	1	0	7.610697	1.536288	23.622272
163	1	0	5.356268	2.065079	23.572318
164	1	0	4.422992	1.656158	22.121337
165	1	0	4.352538	0.607600	23.533424
166	1	0	7.987630	-2.876036	19.786058
167	1	0	6.293512	-3.120420	20.247721
168	1	0	6.698058	-1.969199	18.979590

Supplementary Table 2. Atomic coordinates of the optimized structure of **6b**.

Center Number	Atomic Number	Atomic Type	Coordinates (Angstroms) X	Y	Z
1	9	0	12.395368	0.540255	13.245736
2	9	0	10.750780	14.014316	13.981356
3	7	0	9.932723	-0.593118	13.092568
4	1	0	10.944369	-0.534211	13.104369
5	7	0	12.109082	15.167976	11.906620

6	1	0	11.500573	15.116179	12.716673
7	7	0	10.757629	2.279922	13.667936
8	7	0	11.908570	-2.637421	14.199320
9	7	0	12.098821	12.293617	12.942136
10	6	0	8.820379	-4.170971	12.520427
11	6	0	12.297132	17.644814	11.672761
12	6	0	11.518605	-4.754544	13.498204
13	1	0	11.047692	-5.591538	13.002857
14	6	0	10.959795	-3.436862	13.539157
15	6	0	12.441751	11.536356	10.832660
16	1	0	12.597515	11.538926	9.762535
17	6	0	9.576336	1.865477	13.083698
18	6	0	9.717070	-3.066314	13.010769
19	6	0	12.962443	6.587336	13.664734
20	1	0	13.804157	6.044256	13.245122
21	6	0	11.890239	8.713465	14.180141
22	6	0	12.080790	18.041093	12.998551
23	6	0	9.146183	0.522797	12.915609
24	6	0	9.207143	-1.748611	12.896460
25	7	0	12.001224	17.240159	14.142223
26	6	0	8.857803	3.023637	12.678640
27	1	0	7.903632	3.012991	12.169786
28	6	0	12.414752	14.039154	11.175290
29	6	0	12.476267	18.734890	10.650097
30	6	0	8.753832	-4.469024	11.141377
31	6	0	12.404927	16.314261	11.193833
32	6	0	12.969979	7.979529	13.659246
33	1	0	13.828341	8.509928	13.257777
34	6	0	10.796887	3.669020	13.647683
35	6	0	9.611440	4.129611	13.008709
36	1	0	9.370684	5.165075	12.816604

37	6	0	7.122288	-6.233140	11.595276
38	6	0	12.093357	10.904626	12.975655
39	6	0	12.312285	12.701228	11.638428
40	6	0	11.874078	4.385172	14.203189
41	6	0	11.876210	5.868709	14.193764
42	6	0	11.897460	10.197654	14.176252
43	6	0	12.293280	10.434803	11.647266
44	1	0	12.310423	9.395939	11.351281
45	5	0	11.849151	1.402364	14.279675
46	6	0	7.911302	-5.496946	10.706634
47	1	0	7.871950	-5.728491	9.643726
48	6	0	11.907391	19.387597	13.456035
49	1	0	11.878396	20.246946	12.801746
50	6	0	8.036128	-4.906057	13.435042
51	6	0	11.389705	19.121470	9.834910
52	6	0	13.869849	20.370678	9.529723
53	1	0	14.838433	20.853442	9.413549
54	6	0	7.201667	-5.921808	12.954616
55	1	0	6.597291	-6.483532	13.664501
56	6	0	13.728613	19.366754	10.494795
57	6	0	8.079656	-4.617875	14.919699
58	1	0	9.041971	-4.907514	15.359063
59	1	0	7.294085	-5.169553	15.445377
60	1	0	7.943933	-3.550982	15.127926
61	5	0	11.879917	13.178942	14.166484
62	6	0	9.583733	-3.706473	10.132561
63	1	0	9.276833	-2.655655	10.072262
64	1	0	9.481351	-4.143837	9.134605
65	1	0	10.646928	-3.710989	10.398172
66	6	0	12.913083	14.490892	9.928343
67	1	0	13.286986	13.851079	9.140936

68	6	0	11.574494	20.133248	8.887614
69	1	0	10.730423	20.433073	8.269093
70	6	0	12.807536	20.768892	8.714898
71	6	0	14.916618	18.982791	11.349421
72	1	0	14.774247	19.280604	12.395235
73	1	0	15.828565	19.466634	10.985991
74	1	0	15.081857	17.899871	11.347811
75	6	0	12.906346	15.876133	9.936806
76	1	0	13.264046	16.534831	9.158645
77	6	0	6.192987	-7.314623	11.094959
78	1	0	5.231544	-6.894470	10.770077
79	1	0	5.978002	-8.052028	11.875492
80	1	0	6.619637	-7.844321	10.236077
81	6	0	10.032665	18.468634	9.976689
82	1	0	10.067317	17.406932	9.705164
83	1	0	9.296421	18.956488	9.330479
84	1	0	9.666261	18.519382	11.008293
85	6	0	12.990196	21.836430	7.661037
86	1	0	13.822103	22.504537	7.907380
87	1	0	12.087337	22.446546	7.547370
88	1	0	13.206783	21.394313	6.679174
89	9	0	11.338631	0.548507	15.265070
90	9	0	12.998457	14.067869	14.329608
91	7	0	13.915515	-0.570079	15.221452
92	1	0	13.349510	-0.515698	14.382641
93	7	0	11.740792	15.149744	16.429948
94	1	0	12.281490	15.047657	15.582072
95	7	0	12.969083	2.290689	14.821168
96	7	0	11.716286	12.301407	15.410486
97	6	0	15.029186	-4.139350	15.844828
98	6	0	11.637586	17.632295	16.624031

99	6	0	12.727132	-4.747399	14.141500
100	1	0	13.408247	-5.577813	14.260535
101	6	0	12.992511	-3.425112	14.622713
102	6	0	11.396339	11.538500	17.524859
103	1	0	11.255700	11.539597	18.597139
104	6	0	14.085772	1.888980	15.528858
105	6	0	14.117367	-3.042826	15.364062
106	6	0	10.797184	6.602863	14.715977
107	1	0	9.951902	6.072723	15.144417
108	6	0	11.822936	18.023819	15.289424
109	6	0	14.475617	0.550071	15.793435
110	6	0	14.482360	-1.721253	15.724898
111	6	0	14.773773	3.055828	15.961404
112	1	0	15.693281	3.057031	16.530626
113	6	0	11.452228	14.041229	17.190589
114	6	0	11.452832	18.736164	17.634330
115	6	0	14.701046	-4.878763	17.001537
116	6	0	11.536661	16.313870	17.136875
117	6	0	10.803260	7.994966	14.707393
118	1	0	9.950982	8.537681	15.105318
119	6	0	12.938682	3.679843	14.795475
120	6	0	14.072759	4.154463	15.512989
121	1	0	14.314334	5.194803	15.675605
122	6	0	16.750564	-6.189865	16.753279
123	6	0	11.712105	10.910771	15.375345
124	6	0	11.521149	12.704197	16.721336
125	6	0	11.525852	10.438938	16.704888
126	1	0	11.507064	9.399290	16.997834
127	6	0	15.568270	-5.888406	17.433640
128	1	0	15.310197	-6.453369	18.327485
129	6	0	11.765073	19.378210	14.817132

130	1	0	11.599653	20.230445	15.459943
131	6	0	16.221745	-4.425771	15.144442
132	6	0	12.566739	19.232011	18.344631
133	6	0	15.460238	-1.298455	16.667796
134	1	0	16.063087	-1.966289	17.266316
135	6	0	10.026093	20.285445	18.832072
136	1	0	9.034222	20.693824	19.017075
137	6	0	15.454373	0.086541	16.707947
138	1	0	16.048217	0.717760	17.354568
139	6	0	17.055400	-5.448267	15.608258
140	1	0	17.967654	-5.670926	15.057742
141	6	0	10.169052	19.267961	17.881791
142	6	0	16.604338	-3.656127	13.899842
143	1	0	15.797571	-3.660975	13.158273
144	1	0	17.496329	-4.086717	13.434289
145	1	0	16.818395	-2.605298	14.128058
146	6	0	13.433640	-4.601702	17.779871
147	1	0	13.324271	-3.535473	18.006203
148	1	0	13.430410	-5.152557	18.725631
149	1	0	12.541312	-4.900216	17.216385
150	6	0	11.044307	14.522440	18.460425
151	1	0	10.704425	13.906018	19.281235
152	6	0	12.378018	20.250806	19.284275
153	1	0	13.242329	20.632893	19.824465
154	6	0	11.115698	20.790045	19.546546
155	6	0	8.952069	18.765128	17.136948
156	1	0	9.021556	18.973089	16.062673
157	1	0	8.042032	19.242818	17.512936
158	1	0	8.834803	17.680874	17.241586
159	6	0	11.099319	15.906474	18.431454
160	1	0	10.817886	16.584711	19.223847

161	6	0	17.685579	-7.266398	17.253775
162	1	0	18.464686	-6.846530	17.904331
163	1	0	18.194739	-7.772101	16.426063
164	1	0	17.150261	-8.023899	17.835901
165	6	0	13.955932	18.685656	18.101482
166	1	0	14.013025	17.615327	18.332076
167	1	0	14.692866	19.203627	18.722948
168	1	0	14.256375	18.800612	17.053573
169	6	0	10.931461	21.867654	20.589858
170	1	0	10.069574	22.503707	20.361609
171	1	0	11.816193	22.509167	20.664445
172	1	0	10.760843	21.434146	21.584575
173	6	0	7.849385	0.052241	12.590666
174	1	0	6.982372	0.679349	12.434822
175	6	0	7.883653	-1.333201	12.580655
176	1	0	7.055768	-2.005037	12.405068
177	1	0	12.334160	16.287400	14.143884
178	1	0	11.646926	-1.783074	14.681426

Supplementary Table 3. Atomic coordinates of the optimized structure of **6c**

Center Number	Atomic Number	Atomic Type	Coordinates (Angstroms) X	Y	Z
1	9	0	6.333131	18.713959	8.737366
2	9	0	4.303949	18.084749	7.908929
3	7	0	4.051691	18.298593	11.244097
4	1	0	4.865564	18.465049	10.660450
5	7	0	5.366076	16.165624	9.504224
6	1	0	4.744110	16.909173	9.208541

7	7	0	4.213777	20.513994	9.143073
8	1	0	4.046004	19.526749	8.987466
9	7	0	5.632030	19.633316	6.603374
10	7	0	6.296229	17.238078	6.813096
11	6	0	4.323887	14.848954	12.901287
12	6	0	3.916846	17.131553	12.015670
13	6	0	3.336255	19.353701	11.835635
14	6	0	3.247005	20.689716	11.425924
15	6	0	3.747514	21.239215	10.218481
16	6	0	4.504003	15.876247	11.815827
17	6	0	2.563321	21.651004	12.360819
18	6	0	3.028341	17.470684	13.086061
19	1	0	2.682193	16.763112	13.825666
20	6	0	3.330405	13.853735	12.773067
21	6	0	6.055172	14.299139	10.529000
22	1	0	6.211207	13.555445	11.297171
23	6	0	2.683999	18.791443	12.979608
24	1	0	2.009673	19.343833	13.618162
25	6	0	4.973681	13.895659	15.032664
26	1	0	5.617266	13.915788	15.910316
27	6	0	5.153049	14.868869	14.043670
28	6	0	3.995685	12.903355	14.925734
29	6	0	1.218624	22.019577	12.138239
30	6	0	3.269147	22.200927	13.452716
31	6	0	5.259525	15.467489	10.687909
32	6	0	3.182125	12.904274	13.789488
33	1	0	2.408209	12.145474	13.689355
34	6	0	6.611595	14.322088	9.259782
35	1	0	7.301024	13.601206	8.842235
36	6	0	6.227732	15.919438	14.217591
37	1	0	6.871704	15.987510	13.333941

38	1	0	6.858808	15.689720	15.081778
39	1	0	5.796326	16.915546	14.373629
40	6	0	0.605130	22.920712	13.014755
41	1	0	-0.432778	23.198124	12.839727
42	6	0	6.175873	15.503234	8.608956
43	6	0	4.647427	21.345037	8.134371
44	6	0	2.421864	13.803601	11.564957
45	1	0	1.933373	14.768163	11.387225
46	1	0	1.642177	13.046534	11.694513
47	1	0	2.979983	13.558522	10.653529
48	6	0	3.895093	22.608834	9.863022
49	1	0	3.649224	23.439218	10.509256
50	6	0	0.432530	21.455960	10.975375
51	1	0	0.838218	21.793861	10.014445
52	1	0	-0.615068	21.768511	11.025913
53	1	0	0.460455	20.360542	10.963059
54	6	0	2.617946	23.104036	14.300025
55	1	0	3.170441	23.530846	15.135117
56	6	0	1.284895	23.473693	14.103390
57	6	0	6.517095	15.968852	7.311729
58	6	0	5.224788	20.917394	6.909840
59	6	0	4.443708	22.670973	8.592046
60	1	0	4.721819	23.564516	8.050259
61	6	0	6.775864	17.290467	5.509740
62	6	0	6.148749	19.629539	5.313446
63	6	0	6.708969	18.470283	4.745251
64	6	0	6.060579	20.955526	4.803918
65	1	0	6.395067	21.267444	3.825070
66	6	0	3.840214	11.846003	15.994086
67	1	0	4.481631	10.977626	15.791450
68	1	0	2.809166	11.480744	16.049863

69	1	0	4.118078	12.229569	16.981738
70	6	0	4.714120	21.839470	13.718358
71	1	0	4.816755	20.793740	14.032409
72	1	0	5.132176	22.468329	14.510550
73	1	0	5.332705	21.964027	12.822788
74	6	0	5.495250	21.743063	5.783879
75	1	0	5.273187	22.799758	5.723787
76	6	0	7.147576	15.201784	6.293780
77	1	0	7.423849	14.160287	6.385897
78	6	0	7.292627	16.005188	5.183141
79	1	0	7.709109	15.728048	4.225632
80	5	0	5.667465	18.426208	7.539953
81	6	0	0.591804	24.422931	15.053065
82	1	0	-0.190679	24.997745	14.546060
83	1	0	1.298158	25.131451	15.498970
84	1	0	0.112141	23.880250	15.878870
85	6	0	6.917890	18.913617	0.981483
86	1	0	6.270167	19.244240	0.174319
87	6	0	6.429249	18.901737	2.284504
88	1	0	5.401456	19.195857	2.476100
89	6	0	7.239397	18.497510	3.359708
90	6	0	8.561624	18.115248	3.075615
91	1	0	9.213788	17.813134	3.889657
92	6	0	9.049030	18.129968	1.772057
93	1	0	10.069935	17.809713	1.583316
94	6	0	8.237662	18.525440	0.695062
95	9	0	10.659257	18.714242	-8.737203
96	9	0	12.688333	18.084455	-7.908942
97	7	0	12.940490	18.298544	-11.244082
98	1	0	12.126707	18.465136	-10.660348
99	7	0	11.625659	16.165732	-9.504365

100	1	0	12.247826	16.909098	-9.208644
101	7	0	12.779050	20.513793	-9.142843
102	1	0	12.946594	19.526492	-8.987338
103	7	0	11.360705	19.633216	-6.603159
104	7	0	10.695907	17.238162	-6.813084
105	6	0	12.667309	14.849154	-12.901630
106	6	0	13.074986	17.131555	-12.015791
107	6	0	13.656135	19.353541	-11.835564
108	6	0	13.745722	20.689499	-11.425737
109	6	0	13.245418	21.239004	-10.218212
110	6	0	12.487527	15.876374	-11.816044
111	6	0	14.429549	21.650713	-12.360602
112	6	0	13.963518	17.470573	-13.086198
113	1	0	14.309450	16.762993	-13.825896
114	6	0	13.660604	13.853716	-12.773619
115	6	0	10.936046	14.299517	-10.529284
116	1	0	10.779782	13.555938	-11.297519
117	6	0	14.308189	18.791238	-12.979633
118	1	0	14.982610	19.343527	-13.618173
119	6	0	12.017094	13.896210	-15.033035
120	1	0	11.373417	13.916561	-15.910614
121	6	0	11.838029	14.869355	-14.043916
122	6	0	12.994900	12.903702	-14.926317
123	6	0	15.774327	22.019016	-12.138062
124	6	0	13.723780	22.200833	-13.452437
125	6	0	11.731971	15.467687	-10.688123
126	6	0	13.808584	12.904335	-13.790155
127	1	0	14.582357	12.145367	-13.690189
128	6	0	10.379711	14.322473	-9.260027
129	1	0	9.690133	13.601716	-8.842511
130	6	0	10.763531	15.920151	-14.217607

131	1	0	10.119643	15.988222	-13.333894
132	1	0	10.132342	15.690669	-15.081774
133	1	0	11.195112	16.916199	-14.373540
134	6	0	16.387952	22.920088	-13.014552
135	1	0	17.425921	23.197289	-12.839555
136	6	0	10.815746	15.503458	-8.609118
137	6	0	12.345652	21.344843	-8.134040
138	6	0	14.569268	13.803280	-11.565614
139	1	0	15.057998	14.767718	-11.387863
140	1	0	15.348769	13.046046	-11.695313
141	1	0	14.011192	13.558261	-10.654145
142	6	0	13.098181	22.608625	-9.862616
143	1	0	13.344204	23.439012	-10.508788
144	6	0	16.560371	21.455184	-10.975268
145	1	0	16.154859	21.793196	-10.014302
146	1	0	17.608049	21.767451	-11.025897
147	1	0	16.532148	20.359774	-10.962949
148	6	0	14.375114	23.103870	-14.299722
149	1	0	13.822662	23.530828	-15.134766
150	6	0	15.708242	23.473264	-14.103124
151	6	0	10.474702	15.969039	-7.311830
152	6	0	11.768249	20.917223	-6.909521
153	6	0	12.549673	22.670774	-8.591595
154	1	0	12.271810	23.564332	-8.049705
155	6	0	10.216338	17.290544	-5.509702
156	6	0	10.844037	19.629444	-5.313211
157	6	0	10.283553	18.470272	-4.745105
158	6	0	10.932546	20.955364	-4.803565
159	1	0	10.598167	21.267271	-3.824677
160	6	0	13.150055	11.846426	-15.994790
161	1	0	12.509059	10.977827	-15.791772

162	1	0	14.181190	11.481504	-16.051216
163	1	0	12.871455	12.229926	-16.982258
164	6	0	12.278728	21.839663	-13.718040
165	1	0	12.175881	20.793961	-14.032116
166	1	0	11.860767	22.468625	-14.510199
167	1	0	11.660197	21.964313	-12.822445
168	6	0	11.498037	21.742851	-5.783471
169	1	0	11.720360	22.799486	-5.723289
170	6	0	9.844074	15.202030	-6.293928
171	1	0	9.567536	14.160611	-6.386133
172	6	0	9.699263	16.005365	-5.183208
173	1	0	9.282748	15.728237	-4.225710
174	5	0	11.324916	18.426212	-7.539856
175	6	0	16.401475	24.422422	-15.052776
176	1	0	17.183987	24.997170	-14.545739
177	1	0	15.695213	25.131002	-15.498729
178	1	0	16.881133	23.879683	-15.878545
179	6	0	10.074775	18.913317	-0.981290
180	1	0	10.722582	19.243715	-0.174102
181	6	0	10.563404	18.901436	-2.284317
182	1	0	11.591265	19.195326	-2.475895
183	6	0	9.753147	18.497503	-3.359552
184	6	0	8.430829	18.115540	-3.075483
185	1	0	7.778584	17.813664	-3.889548
186	6	0	7.943438	18.130260	-1.771920
187	1	0	6.922455	17.810242	-1.583201
188	6	0	8.754909	18.525435	-0.694896
